# Reactivity of ethyl nitrosoacrylate toward pyrrole, indole and pyrrolo[3,2-*c*]carbazole: an experimental and theoretical study

**DOI:** 10.3389/fchem.2023.1229669

**Published:** 2023-08-08

**Authors:** Alice Benzi, Susana M. M. Lopes, Sandra C. C. Nunes, Gianluca Giorgi, Lara Bianchi, Cinzia Tavani, Alberto A. C. C. Pais, Giovanni Petrillo, Teresa M. V. D. Pinho e Melo

**Affiliations:** ^1^ Department of Chemistry and Industrial Chemistry, University of Genova, Genoa, Italy; ^2^ Coimbra Chemistry Centre-Institute of Molecular Sciences, Department of Chemistry, University of Coimbra, Coimbra, Portugal; ^3^ Department of Biotechnology, Chemistry and Pharmacy, University of Siena, Siena, Italy

**Keywords:** nitrosoalkenes, pyrrolo[3,2-*c*]carbazole, hetero-Diels-Alder reactions, pictet-spengler reaction, DFT calculations

## Abstract

Nitrosoalkenes react with 8-methyl-1,6-dihydropyrrolo[3,2-*c*]carbazole to give both 2- and 3-alkylated products via hetero-Diels-Alder reaction followed by the cycloadduct ring-opening. Quantum chemical calculations, at DFT level of theory, were carried out to investigate the regioselectivity of the cycloaddition of ethyl nitrosoacrylate with 1,6-dihydropyrrolo[3,2-*c*]carbazoles as well as with pyrrole and indole, allowing a more comprehensive analysis of the reactivity pattern of nitrosoalkenes with five-membered heterocycles. Furthermore, theoretical calculations confirmed that ethyl nitrosoacrylate reacts with these heterocycles via a LUMO_heterodiene_-HOMO_dienophile_ controlled cycloaddition. The reactivity of one of the oxime-functionalized 1,6-dihydropyrrolo[3,2-*c*]carbazole was explored and a new hexahydropyrido[4′,3':4,5]pyrrolo[3,2-*c*]carbazole system was obtained in high yield via a one-pot, two-step procedure.

## Graphical Abstract

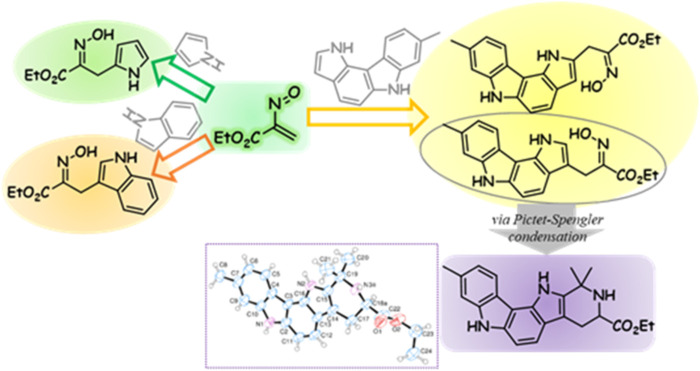

## Introduction

The chemistry of conjugated nitrosoalkenes has been extensively studied for the synthesis and functionalization of a plethora of heterocyclic systems. These reactive intermediates act mainly as electron-deficient heterodienes in inverse electron-demand hetero-Diels-Alder reactions or as Michael-type acceptors in conjugated 1,4-addition reactions (Lopes et al., 2018a; Naumovich et al., 2019; Weinreb, 2019; Soares et al., 2022). In recent years, our research group has explored the reactivity and versatility of conjugated nitrosoalkenes as a synthetic tool to achieve heterocyclic structural diversity (Lopes et al., 2018a). Functionalization of five-membered heterocycles, such as pyrroles, dipyrromethanes, indoles and furans, was achieved by reaction with nitrosoalkenes, including the synthesis of 3-tetrazolyl and 3-triazolyl derivatives (Lopes et al., 2010; Lopes et al., 2011; Nunes et al., 2014; Lopes et al., 2015; Lopes et al., 2016; Alves et al., 2017; Jorda et al., 2017; Lopes et al., 2018b). One-pot methods have also been developed for the synthesis of dipyrromethanes (Pereira et al., 2014; Cardoso et al., 2021), bis(indolyl)methanes (Grosso et al., 2015; Grosso et al., 2017; Grosso et al., 2019), bis(pyrazol-1-yl)methanes (Grosso et al., 2014) and tetrapyrrolic compounds (Lopes and Pinho e Melo, 2020) via two consecutive hetero-Diels-Alder reactions (or conjugated additions) of *in situ* generated nitrosoalkenes with pyrroles, indoles, pyrazoles and dipyrromethanes, respectively. Moreover, tryptophan analogues have been obtained by reducing the oxime moiety of indoles C3-functionalized via hetero-Diels-Alder reactions with nitrosoalkenes. These tryptamine derivatives were used in the synthesis of 3-triazolyl- and 3-tetrazolyl-β-carbolines via Pictet–Spengler condensation followed by an oxidative step. *ß*-Carboline derivatives obtained by this strategy have shown interesting anticancer properties (Panice et al., 2019; Ribeiro et al., 2021; Ribeiro et al., 2022).

**SCHEME 1:**
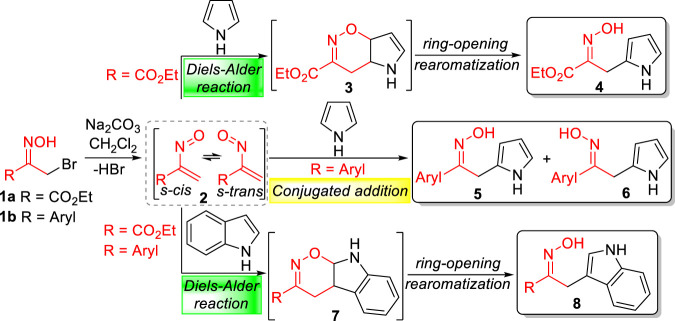
Conjugated 1,4-addition and hetero-Diels-Alder reactions of nitrosoalkenes with heterocycles.

The reactivity of nitrosoalkenes with electron-rich heterocycles is strongly influenced not only by the nitrosoalkene substituents but also by the type of heterocycle (Nunes et al., 2014; Lopes et al., 2016). The pioneer work of Gilchrist and co-workers showed that the reaction of ethyl nitrosoacrylate (**2**, R = CO_2_Et), generated from ethyl bromopyruvate oxime **1a** by action of base, with pyrrole and indole afforded the open chain oximes **4** and **8**, respectively, through hetero-Diels-Alder reactions (Scheme 1) (Gilchrist and Roberts, 1983; Gilchrist and Lemos, 1993). The outcome and mechanistic pathway of the reaction of nitrosoalkenes with pyrrole and pyrrole derivatives depends on the nitrosoalkene substituent, as shown by experimental and theoretical studies (Nunes et al., 2014). In fact, ethyl nitrosoacrylate (**2**, R = CO_2_Et) reacts *via* hetero-Diels-Alder reaction, through the formation of the bicyclic 1,2-oxazine **3** followed by 1,2-oxazine ring-opening with concomitant rearomatization of the pyrrole unit, giving the open chain oxime **4**, as a single isomer. However, aryl nitrosoalkenes **2** (R = Aryl) react with pyrrole by conjugated addition to give two isomeric oximes **5** and **6**. On the other hand, the reaction of both nitrosoalkenes **2** with indole affords open chain oximes as single isomers *via* hetero-Diels-Alder reaction (Lopes et al., 2016). Furthermore, nitrosoalkenes **2** react with pyrrole to give 2-alkylated products, whereas indole undergoes alkylation at the 3-position, as would be expected from the opposite regioselectivity of the hetero-Diels-Alder reaction.

**FIGURE 1:**
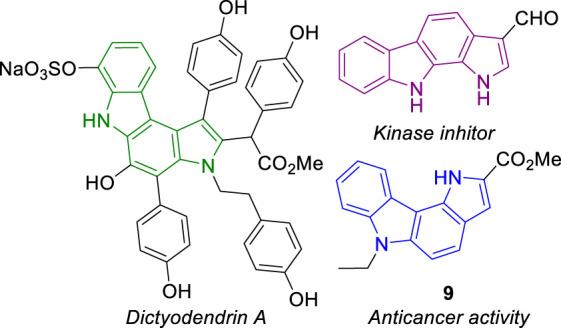
Examples of different pyrrolocarbazoles.

Pyrrolocarbazoles are tetracyclic ring systems containing a pyrrole ring fused to a carbazole unit. Depending on the position of the pyrrole/carbazole ring junction and the relative position of the pyrrole nitrogen to the carbazole moiety, several structural isomers can be found (Giraud et al., 2019). The best known are the pyrrolo[2,3-*c*]carbazoles (Zhang and Ready, 2017), mainly because their core structure is present in the marine natural product dictyodendrin A, and pyrrolo[2,3-*a*]carbazoles for the recognized kinase inhibitory activity of some derivatives (Akué-Gédu et al., 2009; Giraud et al., 2012) (Figure 1). Reports on the synthesis and reactivity of pyrrolo[3,2-*c*]carbazoles are scarce. Recently, however, a few reports describing the synthesis (Benzi et al., 2019), photophysical and biological activity, namely, antioxidant (Bingul et al., 2019), anticancer and antibacterial activity, have been disclosed (Sengul et al., 2016; Saglam et al., 2021; Sinicropi et al., 2021). Compound **9** is an example of a pyrrolo[3,2-*c*]carbazole with high cytotoxicity against human colon cancer HT29 cells (Sengul et al., 2016).

Compounds containing an oxime moiety have found a wide range of biological applications, displaying anti-inflammatory, antimicrobial, antioxidant and anticancer activity (Surowiak et al., 2020). In this context, and following our interest in the chemistry of nitrosoalkenes, we decided to explore the reactivity of nitrosoalkenes towards 8-methyl-1,6-dihydropyrrolo[3,2-*c*]carbazole (**10**) aiming at the synthesis of oxime-functionalized pyrrolo[3,2-*c*]carbazoles. The combination of these two structural elements in a single molecule would lead to new chemical entities with increased interest in terms of potential biological activity and with the possibility of further structural modulation.

## Results and discussion

**SCHEME 2:**
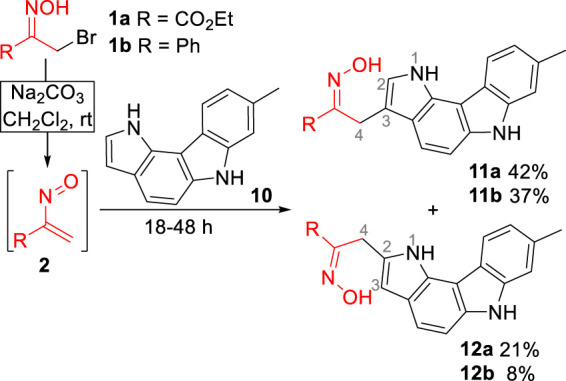
Reactivity of nitrosoalkenes towards 8-methyl-1,6-dihydropyrrolo [3,2-*c*]carbazole.

Initially, the reaction of ethyl nitrosoacrylate (**2a**), generated *in situ* from ethyl bromopyruvate oxime (**1a**) by action of sodium carbonate, with pyrrolo[3,2-*c*]carbazole **10** was explored. The reaction, carried out in dichloromethane at room temperature, gave the 3- and 2-alkylated pyrrolo[3,2-*c*]carbazoles **11a** and **12a**, respectively, in 63% overall yield (Scheme 2). The same reactivity pattern was observed in the reaction of the less activated nitrosoalkene **2b** with **10** affording the open chain oximes **11b** and **12b** in 37% and 8%, respectively (Scheme 2). In both cases, the regioisomeric open chain oximes were isolated as single stereoisomers, indicating that these were formed *via* hetero-Diels-Alder reaction followed by 1,2-oxazine ring-opening and concomitant rearomatization of the pyrrole unit.

The structural assignment of the two regioisomers was established by two-dimensional NMR spectroscopy. From the coupling observed in the COSY spectrum of compound **11a**, it was possible to assign the signals corresponding to the protons H-2 (7.14 ppm), H-1 (10.49 ppm), H-4 (4.15 ppm) and the proton of the hydroxyimino moiety (11.35 ppm). The stereochemistry of the C,N-bond was established by the NOESY spectrum data, in which cross peaks were observed between the NO*H* proton and the H-4 protons, confirming the *trans* orientation of the OH and ester groups. The NOESY spectra of compounds **11b** and **12b** also showed a correlation between the proton of the oxime moiety and H-4 protons (see Supplementary Material), suggesting the *trans* orientation of the OH and ester groups.

**SCHEME 3:**
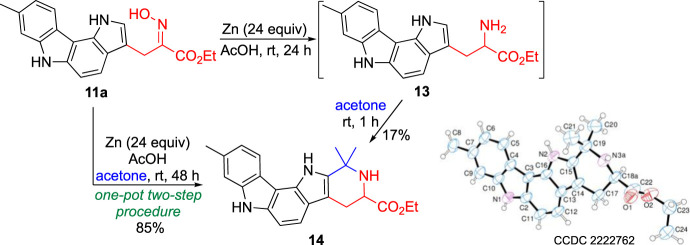
Reactivity of a 3-alkylated pyrrolo[3,2-*c*]carbazole.

Preliminary studies on the reactivity of the new 3-alkylated pyrrolo[3,2-*c*]carbazoles focused on the interconversion of the oxime-amine functional groups. However, the reduction reaction of pyrrolo[3,2-*c*]carbazole **11a**, using zinc in acetic acid at room temperature, led to an unexpected but interesting result. One product was isolated whose ^1^H NMR spectrum features, recorded using acetone-*d*_6_ as solvent, were not those expected for the desired amine **13**. From the analysis of the ^1^H NMR spectrum it was possible to confirm the presence of the pyrrolo[3,2-*c*]carbazole scaffold, signals corresponding to the ester group as well as to an ABX system, as it would be for amine **13**. It was observed that when the product of the reduction reaction was treated with acetone for 1 h, a compound was isolated in 17% yield with a similar ^1^H NMR spectrum but in which the presence of two methyl groups could be observed (Scheme 3).

The structure of this new compound was unambiguously determined by X-ray crystallography as being hexahydropyrido [4′,3':4,5]pyrrolo[3,2-*c*]carbazole **14** (Scheme 3). This heterocyclic compound crystallized with an ethanol and water molecule as colourless plates in the triclinic crystal system within the P-1 space group, showing one molecule of **14**⋅H_2_O⋅C_2_H_5_OH per asymmetric unit. Its molecular structure consists of a five-fused ring system, in which a piperidine ring is fused to the 1,6-dihydropyrrolo[3,2-*c*]carbazole moiety. This six-membered heterocycle contains two methyl groups at C (19) and an ester substituent at C (18). Bond lengths and bond angles are normal. The 1,6-dihydropyrrolo[3,2-*c*]carbazole moiety is almost planar with the largest deviations from the least-squares plane shown by C (9) (0.091 (4)Å), C (7) (0.086 (3)Å) and C (5) (0.081 (3)Å). In the tetrahydropyridine moiety the atoms C (18) and N (3) show a statistical disorder with two different positions with site occupation factors refined to 0.671) (conformer A) and 0.331) (conformer B). Ring puckering analysis (Cremer and Pople, 1975) of the tetrahydropyridine ring shows the following parameters: conformer A: *θ* = 51.27°, *φ* = 32.08°, total puckering amplitudes (*Q*_T_) 0.45 Å; conformer B: *θ* = 48.51°, *φ* = 32.65°, *Q*_T_ 0.57 Å, suggesting a half chair conformation for both the conformations. A network of intra- and intermolecular hydrogen bonding interactions involves the water and ethanol molecules. In particular, an intramolecular H-bond interaction involves O (1 Et)-H ^…^ O (1w) d H ^…^ O (1w) = 1.97 (1)Å and O (1w)-H ^…^ O (2) d H ^…^ O (2) = 2.76 (4)Å, and intermolecular H-bond interactions are N (1)-H ^…^ O (1w) (*x-1, y+1, z*) d H ^…^ O (1w) = 2.021) Å; N (2)-H ^…^ O (1et) (*x-1,y,z*) d H ^…^ O (1w) = 2.051) Å.

The formation of the pentaheterocyclic system **14** can be explained considering the initial reduction of the oxime moiety to give amine **13** followed by its Pictet-Spengler condensation with acetone to give the final product.

The condensation of tryptamines with aldehydes and ketones, known as the Pictet-Spengler reaction, has been extensively explored for the synthesis of ring-fused indole derivatives, including tetrahydro-β-carbolines a core structure of various indole alkaloids (Stöckigt et al., 2011; Maity et al., 2019; Panice et al., 2019; Ribeiro et al., 2022). The mechanism of this transformation has been a research topic of some controversy as two pathways can be considered: the formation of spiroindolenines via the attack of indole’s C3 to the initially formed imine followed by a 1,2-migration/elimination sequence to restore aromaticity, or the direct C2 attack. However, there are several experimental studies where the spiroindolenines, intermediates of Pictet−Spengler-type reactions, were captured (Williams and Unger, 1970; Stöckigt et al., 2011; Chambers et al., 2016; Zheng and You, 2020). Furthermore, in recent years the interrupted Pictet–Spengler reaction is being explored as a strategy for the dearomatisation of indoles (James et al., 2016). Several successful syntheses of spirocyclic indolenine are known resulting from methodologies designed to allow the initial spirocyclisation step but preventing further reaction. The reported synthesis of hexahydropyrido[4′,3':4,5]pyrrolo[3,2-*c*]carbazole **14** is a new entry to Pictet−Spengler-type reactions.

This interesting result justified the optimization of the synthetic procedure, as a one-pot two-step procedure. Thus, a solution of compound **11a** in acetic acid and acetone was treated with zinc powder at room temperature for 48 h. After the neutralization of the reaction medium and purification, the target compound **14** was isolated in 85% yield (Scheme 3).

### Rationalization of the hetero-Diels-Alder reactions outcome

**FIGURE 2:**
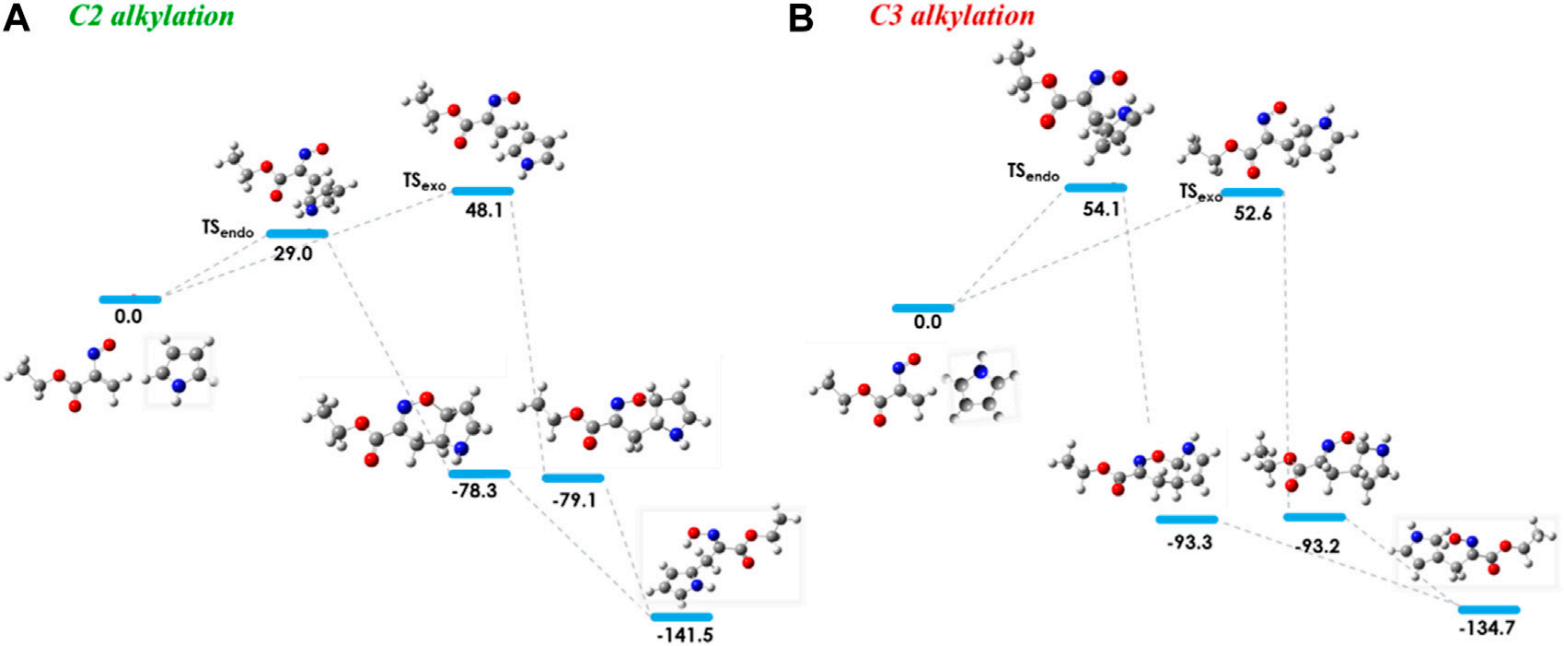
Relative stabilities (ΔE in kJ/mol) of the transition states involved in the hetero-Diels-Alder reaction of ethyl nitrosoacrylate (**2a**) with pyrrole considering the two regioisomers and both *endo* and *exo* approaches, both for C2 alkylation **(A)** and for C3 alkylation **(B)**. All structures were optimized at the B3LYP/6-31G (d,p) level of theory. Color code: grey, carbon; red, oxygen; blue, nitrogen and white, hydrogen.

**FIGURE 3:**
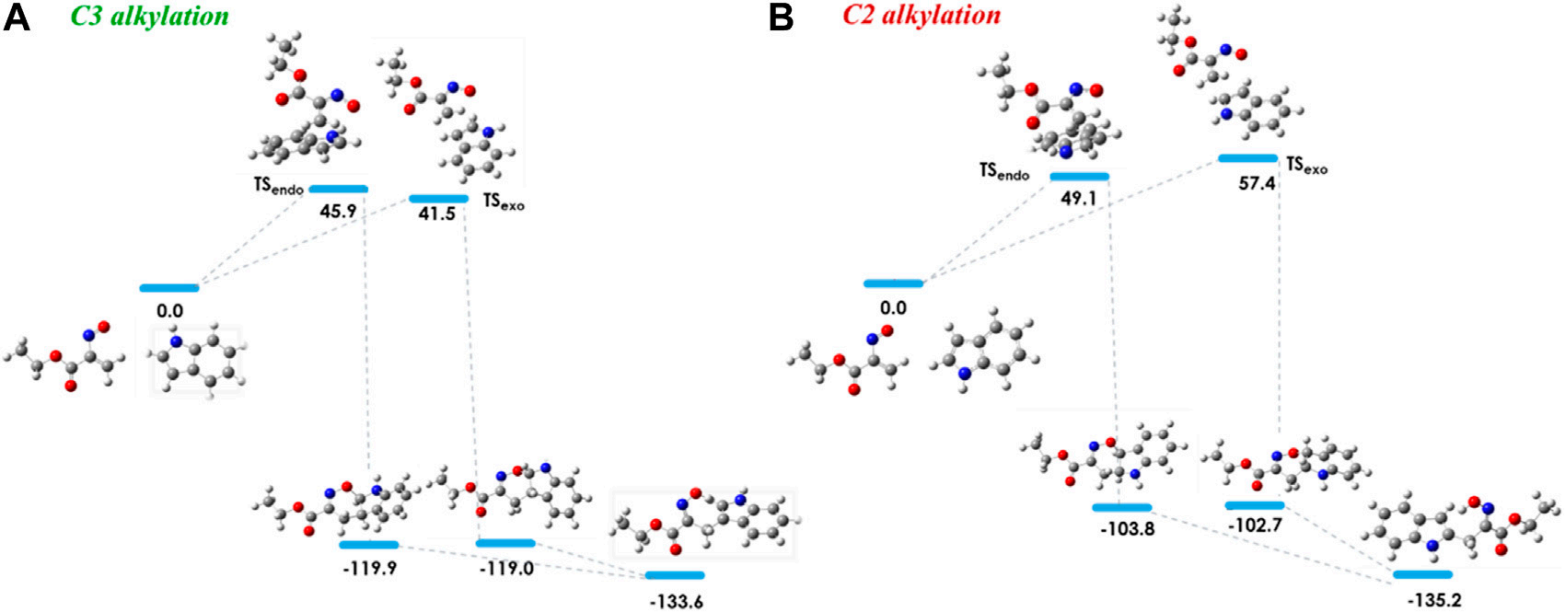
Relative stabilities (ΔE in kJ/mol) of transition states involved in the hetero-Diels-Alder reaction of ethyl nitrosoacrylate (**2a**) with indole considering the two regioisomers and both *endo* and *exo* approaches, both for C3 alkylation **(A)** and C2 alkylation **(B)**. All structures were optimized at the B3LYP/6-31G (d,p) level of theory. Color code: grey, carbon; red, oxygen; blue, nitrogen and white, hydrogen.

**FIGURE 4:**
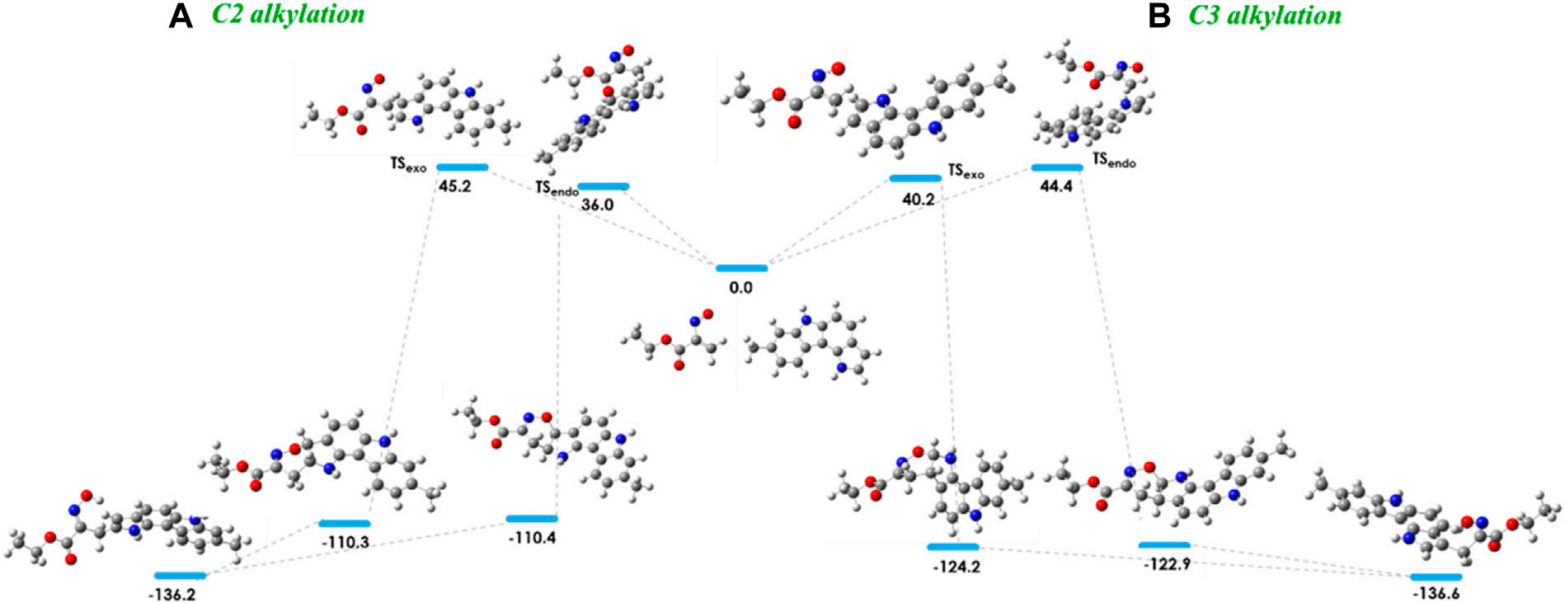
Relative stabilities (ΔE in kJ/mol) of transition states involved in the hetero-Diels-Alder reaction of ethyl nitrosoacrylate (**2a**) with 8-methyl-1,6-dihydropyrrolo [3,2-*c*]carbazole considering the C2 alkylation **(A)** and C3 alkylation **(B)** and both *endo* and *exo* approaches, obtained at the B3LYP/6-31G (d,p) level of theory. Color code: gray, carbon; red, oxygen; blue, nitrogen and white, hydrogen.

In order to investigate the observed and diverse regioselectivity, calculations at the DFT level of theory using the B3LYP hybrid functional (Becke, 1988; Lee et al., 1988; Becke, 1993) and the standard 6-31G (d,p) basis set were carried out for the hetero-Diels-Alder reaction of ethyl nitrosoacrylate (**2a**) with pyrrole, indole and 8-methyl-1,6-dihydropyrrolo[3,2-*c*]carbazole (**10**). For each heterocycle, relative stabilities of the different transition states (TS) involved in the hetero-Diels-Alder reactions were calculated, considering the two possible regioisomers and both *endo* and *exo* approaches (Figures 2–4). The individual contributions to the energy barriers associated with all the transition states studied for the reactions of ethyl nitrosoacrylate (**2a**) with pyrrole, indole and 8-methyl-1,6-dihydropyrrolo[3,2-*c*]carbazole (**10**) are reported in Tables 1–3, considering both zero-point-energy (ZPE) correction, and basis set superposition error (BSSE) correction.

**TABLE 1:** Electronic energy, E, zero-point vibration energy, ZPE, basis set superposition error correction, BSSE, and energy relative to the reactants, ∆E, of the transition states and final products identified for the reaction between 2a and pyrrole for both C2 and C3 alkylation. All values are obtained at the B3LYP/6-31G (d,p) level. ∆E includes both ZPE and BSSE corrections.

Structure	E/E_h_	ZPE/E_h_	BSSE/E_h_	∆E (kJ/mol)	
**2a**	−474.8238068	0.120430			
Pyrrole	−210.0437146	0.082615			
*C2 alkylation*					
TS_*endo*_ [**2a** + pyrrole]	−684.8670778	0.206067	0.007573	29.0	
TS_*exo*_ [**2a** + pyrrole]	−684.8579991	0.205785	0.006065	48.1	
*Endo* cycloadduct **3**	−684.9047253	0.210244			−78.3
*Exo* cycloadduct **3**	−684.9047404	0.210125			−79.1
Open chain oxime **4**	−684.9270218	0.208651			−141.5
*C3 alkylation*					
TS_*endo*_ [**2a** + pyrrole]	−684.8575322	0.206441	0.007227	54.1	
TS_*exo*_ [**2a** + pyrrole]	−684.8565208	0.205866	0.006217	52.6	
*Endo* cycloadduct **3′**	−684.9101386	0.210123			−93.3
*Exo* cycloadduct **3′**	−684.910211	0.210233			−93.2
Open chain oxime **4′**	−684.9244416	0.208671			−134.7

**TABLE 2:** Electronic energy, E, zero-point vibrational energy, ZPE, basis set superposition error correction, BSSE, and energy relative to the reactants, ∆E, of the transition states and final products identified for the reaction between 2a and indole for both C3 and C2 alkylation. All values are obtained at the B3LYP/6-31G (d,p) level. ∆E includes both ZPE and BSSE corrections.

Structure	E/E_h_	ZPE/E_h_	BSSE/E_h_	∆E (kJ/mol)	
**2a**	−474.8238068	0.120430			
Indole	−363.6013009	0.129796			
*C3 alkylation*					
TS_*endo*_ [**2a** + indole]	−838.4172558	0.253154	0.006689	45.9	
TS_*exo*_ [**2a** + indole]	−838.4179836	0.253118	0.005800	41.5	
*Endo* cycloadduct **7a**	−838.4774867	0.257339			−119.9
*Exo* cycloadduct **7a**	−838.4775886	0.256930			−119.0
Open chain oxime **8a**	−838.4812487	0.255474			−133.6
*C2 alkylation*					
TS_*endo*_ [**2a** + indole]	−838.41684	0.253104	0.007560	49.1	
TS_*exo*_ [**2a** + indole]	−838.4116629	0.252882	0.005752	57.4	
*Endo* cycloadduct **7a′**	−838.4717362	0.257327			−103.8
*Exo* cycloadduct **7a′**	−838.4709206	0.256950			−102.7
Open chain oxime **8a′**	−838.4817146	0.255325			−135.2

**TABLE 3:** Electronic energy, E, zero-point vibration energy, ZPE, basis set superposition error correction, BSSE, nand energy relative to the reactants, ∆E, of the transition states and final products identified for the reaction between 2a and pyrrolo [3,2-c]carbazole 10 for both C3 alkylation A) and C2 alkylation. All values are obtained at the B3LYP/6-31G (d,p) level. ∆E includes both ZPE and BSSE corrections.

Structure	E/E_h_	ZPE/E_h_	BSSE/E_h_	∆E (kJ/mol)	
**2a**	−474.8238068	0.120430			
**10**	−687.9494041	0.233090			
*C2 alkylation*					
TS_*endo*_ [**2a** + **10**]	−1162.771242	0.356763	0.008485	36.0	
TS_*exo*_ [**2a** + **10**]	−1162.764597	0.356287	0.005853	45.2	
*Endo* cycloadduct	−1162.8220909	0.361142			−110.4
*Exo* cycloadduct	−1162.822089	0.360943			−110.3
Open chain oxime **12a**	−1162.830221	0.358642			−136.2
*C3 alkylation*					
TS_*endo*_ [**2a** + **10**]	−1162.766076	0.356457	0.006851	44.4	
TS_*exo*_ [**2a** + **10**]	−1162.766980	0.356720	0.005897	40.2	
*Endo* cycloadduct	−1162.827975	0.361463			−122.9
*Exo* cycloadduct	−1162.827778	0.360788			−124.2
Open chain oxime **11a**	−1162.830837	0.359105			−136.6

In the reaction between nitrosoalkene **2a** and pyrrole, the computational results showed that the energy barrier associated with the formation of cycloadduct **3** by an *endo* approach is lower (about 25 kJ/mol) than the energy required for the formation of cycloadduct **3’**, which is in agreement with the regioselectivity observed experimentally (Figure 2). Furthermore, the open-chain oxime **4** was obtained as single product, which is more stable than the primarily formed bicyclic 1,2-oxazine **3**, as confirmed by DFT calculations (about 63 kJ/mol) (Figure 2; Table 1).

The 3-alkylated indole **8a** is obtained from the reaction of nitrosoalkene **2a** with indole via the hetero-Diels-Alder reaction by an *exo* approach. Indeed, this mechanistic pathway involves a lower energy transition state (ΔE = 41.5 kJ/mol) than that which would lead to the C2 alkylation product (Figure 3). Once again, the theoretical predictions of the regioselectivity are in agreement with the experimental results. In addition, the cycloadduct **7a**, involved in C3 alkylation pathway, is more stable (about 16 kJ/mol) than the homologue leading to C2 alkylation product (Figure 3; Table 2).

The energy barriers calculated for the transition states of the hetero-Diels-Alder reaction of nitrosoalkene **2a** with pyrrolo[3,2-*c*]carbazole **10** are very similar for the formation of both alkylated products (Figure 4). The DFT calculations showed that the formation of the 2-alkylated product proceeded via the *endo* transition state (TS_*endo*_), whereas the *exo* transition state (TS_*exo*_) was involved in the formation of the 3-alkylated product. The cycloadduct precursors of the 3-alkylated pyrrolo[3,2-*c*]carbazole **11a** are more stable than the precursors of the 2-alkylated pyrrolo [3,2-*c*]carbazole **12a** (about 13 kJ/mol), explaining the predominance of the 3-alkylated product. However, the stability of the open chain oximes **11a** and **12a** is very similar (Figure 4; Table 3). These computational results explain the isolation of both 2-alkylated and 3-alkylated products from the reaction between nitrosoalkene **2a** and pyrrolo[3,2-*c*]carbazole **10**.

**FIGURE 5:**
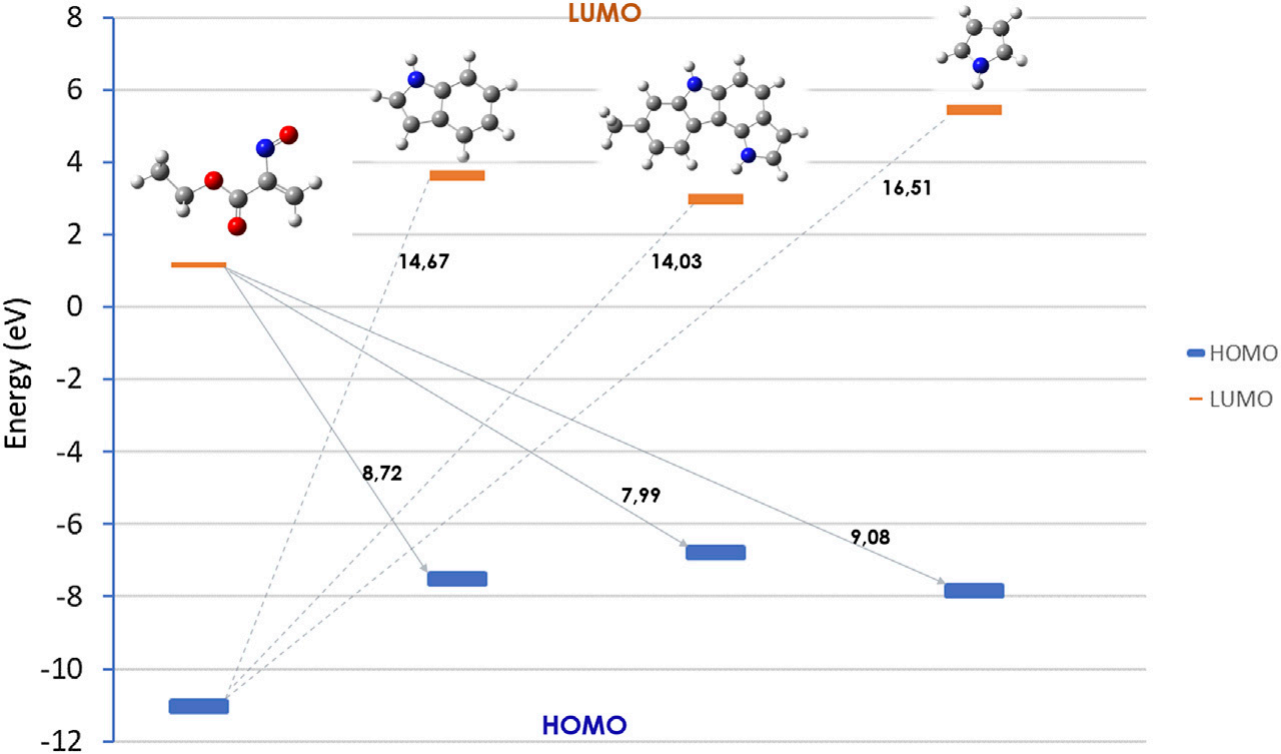
Relative energies (eV) of the HOMO and LUMO orbitals for nitrosoalkene **2a**, pyrrole, indole and pyrrolo [3,2-*c*]carbazole **10**, obtained at the HF/6-31G (d,p) level of theory.

Frontier Molecular Orbital (FMO) analysis of the hetero-Diels–Alder reaction of ethyl nitrosoacrylate (**2a**) with pyrrole, indole and pyrrolo[3,2-*c*]carbazole **10** was carried out. The relative energy values of the HOMO and LUMO orbitals of the reactants were obtained at the HF/6-31G (d,p) level of theory (Figure 5). The results show that the energy difference between the LUMO of the nitrosoalkene and the HOMO of the heterocycles is smaller (between 7.99 and 9.08 eV) than that calculated for the HOMO_nitrosoalkene_-LUMO_heterocycle_ pair (between 14.03 and 16.51 eV). Thus, the results show that these reactions are LUMO_nitrosoalkene_-HOMO_heterocycle_ controlled and confirm that pyrrole, indole and 8-methyl-1,6-dihydropyrrolo [3,2-*c*]carbazole participate in *inverse electron-demand* hetero-Diels-Alder reaction with nitrosoalkene **2a** acting as electron-rich 2π component. Furthermore, FMO analysis indicates that the molecular orbital energy profile of 8-methyl-1,6-dihydropyrrolo[3,2-c]carbazole is closer to the one of indole than to pyrrole.

**FIGURE 6:**
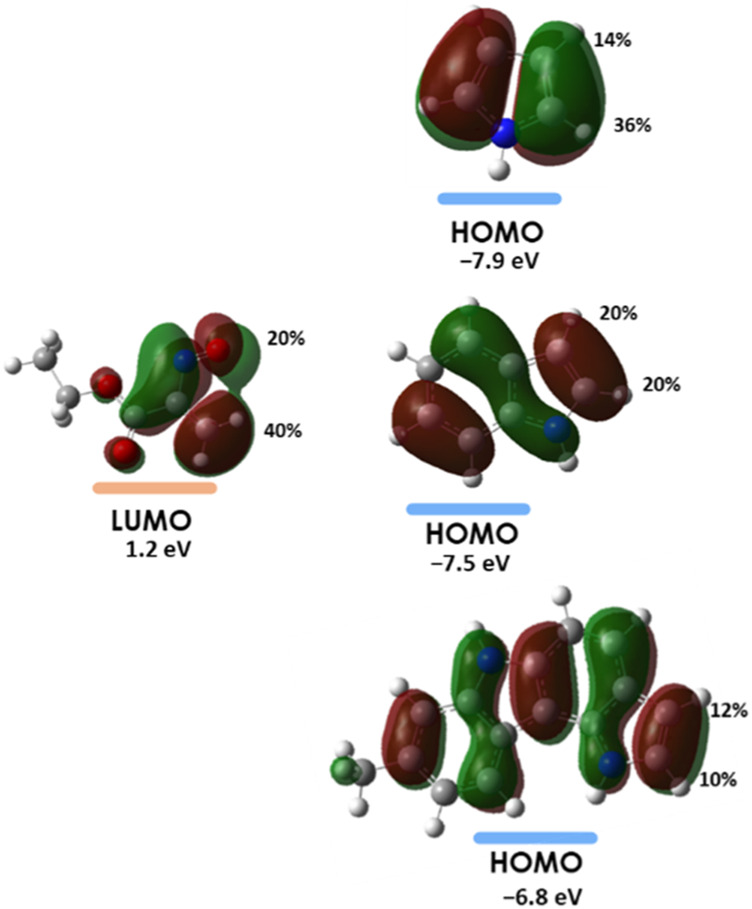
Orbital interaction diagrams for the hetero-Diels-Alder reactions with the indication of the orbital coefficients for the interacting orbitals obtained through NBO analysis at the HF/6-31G (d,p) level of theory.

The results of the Frontier Molecular Orbital interactions are in accordance with the observed regioselectivity, pointing generally to the formation of the product that stems from the interaction sites corresponding to the larger orbital coefficients. For indole the calculated orbital coefficients do not allow to distinguish between the two possible regiochemistries (Figure 6).

**SCHEME 4:**
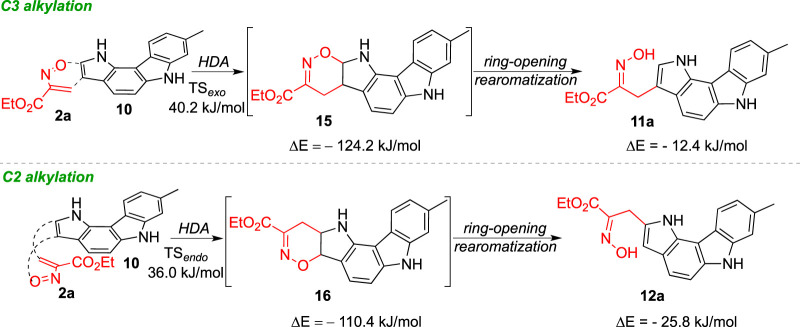
Mechanistic pathways for the formation of 3- and 2-alkylated pyrrolo[3,2-*c*]carbazoles.

Scheme 4 summarizes the mechanistic pathways leading to oxime-functionalized pyrrolo[3,2-c]carbazoles **11a** and **12a**. The 3-alkylated pyrrolo[3,2-c]carbazole is obtained via hetero-Diels-Alder reaction of nitrosoalkene **2a** with an *exo* approach followed by 1,2-oxazine ring-opening reaction. The synthesis of the 2-alkylated derivative takes place with the initial *endo* cycloaddition reaction and subsequent conversion into the final oxime **12a**. The selectivity towards the 3-alkylated pyrrolo[3,2-c]carbazole **11a** is determined by the more exothermic formation of the hetero-Diels-Alder cycloadduct than that derived from the opposite regiochemistry. It should be noted that the chemical behaviour of 8-methyl-1,6-dihydropyrrolo[3,2-*c*]carbazole is closer to the reactivity observed for indole than to that of pyrrole.

## Conclusion

The reactivity of nitrosoalkenes towards 8-methyl-1,6-dihydropyrrolo[3,2-*c*]carbazole was studied leading to the synthesis of oxime-functionalized pyrrolo[3,2-*c*]carbazoles. The mechanistic pathway involves a hetero-Diels-Alder reaction leading to the construction of a 1,2-oxazine ring which undergoes a ring-opening reaction to give open-chain oximes. Calculations at the DFT level of theory were carried out to investigate the regioselectivity of the hetero-Diels-Alder reaction of ethyl nitrosoacrylate with 8-methyl-1,6-dihydropyrrolo[3,2-*c*]carbazole as well as with pyrrole and indole, allowing a comparison between these three types of heterocycles. The computational results allowed the rationalization of the regioselectivity observed in the cycloaddition reaction and the formation of the more stable open chain oximes as the final products. The relative energy values of the Frontier HOMO and LUMO molecular orbitals for the ethyl nitrosoacrylate and the studied heterocyclic dienophiles were also calculated, substantiating that the cycloadditions are LUMO_heterodiene_-HOMO_dienophile_ controlled.

The construction of a new hexahydropyrido[4′,3':4,5]pyrrolo [3,2-*c*]carbazole system from one of the new 3-alkylated pyrrolo [3,2-*c*]carbazole was achieved in high yield via a one-pot two-step approach.

## Experimental

*General Information*: NMR spectra were recorded on a Bruker Avance III instrument operating at 400 MHz (^1^H) or 100 MHz (^13^C). Chemical shifts are expressed in ppm relative to TMS and coupling constants (*J*) are in Hz. Infrared spectra (IR) were recorded in a Fourier Transform spectrometer. High-resolution mass spectra (HRMS) were obtained on a TOF VG Autospec M spectrometer with electrospray ionization (ESI). Melting points were recorded in open glass capillaries. Thin Layer Chromatography (TLC) was performed using precoated silica gel plates. Flash chromatography was performed using silica gel 60 as a stationary phase. Ethyl bromopyruvate oxime (**1a**) (Gilchrist and Roberts, 1983), 2-bromo-1-phenylethanone oxime (**1b**) (Masaki et al., 1967) and 8-methyl-1,6-dihydropyrrolo [3,2-*c*]carbazole (**10**) (Benzi et al., 2019) were prepared as described in the literature.

## General procedure for the hetero-Diels-Alder reactions

Sodium carbonate (0.75 mmol) was added to a solution of *a*-bromooxime **1** (0.15 mmol) and 8-methylpyrrolo[3,2-*c*]carbazole **10** (0.22 mmol) in dry dichloromethane (10 mL). The reaction mixture was stirred at room temperature for the time indicated in each case, monitored by TLC. Upon completion, the mixture was filtered through a Celite pad, which was washed with ethyl acetate (2 × 10 mL). The solvent was evaporated, and the products were purified by flash chromatography.

**(*E*)-3-(2-Ethoxycarbonyl-2-hydroxyiminoethyl)-8-methyl-1,6-dihydropyrrolo[3,2-*c*]carbazole** (**11a**) and **(*E*)-2-(2-Ethoxycarbonyl-2-hydroxyiminoethyl)-8-methyl-1,6-dihydropyrrolo[3,2-*c*]carbazole** (**12a**). Obtained from oxime **1a** (31.5 mg, 0.15 mmol) and 8-methylpyrrolo[3,2-*c*]carbazole **10** (48.5 mg, 0.22 mmol) as described in general procedure (reaction time: 18 h). Purification of the crude product by flash chromatography [ethyl acetate/hexane (1:1)], gave, in order of elution, **12a** obtained as a beige solid (11.0 mg, 21%) and **11a** obtained as a beige solid (22.0 mg, 42%).

Data for compound **11a**: mp 199.7°C-201.6°C (from ethyl acetate/hexane). IR (KBr) ν 796, 1020, 1136, 1196, 1389, 1429, 1643, 1714, 3261, 3419 and 3446 cm^-1^. ^1^H NMR (Acetone-*d*_*6*_) δ: 1.21 (t, *J* = 7.2 Hz, 3H), 2.49 (s, 3H), 4.15 (d, *J* = 0.8 Hz, 2H), 4.17 (q, *J* = 7.2 Hz, 2H), 7.02 (dd, *J* = 8.0 and 0.8 Hz, 1H), 7.14 (dd, *J* = 2.0 and 0.8 Hz, 1H), 7.25 (d, *J* = 8.4 Hz, 1H), 7.33 (s, 1H), 7.74 (d, *J* = 8.8 Hz, 1H), 8.24 (d, *J* = 8.0 Hz, 1H), 10.22 (br s, 1H), 10.50 (br s, 1H), 11.35 (s, 1H). ^13^C NMR (Acetone-*d*_*6*_) δ: 14.4, 21.1, 22.0, 61.5, 105.0, 108.3, 111.4, 111.6, 118.0, 120.5, 121.0, 121.3, 121.5, 121.8, 130.9, 134.2, 138.1, 140.3, 152.5, 164.9. HRMS (ESI): calcd. for C_20_H_18_N_3_O_3_, 348.1354 [M-H^+^]; found, 348.1352.

Data for compound **12a**: mp 154.8°C-156.0°C (from carbon tetrachloride). IR (KBr) ν 804, 1020, 1132, 1261, 1382, 1464, 1614, 1722, 2924, 2977 and 3381 cm^-1^. ^1^H NMR (Acetone-*d*_*6*_) δ: 1.26 (t, *J* = 7.2 Hz, 3H), 2.49 (s, 3H), 4.22 (d, *J* = 1.2 Hz, 2H), 4.25 (q, *J* = 7.2 Hz, 2H), 6.32 (dd, *J* = 2.4 and 1.2 Hz, 1H), 7.03 (dd, *J* = 7.6 and 1.2 Hz, 1H), 7.19 (d, *J* = 8.4 Hz, 1H), 7.33 (s, 1H), 7.46 (d, *J* = 8.8 Hz, 1H), 8.19 (d, *J* = 8.0 Hz, 1H), 10.18 (br s, 1H), 10.26 (br s, 1H), 11.58 (br s, 1H). ^13^C NMR (Acetone-*d*_*6*_) δ: 14.4, 22.0, 24.3, 61.9, 102.0, 105.2, 111.6, 118.8, 120.4, 120.9, 121.2, 122.8, 124.4, 130.6, 131.3, 134.3, 137.8, 140.3, 150.7, 164.9. HRMS (ESI): calcd. for C_20_H_20_N_3_O_3_, 350.1499 [M + H^+^]; found, 350.1496.

**(*E*)-3-(2-Phenyl-2-hydroxyiminoethyl)-8-methyl-1,6-dihydropyrrolo[3,2-*c*]carbazole** (**11b**) and **(*E*)-2-(2-phenyl-2-hydroxyiminoethyl)-8-methyl-1,6-dihydropyrrolo[3,2-*c*]carbazole** (**12b**). Obtained from oxime **1b** (32.1 mg, 0.15 mmol) and 8-methylpyrrolo[3,2-*c*]carbazole **10** (48.5 mg, 0.22 mmol) as described in general procedure (reaction time: 48 h). Purification of the crude product by flash chromatography [ethyl acetate/hexane, (1:2)], gave, in order of elution, **12b** obtained as a beige solid (4.2 mg, 8%) and **11b** obtained as a beige solid (19.6 mg, 37%).

Data for compound **11b**: mp 179.8°C-181.7°C (from ethyl acetate/hexane). IR (KBr) ν 687, 761, 943, 1124, 1298, 1331, 1389, 1429, 1462, 1639, 2918, 3059 and 3392 cm^-1^. ^1^H NMR (Acetone-*d*_*6*_) δ: 2.48 (s, 3H), 4.39 (d, *J* = 1.2 Hz, 2H), 7.00 (dd, *J* = 8.0 and 1.2 Hz, 1H), 7.05 (dd, *J* = 2.4 and 1.2 Hz, 1H), 7.24-7.29 (m, 4H), 7.32 (br s, 1H), 7.75-7.77 (m, 3H), 8.21 (d, *J* = 8.0 Hz, 1H), 10.22 (br s, 1H), 10.41 (br s, 1H), 10.51 (s, 1H). ^13^C NMR (Acetone-*d*_*6*_) δ: 22.0, 22.1, 105.0, 109.6, 111.5, 112.4, 117.8, 120.5, 120.9, 121.1, 121.3, 121.8, 127.3, 128.9, 129.2, 131.0, 134.2, 137.7, 138.2, 140.3, 157.2. HRMS (ESI): calcd. for C_23_H_20_NO_3_, 354.1601 [M + H^+^]; found, 354.1598.

Data for compound **12b**: mp 118.0°C-119.5°C (from carbon tetrachloride). IR (KBr) ν 694, 760, 800, 1184, 1288, 1385, 1620, 1697, 2850, 2920 and 3398 cm^-1^. ^1^H NMR (Acetone-*d*_*6*_) δ: 2.49 (s, 3H), 4.45 (d, *J* = 0.8 Hz, 2H), 6.33 (dd, *J* = 2.0 and 0.8 Hz, 1H), 7.03 (dd, *J* = 8.0 and 1.6 Hz, 1H), 7.17 (d, *J* = 8.4 Hz, 1H), 7.31-7.38 (m, 4H), 7.42 (d, *J* = 8.4 Hz, 1H), 7.57-7.61 (m, 1H), 7.82-7.85 (m, 1H), 8.15 (d, *J* = 8.0 Hz, 1H), 10.18 (br s, 1H), 10.33 (br s, 1H), 10.82 (s, 1H). ^13^C NMR (Acetone-*d*_*6*_) δ: 21.1, 24.6, 101.2, 104.2, 107.1, 110.7, 117.8, 120.0, 120.2, 124.9, 126.2, 127.3, 128.2, 128.5, 128.8, 129.8, 131.6, 133.4, 136.3, 136.9, 139.4, 154.9. HRMS (ESI): calcd. for C_23_H_20_NO_3_, 354.1601 [M + H^+^]; found, 354.1598.

**Ethyl 1,1,9-trimethyl-1,2,3,4,7,12-hexahydropyrido[4′,3':4,5]pyrrolo[3,2-*c*]carbazole-3-carboxylate** (**14**): Zinc powder (80 mg, 1.22 mmol) was added portion-wise to a solution of 3-alkylated pyrrolo [3,2-*c*]carbazole **11a** (35.6 mg; 0.102 mmol) in acetic acid (1.5 mL) and acetone (0.1 mL). The resulting mixture was stirred at room temperature for 24 h. After this time, zinc powder (80 mg, 1.22 mmol) was added and the resulting mixture stirred at room temperature for more 24 h. Upon completion, the zinc salts were removed by filtration through a Celite pad, which was washed with ethyl acetate (3 × 10 mL). The filtrate was neutralized with aqueous NaOH 5% to pH 7 and then extracted with ethyl acetate (3 × 20 mL). The organic extracts were dried over Na_2_SO_4_ and the solvent evaporated off. Compound **14** was purified by flash chromatography [ethyl acetate/hexane, (1:1)] and obtained as a beige solid (32.5 mg, 85%). mp 114.0°C-115.8°C (from ethyl acetate/hexane). IR (KBr) ν 808, 1030, 1192, 1265, 1469, 1620, 1705, 2921, 2962 and 3392 cm^-1^. ^1^H NMR (CDCl_3_) δ: 1.37 (t, *J* = 7.2 Hz, 3H), 1.58 (s, 3H), 1.66 (s, 3H), 2.55 (s, 3H), 2.84 (dd, *J* = 15.2 and 11.2 Hz, 1H), 3.20 (dd, *J* = 15.2 and 4.2 Hz, 1H), 4.00 (dd, *J* = 11.2 and 4.2 Hz, 1H), 4.28-4.36 (m, 2H), 7.13 (dd, *J* = 8.0 and 1.6 Hz, 1H), 7.22 (d, *J* = 8.4 Hz, 1H), 7.26 (s, 1H), 7.49 (d, *J* = 8.8 Hz, 1H), 7.95 (d, *J* = 8.0 Hz, 1H), 8.10 (s, 1H), 8.11 (s, 1H). ^13^C NMR (CDCl_3_) δ: 14.4, 22.2, 27.0, 28.9, 30.1, 51.1, 53.1, 61.3, 104.4, 107.4, 107.8, 111.0, 116.5, 119.6, 120.3, 120.7, 120.9, 129.9, 134.6, 137.0, 137.3, 139.2, 174.0. HRMS (ESI): calcd. for C_23_H_26_N_2_O_3_, 376.2019 [M^+^]; found, 376.2015.

**Crystallographic data for Ethyl 1,1,9-trimethyl-1,2,3,4,7,12-hexahydropyrido[4′,3':4,5]pyrrolo[3,2-*c*]carbazole-3-carboxylate** (**14**): A single crystal of compound **14** ^.^ H_2_O^.^C_2_H_5_OH was submitted to X-ray data collection on a Bruker APEX-II CCD diffractometer with a graphite monochromated Cu-Kα radiation (*λ* = 1.54178 Å) at 100 K. The structure was solved by direct methods implemented in SHELXS-97 program (Sheldrick, 2008). The refinement was carried out by full-matrix anisotropic least-squares on F^2^ for all reflections for non-H atoms by means of the SHELXL (version 2019/2) program (Sheldrick, 2015). The structure crystallizes with a water and an ethanol molecule in the Triclinic crystal system, space group P-1 with one molecule in the asymmetric unit. Crystallographic data for this structure has been deposited with the Cambridge Crystallographic Data Centre as supplementary publication no. CCDC 2222762. Copies of the data can be obtained, free of charge, on application to CCDC, 12 Union Road, Cambridge CB2 1EZ, UK; (fax: +44 0) 1223 336 033; or e-mail: deposit@ccdc.cam.ac.uk).

## Computational methodology

Calculations were performed with Gaussian 09 (Frisch et al., 2009) and Gamess (Schmidt et al., 1993) program packages. All structures were fully optimized at the DFT level of theory, using the B3LYP hybrid functional (Becke, 1988; Lee et al., 1988; Becke, 1993) and the standard 6-31G (d,p) basis set.

Vibrational frequencies were calculated at the same level of theory to evaluate the zero-point vibrational energy, ZPE, and to confirm the nature of the stationary points, that in the case of the transition states were characterized by having only one imaginary frequency. Inspection of the corresponding imaginary frequency allowed to confirm that the transition states connect the reactants with the expected products. The geometrical counterpoise correction was added to all transition state structures. Frontier molecular orbitals were calculated on DFT-optimized structures at the HF level of theory with the 6-31G(d) basis set. The orbital coefficients were calculated using the NBO module of Gaussian. Graphical representations were obtained with Gaussview.

## Data Availability

The datasets presented in this study can be found in online repositories. The names of the repository/repositories and accession number(s) can be found below: https://www.ccdc.cam.ac.uk/, 2222762.
