# Supplementary material for: Reactivity of ethyl nitrosoacrylate toward pyrrole, indole and pyrrolo[3,2-c]carbazole: an experimental and theoretical study
Source: Front Chem. 2023 Aug 8;11:1229669. doi: 10.3389/fchem.2023.1229669 (PMC10443595; doi:10.3389/fchem.2023.1229669)
Supplement: Supplementary file 1 [file DataSheet1.docx]

**Reactivity of Ethyl Nitrosoacrylate toward Pyrrole, Indole and Pyrrolo[3,2-*c*]carbazole: An Experimental and Theoretical Study**

Alice Benzi,^2^ Susana M. M. Lopes,^1^ Sandra C. C. Nunes,^1^ Gianluca Giorgi,^3^ Lara Bianchi,^2^ Cinzia Tavani,^2^ Alberto A. C. C. Pais,^1^ Giovanni Petrillo,^2^ Teresa M. V. D. Pinho e Melo^1,*^

^1^University of Coimbra, Coimbra Chemistry Centre-Institute of Molecular Sciences (CQC-IMS), Department of Chemistry, 3004-535 Coimbra.

^2^Department of Chemistry and Industrial Chemistry, University of Genova, Via Dodecaneso 31, 16146 Genova, Italy.

^3^Department of Biotechnology, Chemistry and Pharmacy, University of Siena, Via A. Moro, I-53100 Siena, Italy

**Supplementary material**

**Table of contents**

1. Copies of NMR spectra of all new compounds ………………………..………. S2
2. X-Ray Crystallographic data for compound **14** ………………….…………... S16
3. Theoretical Calculations ………………………………………...…………... S17
4. **Copies of NMR spectra of all new compounds**


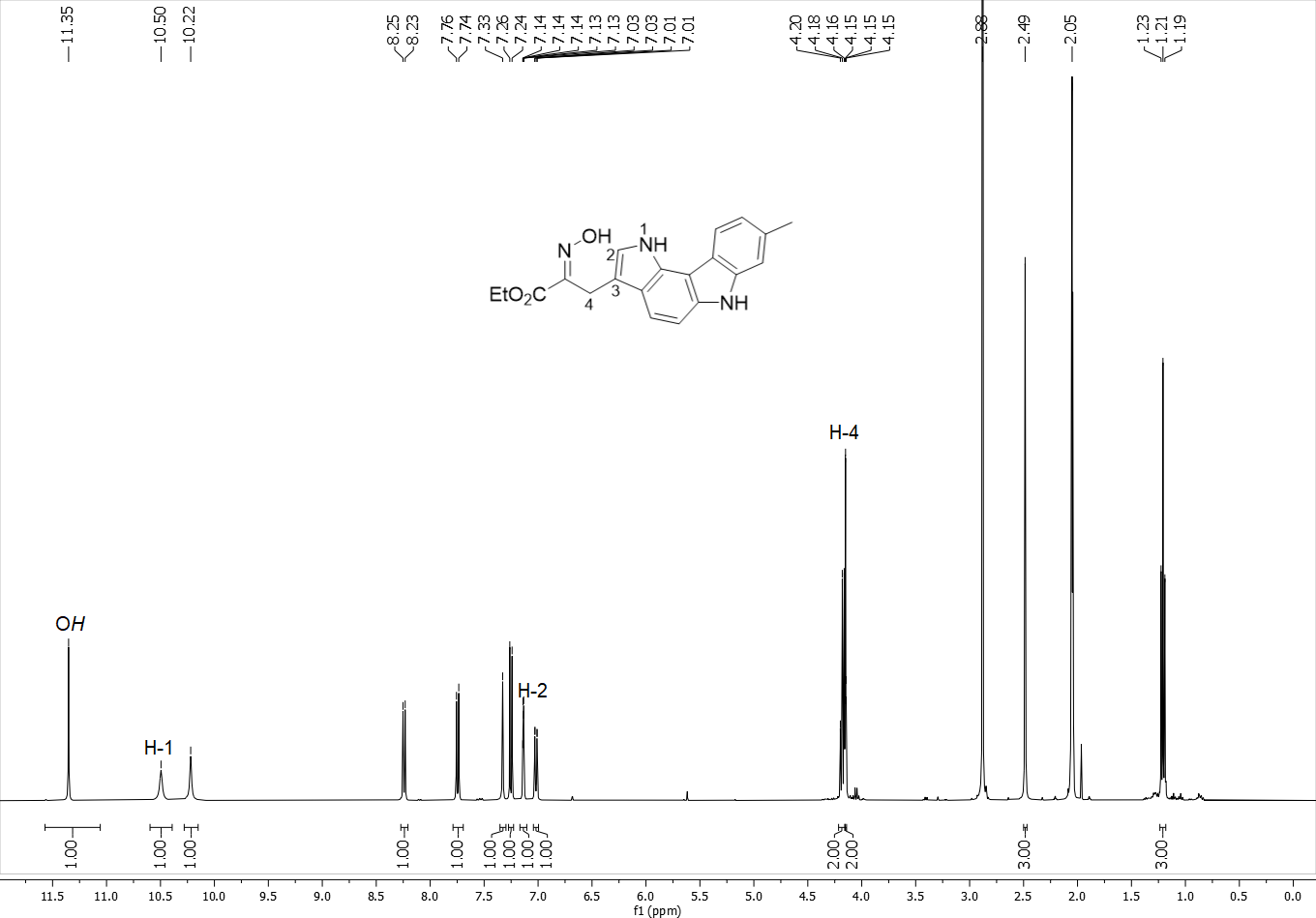


**Figure S1.** ^1^H NMR spectrum of compound **11a** (400 MHz, Acetone-*d_6_*).


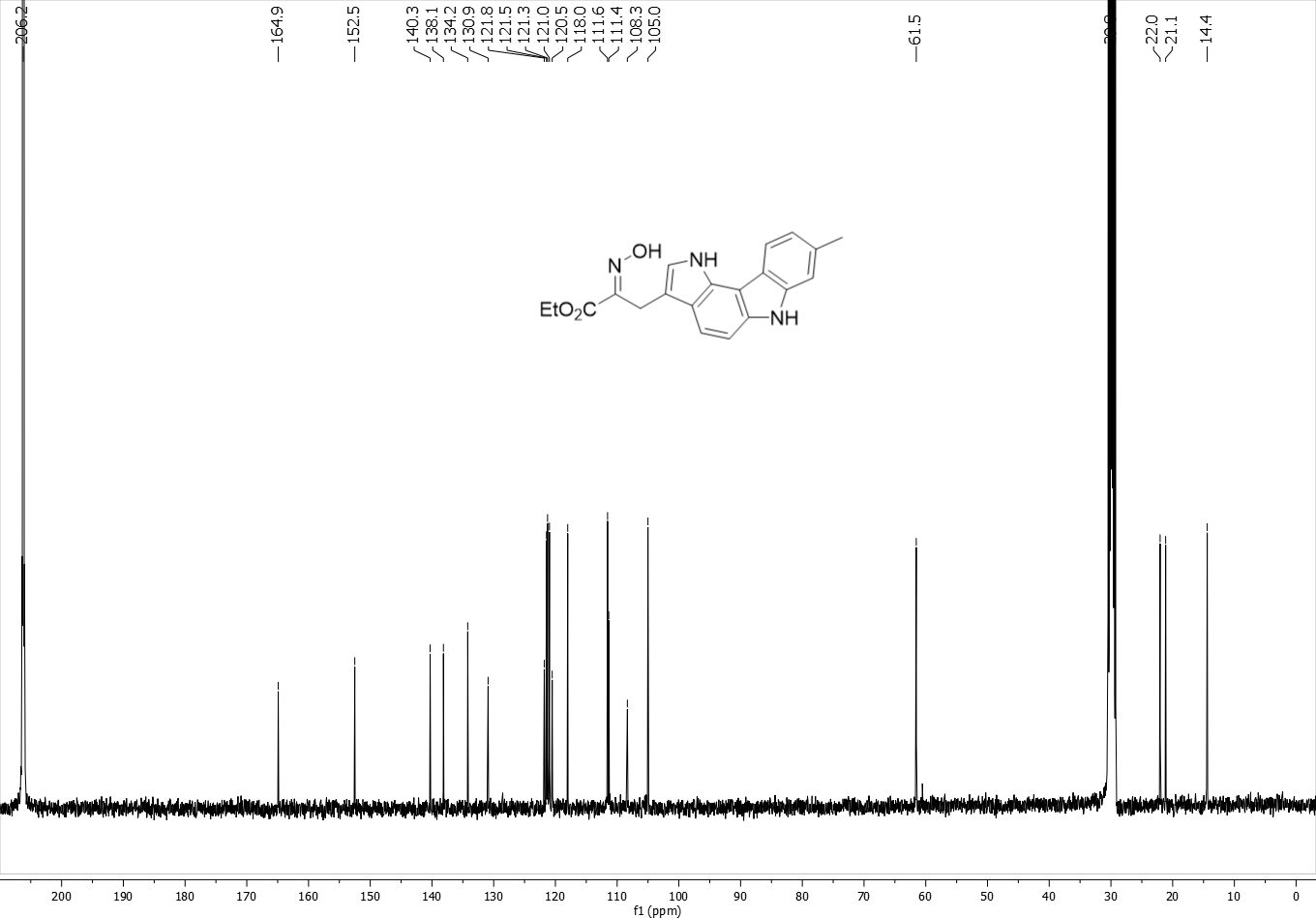


**Figure S2.** ^13^C NMR spectrum of compound **11a** (100 MHz, Acetone-*d_6_*).


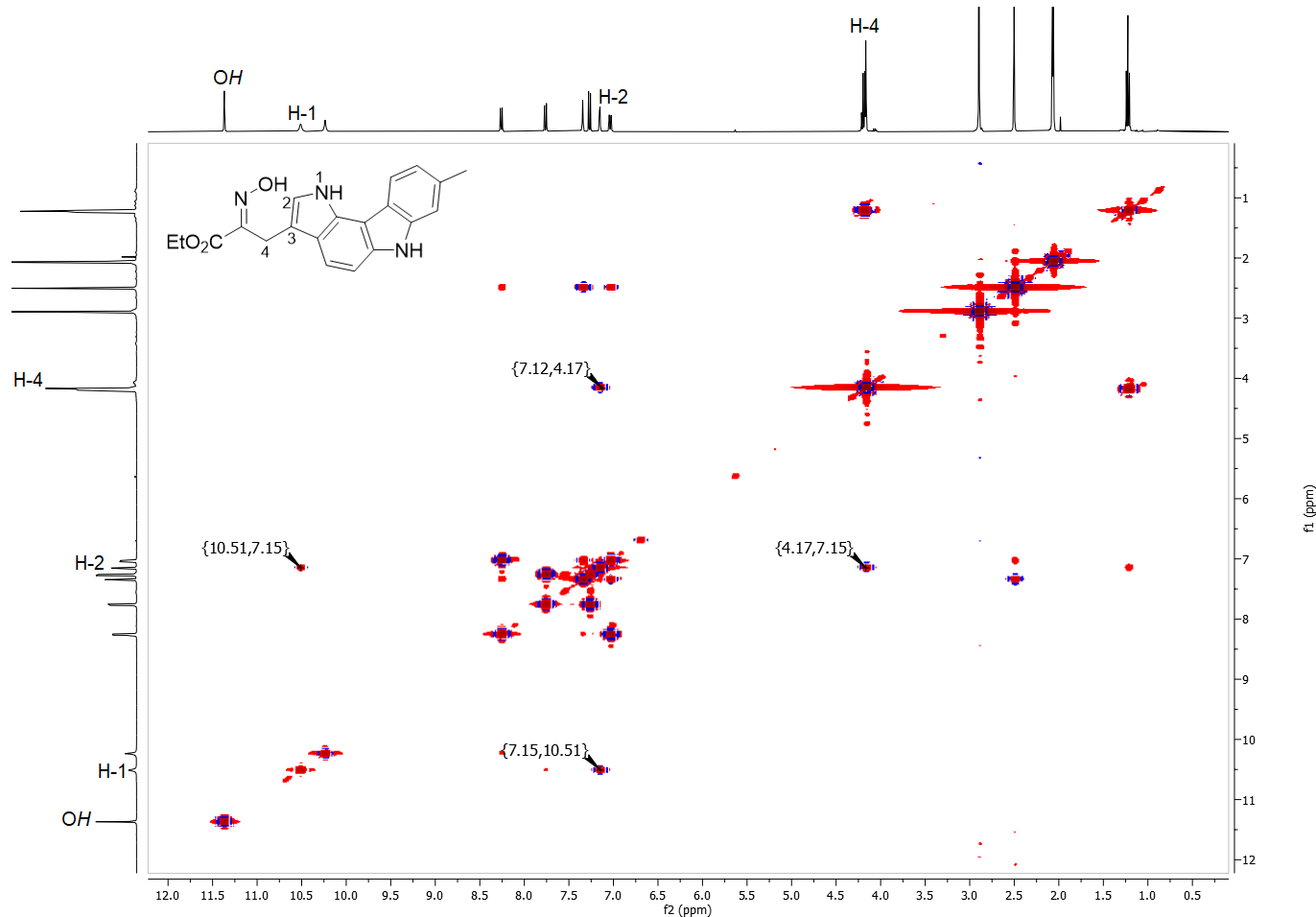


**Figure S3.** COSY spectrum of compound **11a** (400 MHz, Acetone-*d_6_*).


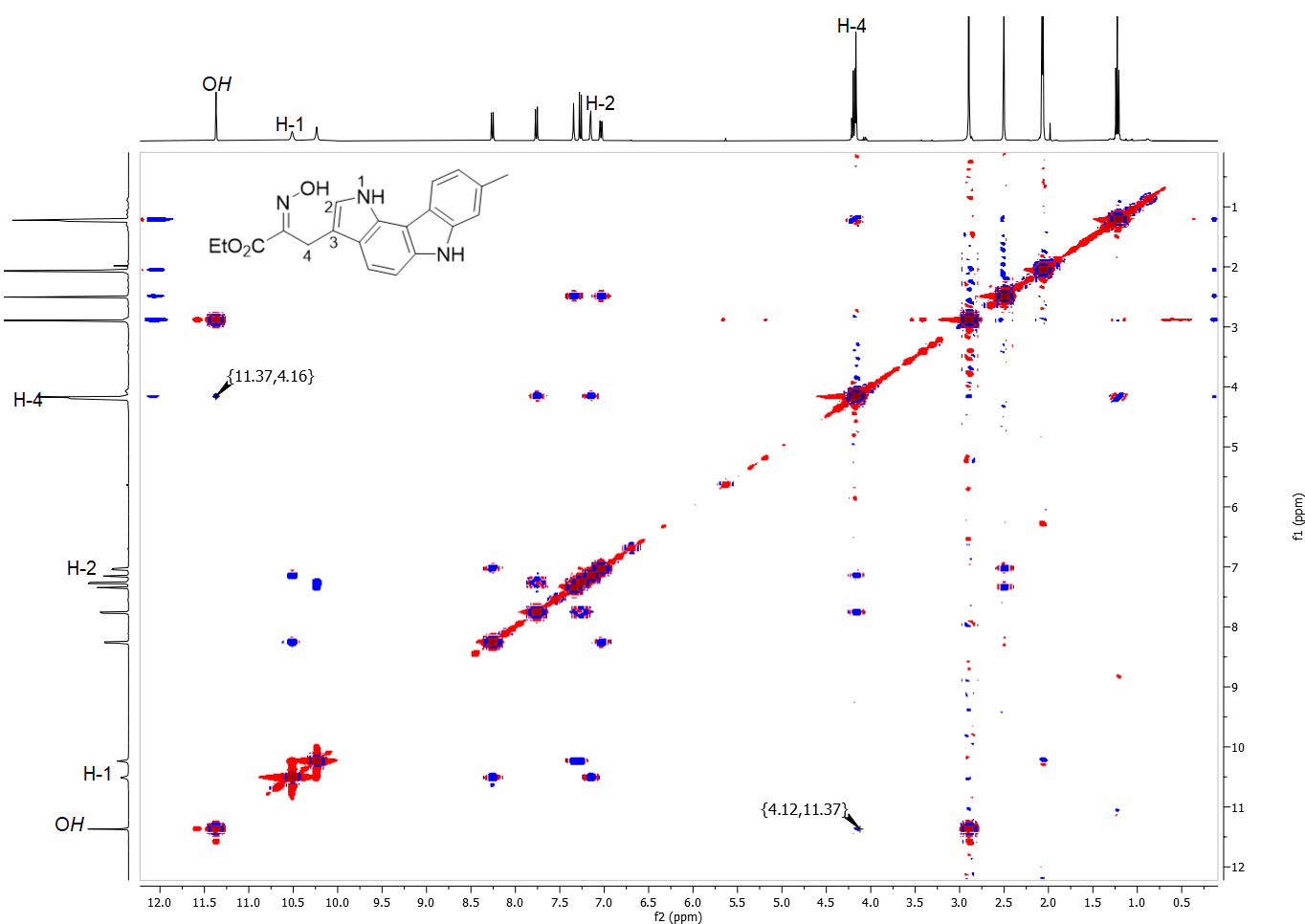


**Figure S4.** NOESY spectrum of compound **11a** (400 MHz, Acetone-*d_6_*).


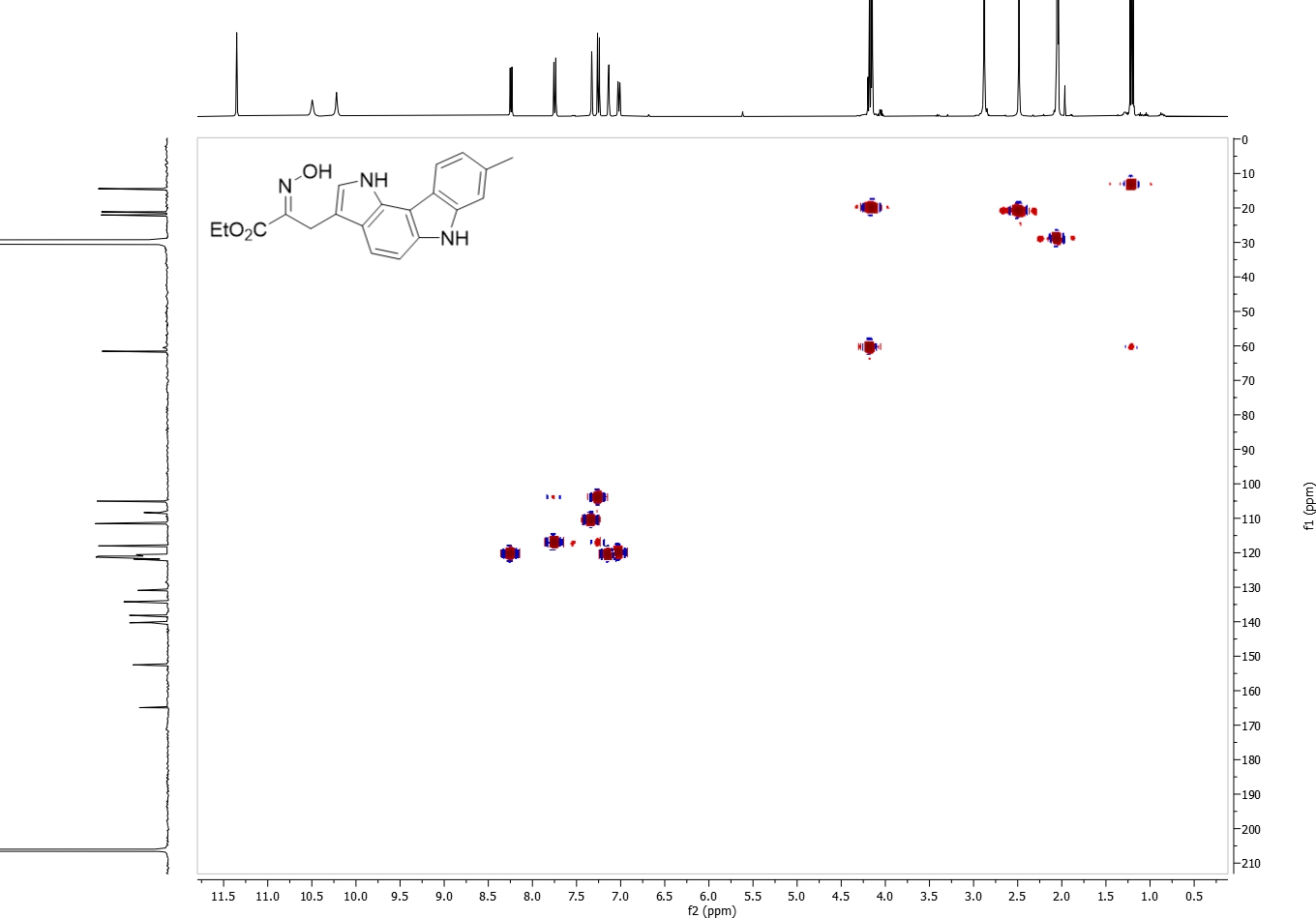


**Figure S5.** HSQC spectrum of compound **11a** (Acetone-*d_6_*).


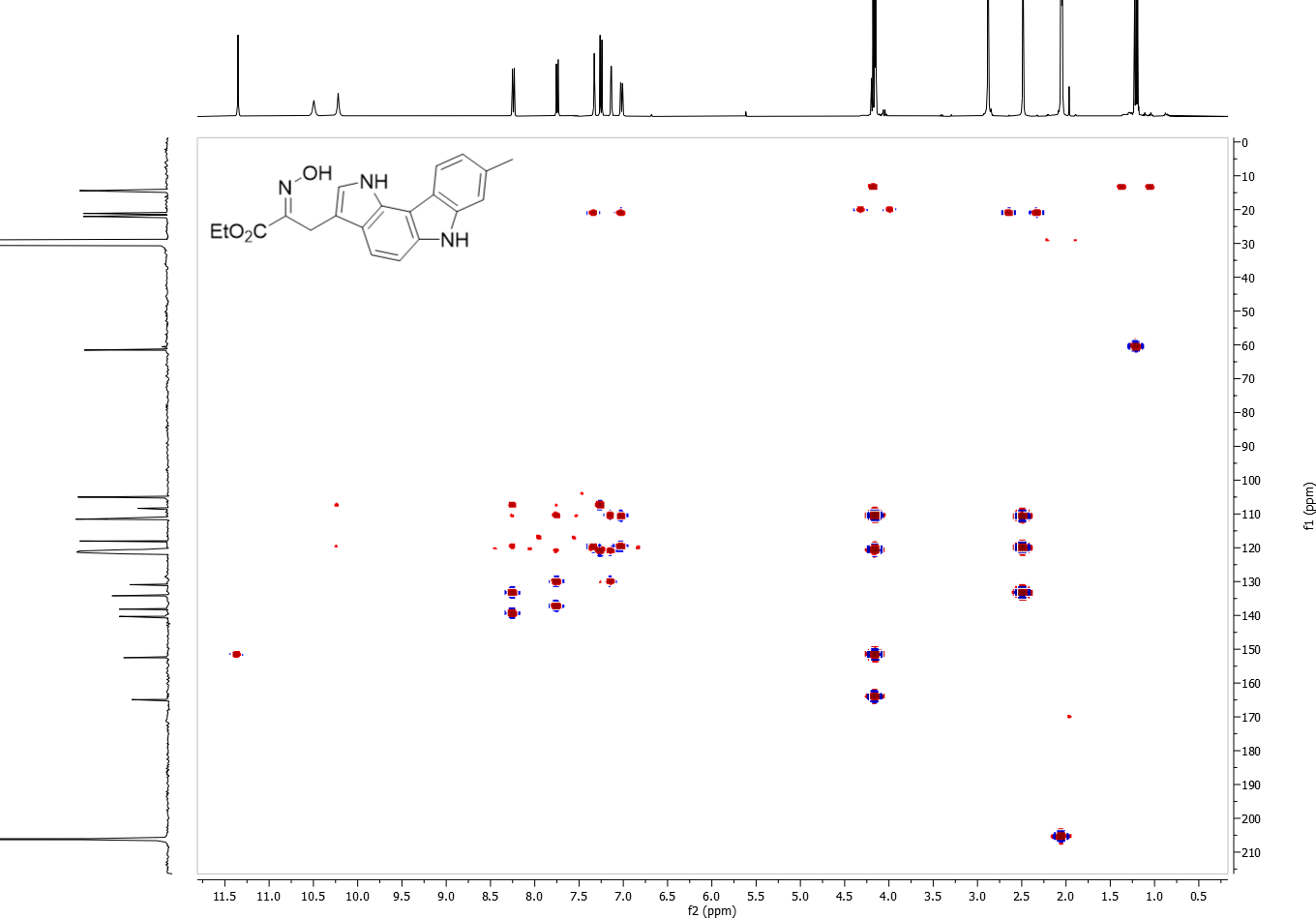


**Figure S6.** HMBC spectrum of compound **11a** (Acetone-*d_6_*).


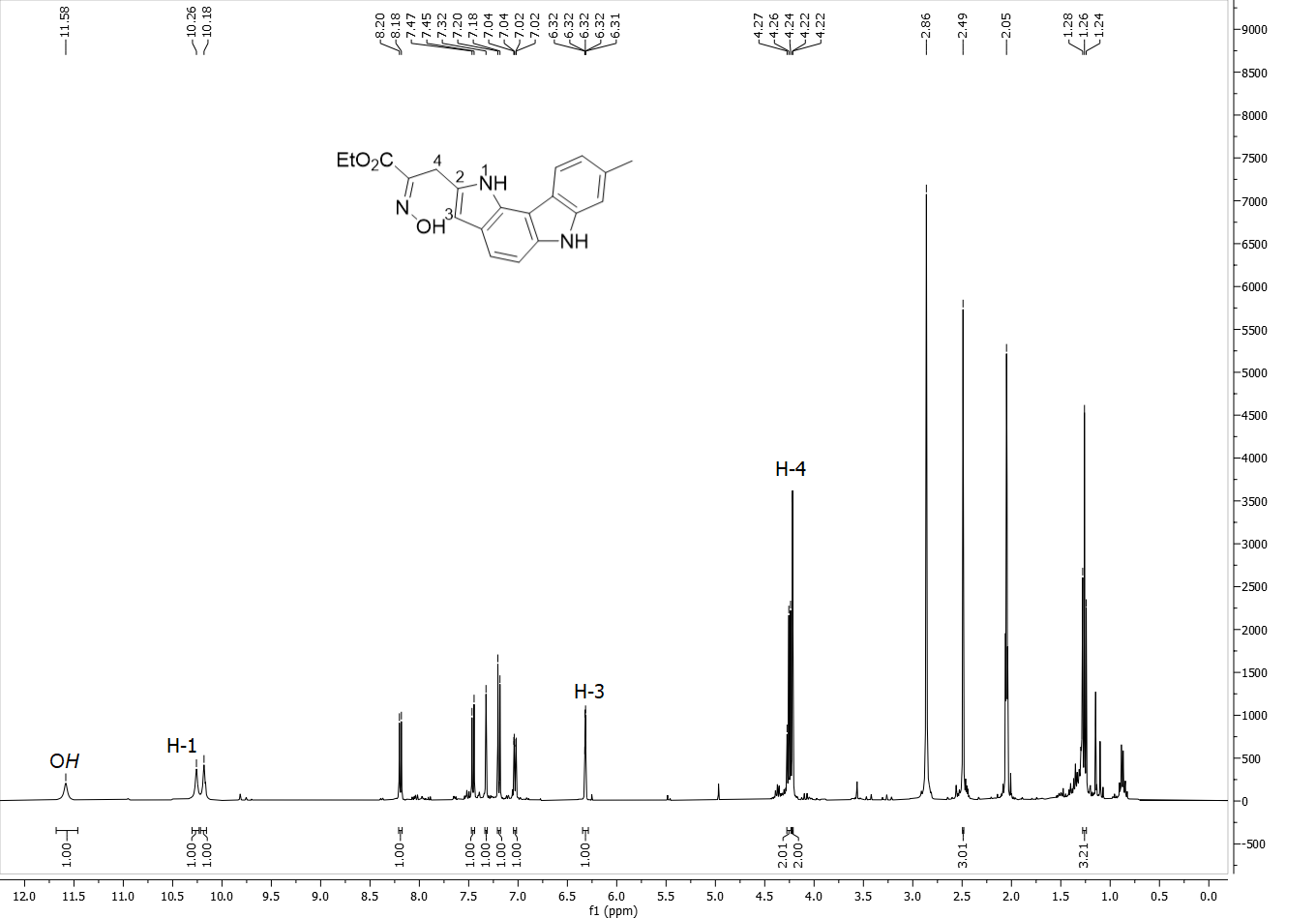


**Figure S7.** ^1^H NMR spectrum of compound **12a** (400 MHz, Acetone-*d_6_*).


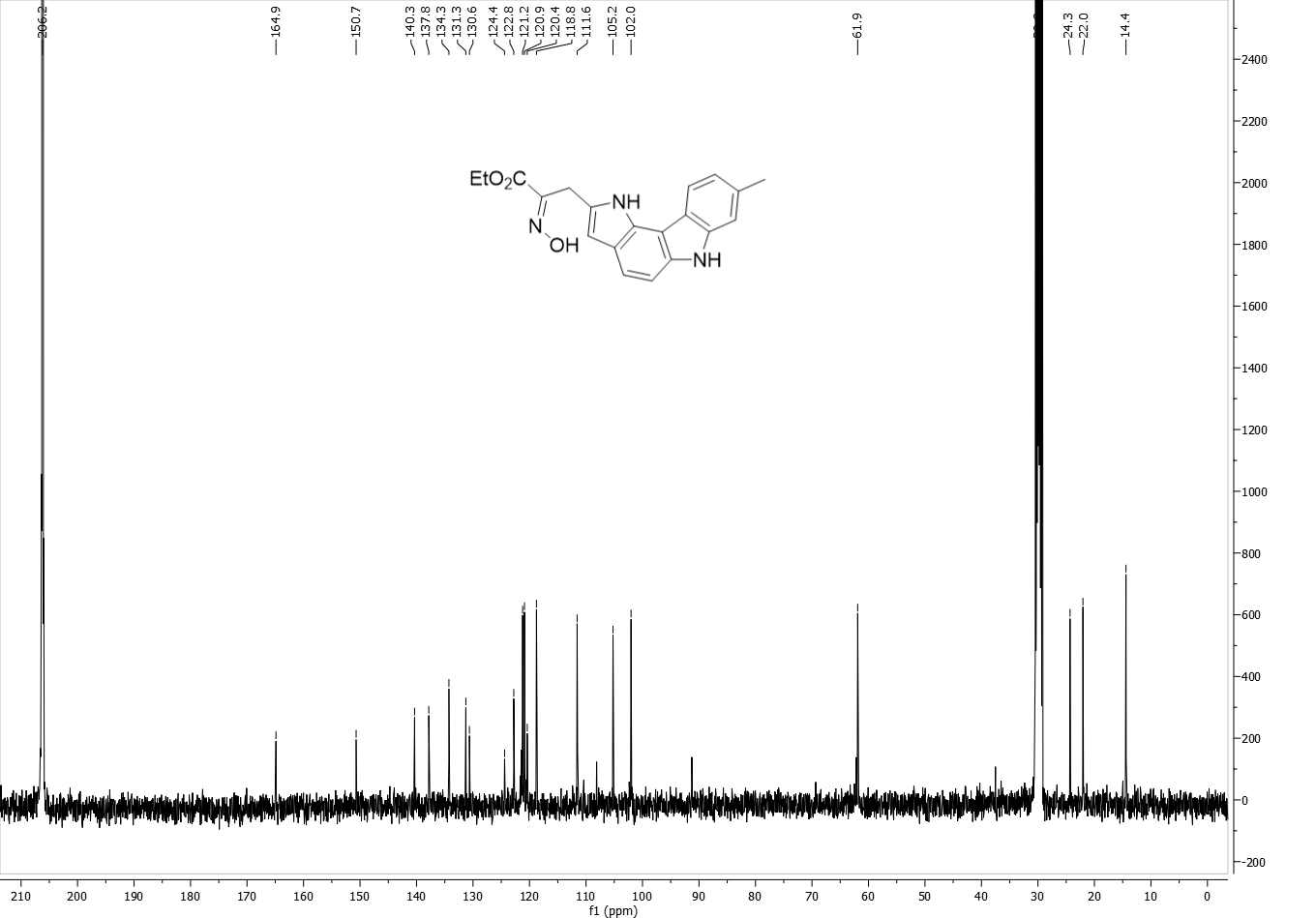


**Figure S8.** ^13^C NMR spectrum of compound **12a** (100 MHz, Acetone-*d_6_*).


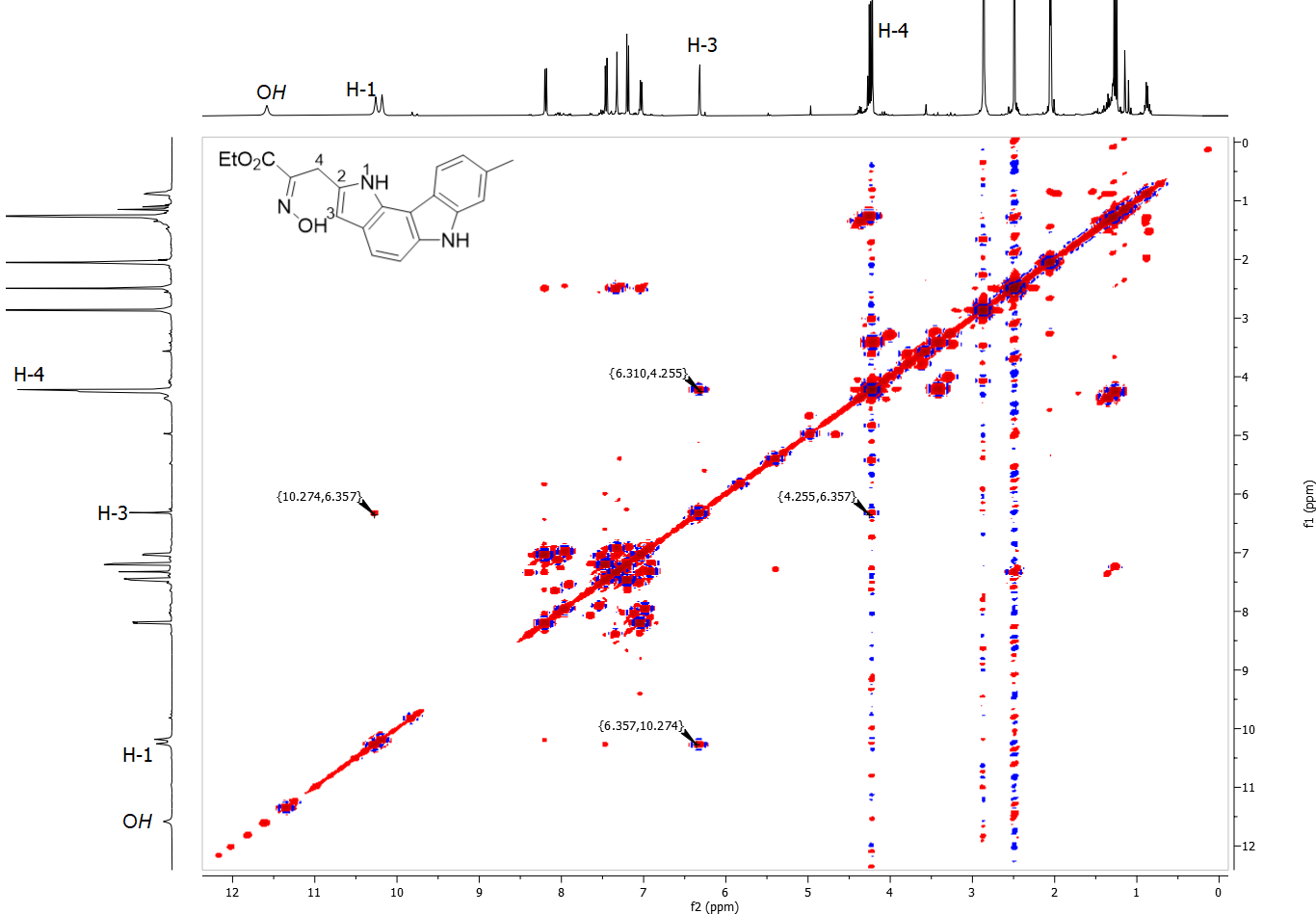


**Figure S9.** COSY spectrum of compound **12a** (400 MHz, Acetone-*d_6_*).


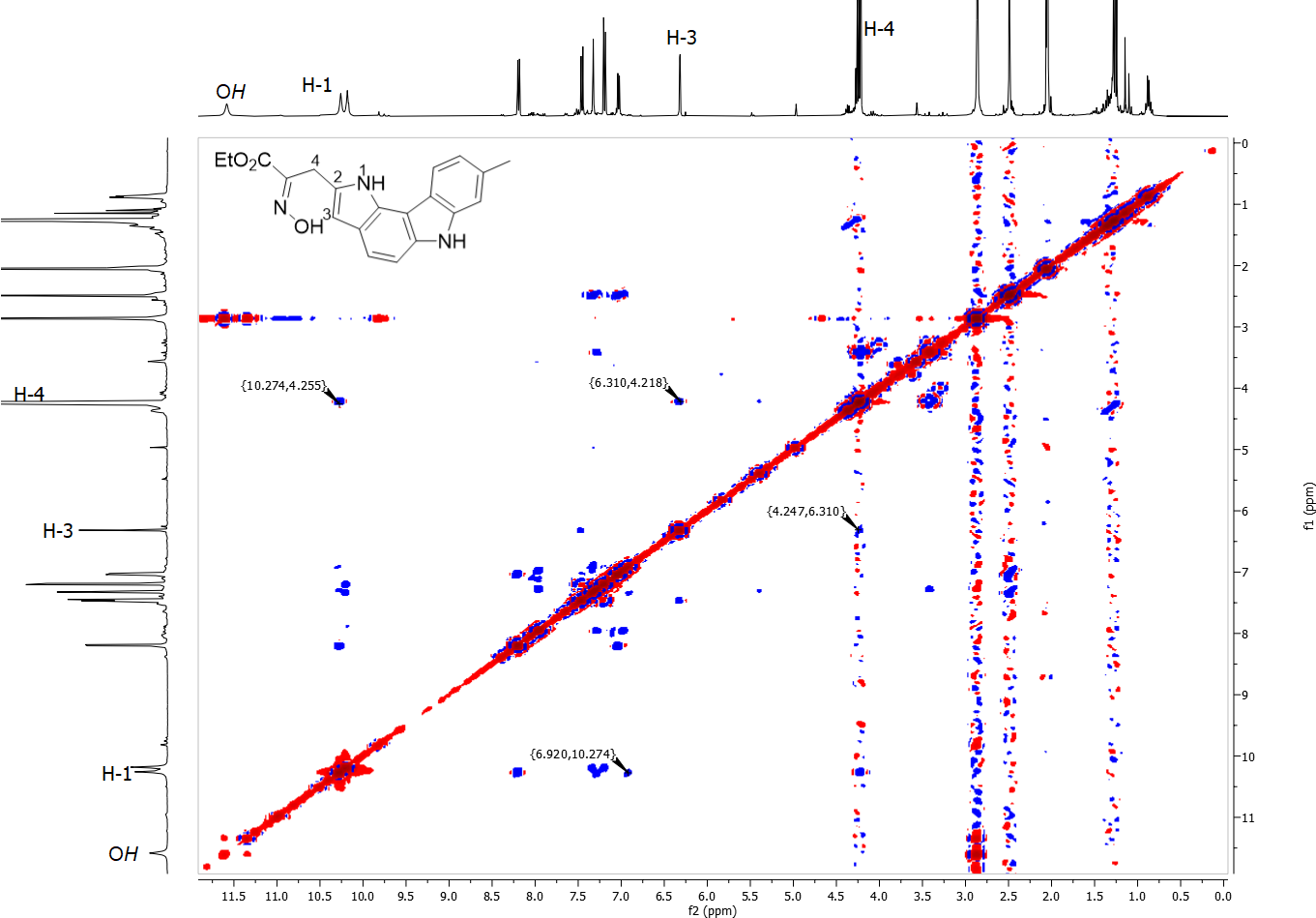


**Figure S10.** NOESY spectrum of compound **12a** (400 MHz, Acetone-*d_6_*).


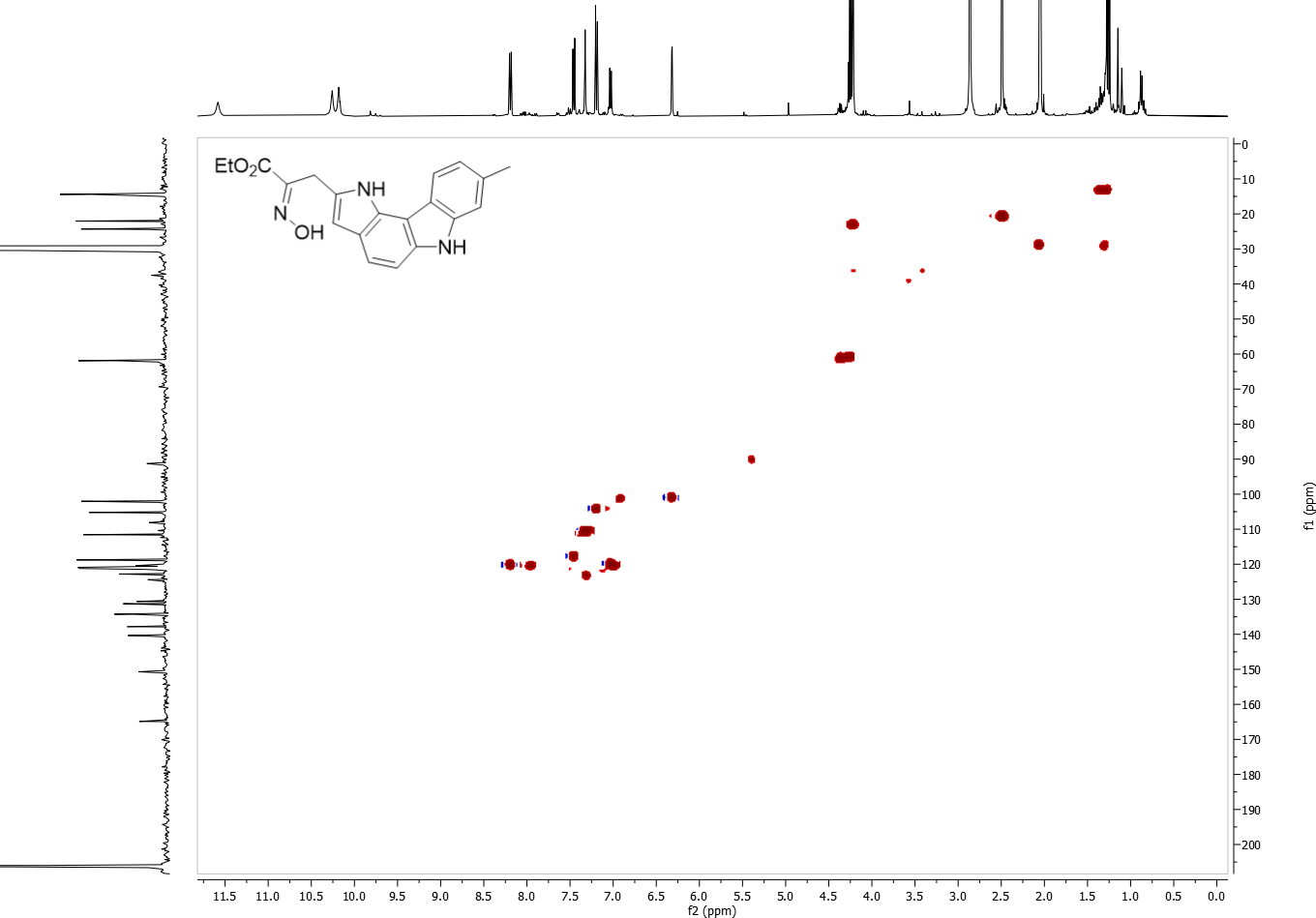


**Figure S11.** HSQC spectrum of compound **12a** (Acetone-*d_6_*).


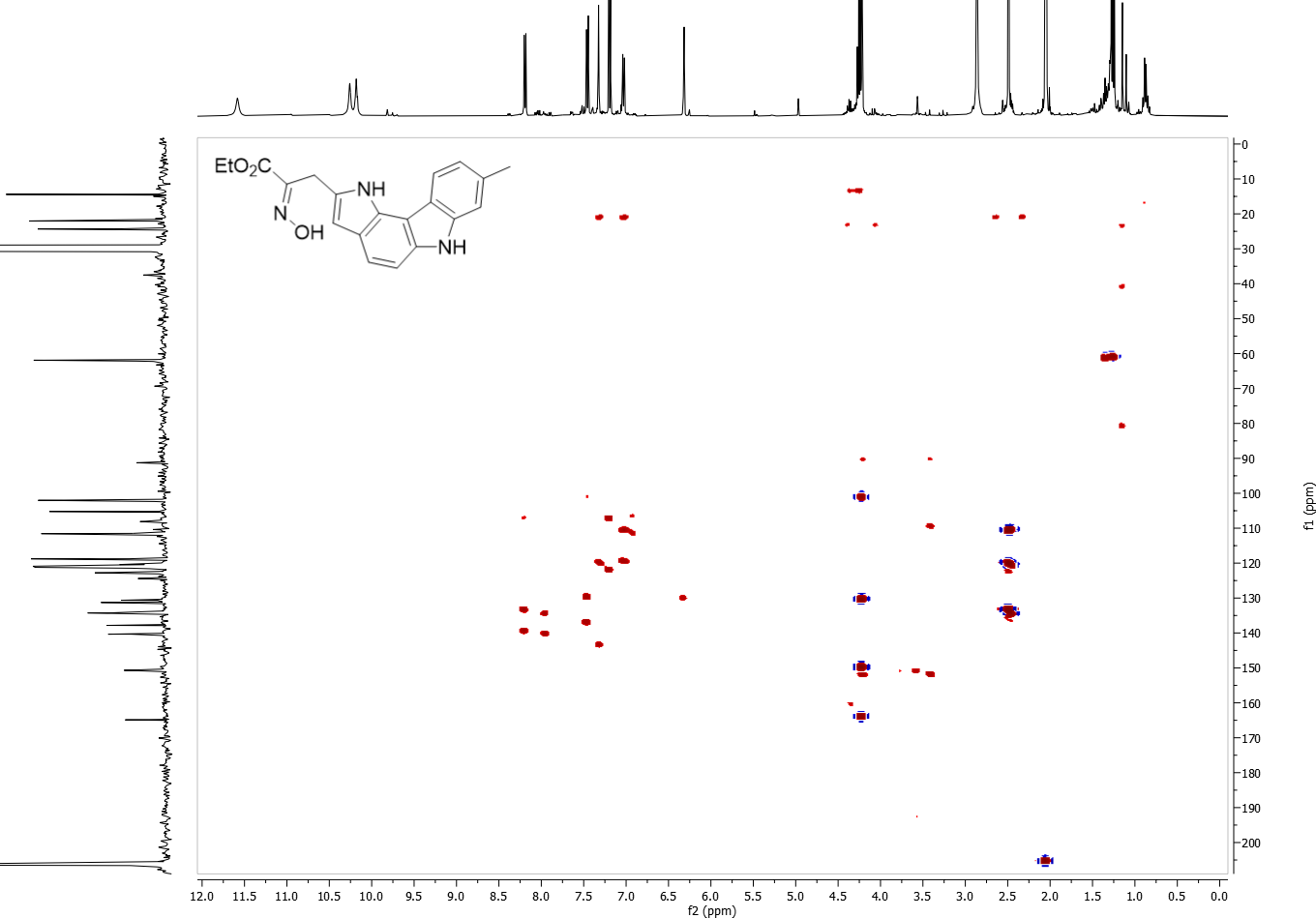


**Figure S12.** HMBC spectrum of compound **12a** (Acetone-*d_6_*).


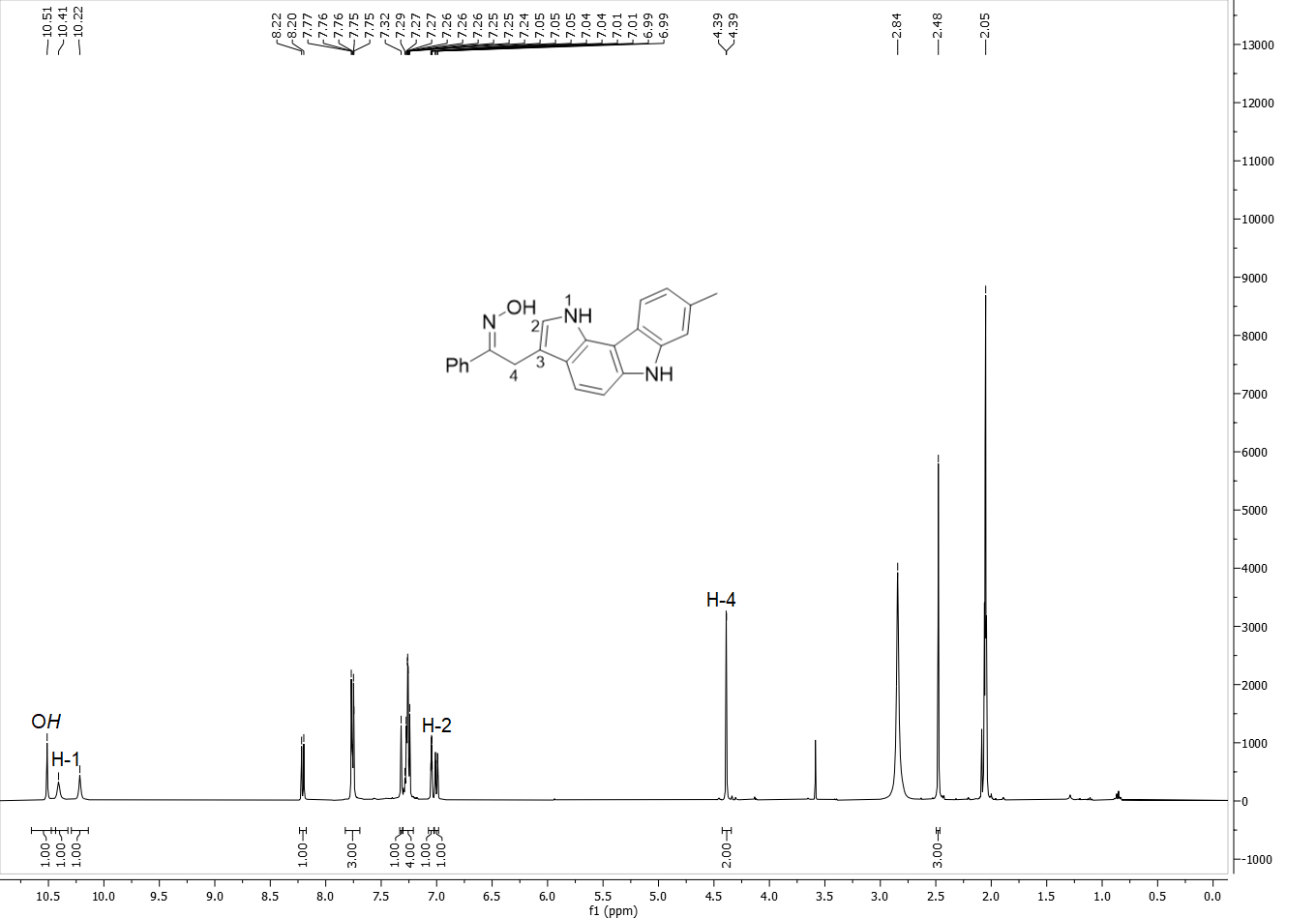


**Figure S13.** ^1^H NMR spectrum of compound **11b** (400 MHz, Acetone-*d_6_*).


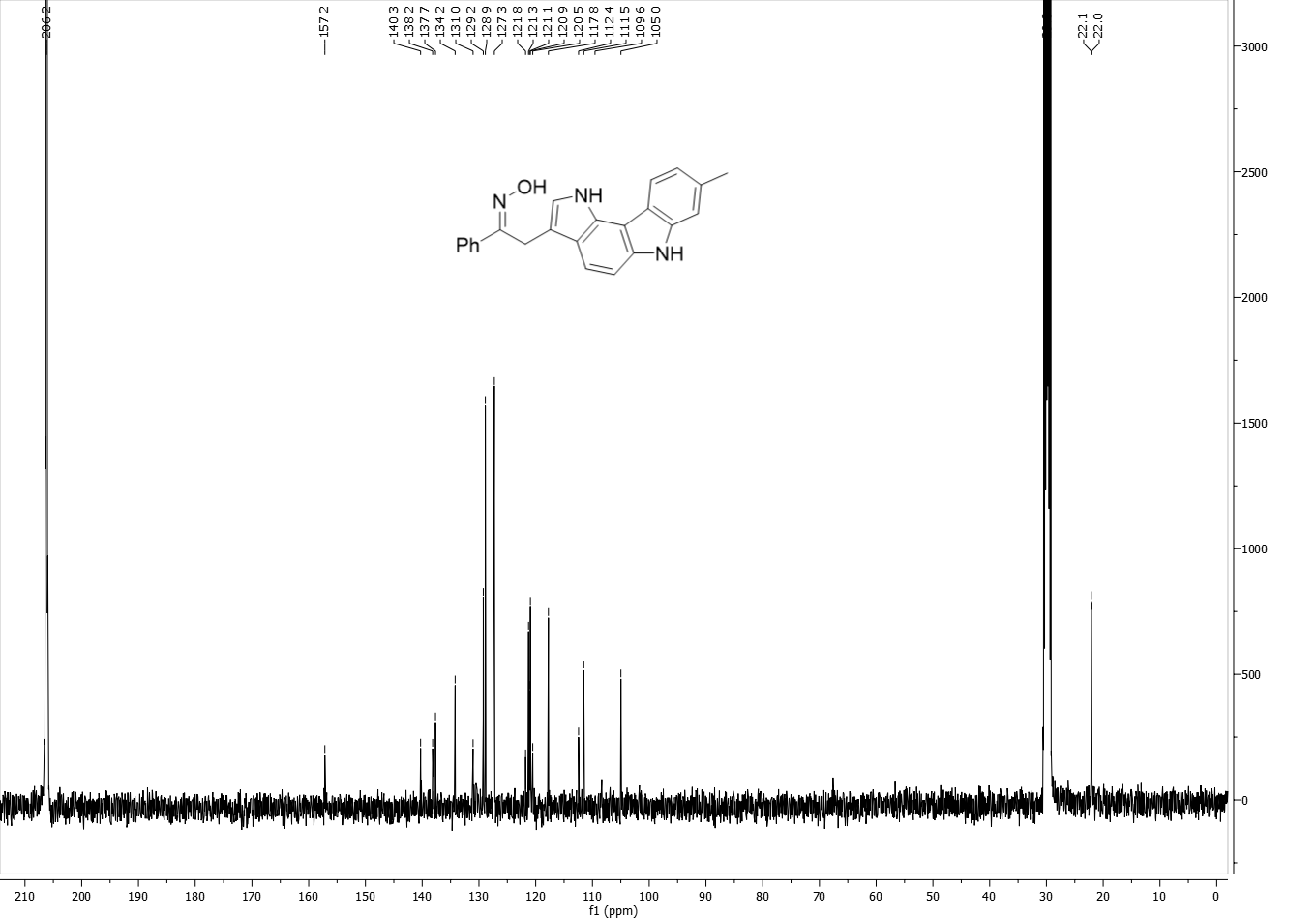


**Figure S14.** ^13^C NMR spectrum of compound **11b** (100 MHz, Acetone-*d_6_*).


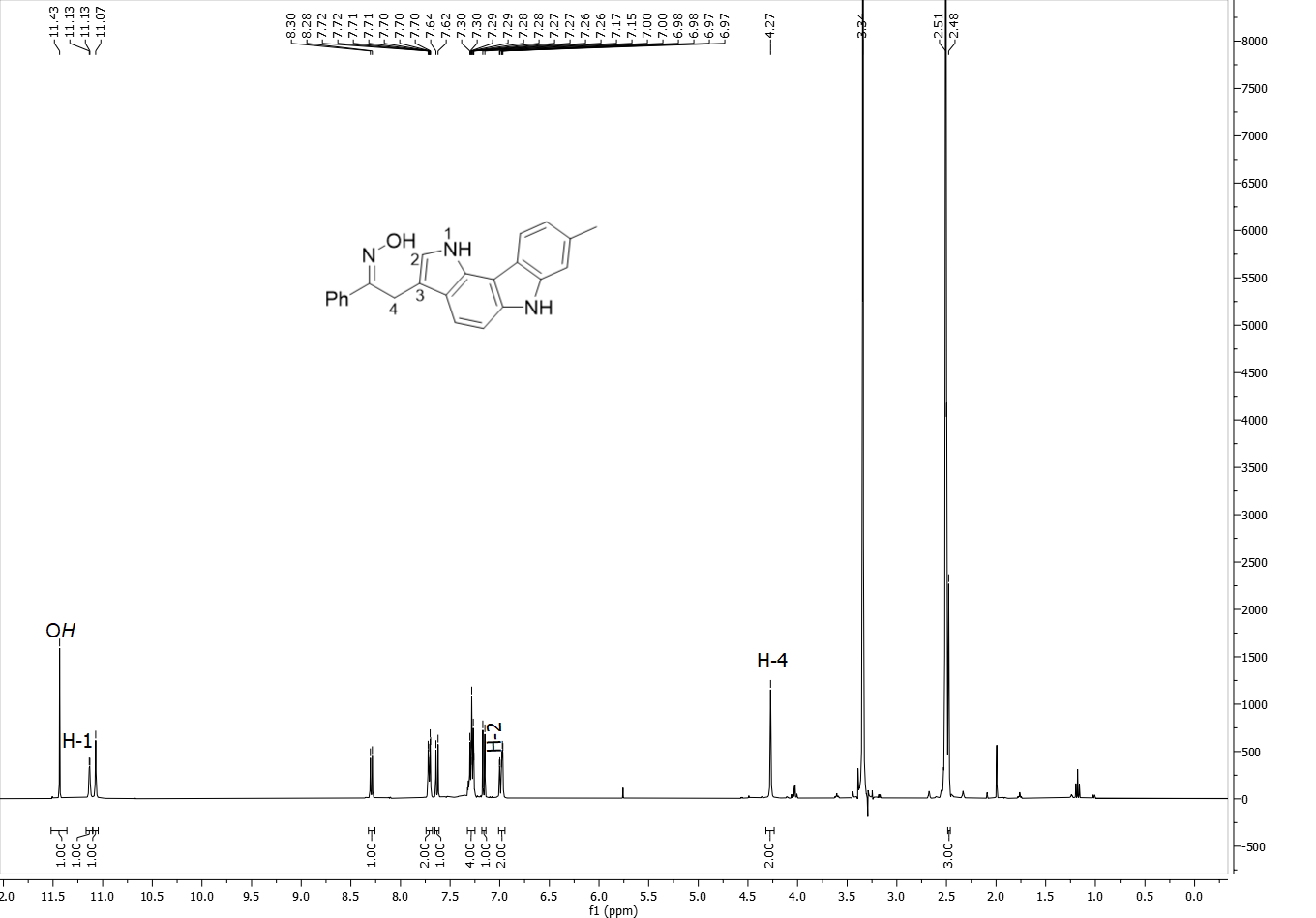


**Figure S15.** ^1^H NMR spectrum of compound **11b** (400 MHz, DMSO-*d_6_*).


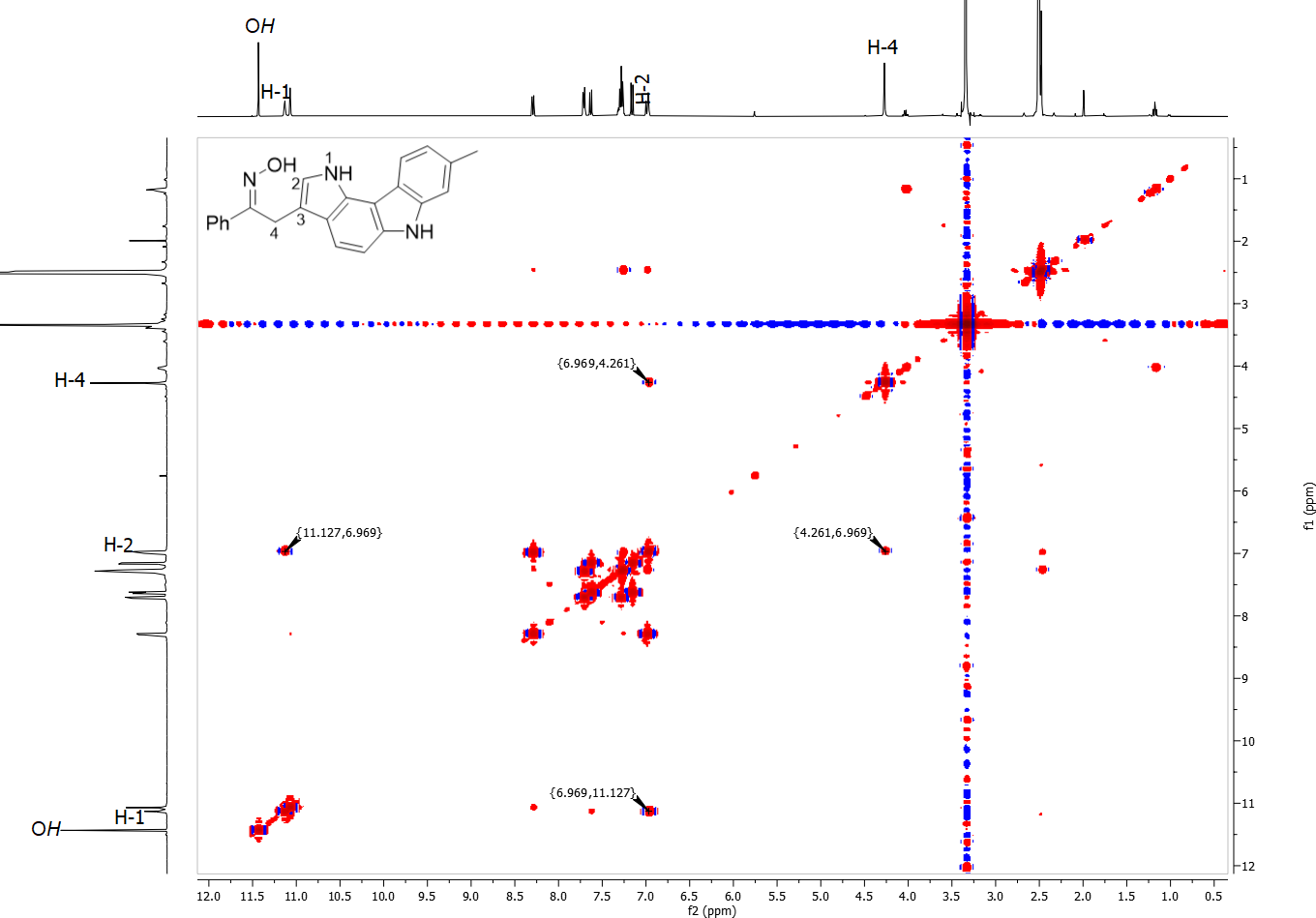


**Figure S16.** COSY spectrum of compound **11b** (400 MHz, DMSO-*d_6_*).


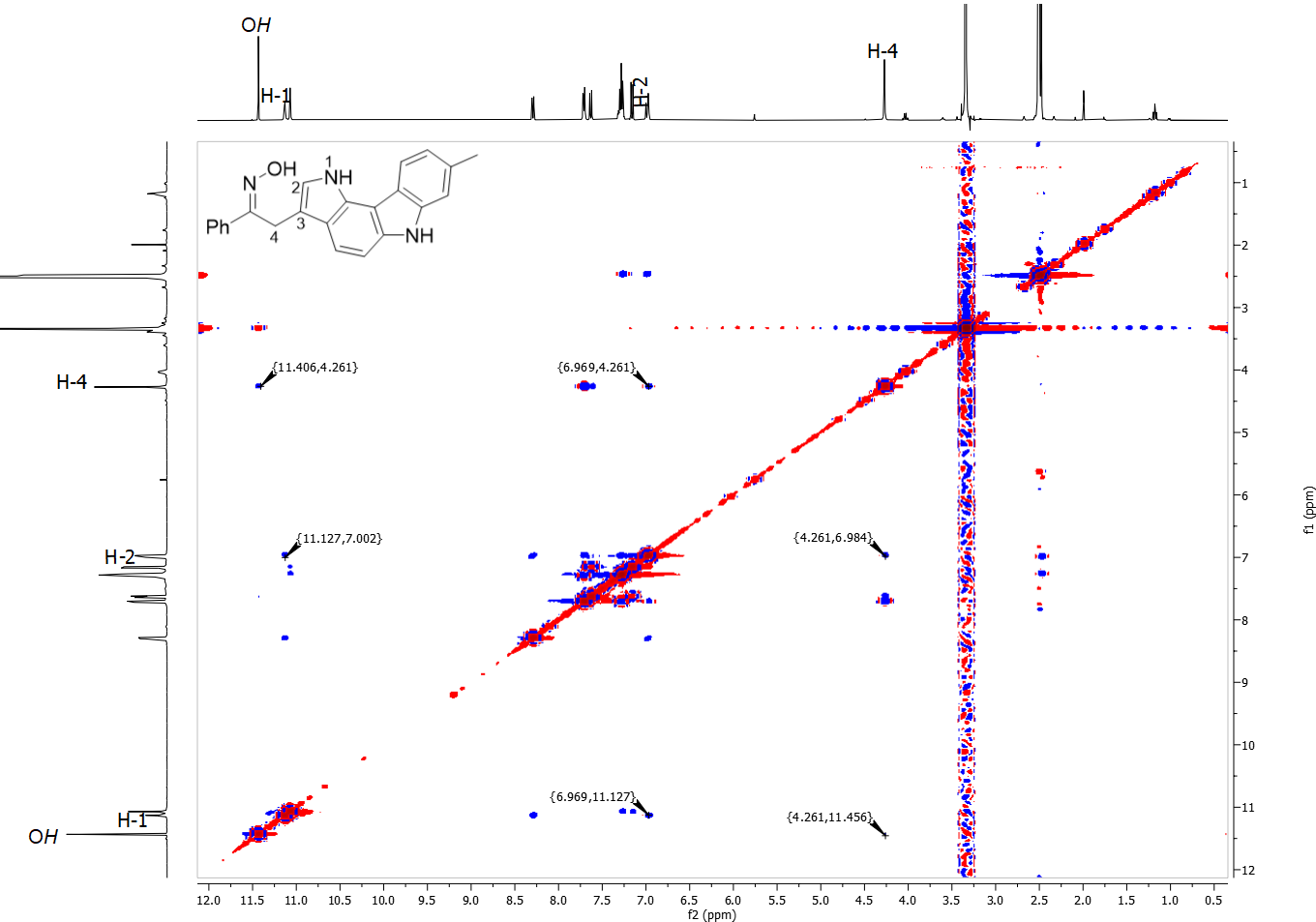


**Figure S17.** NOESY spectrum of compound **11b** (400 MHz, DMSO-*d_6_*).


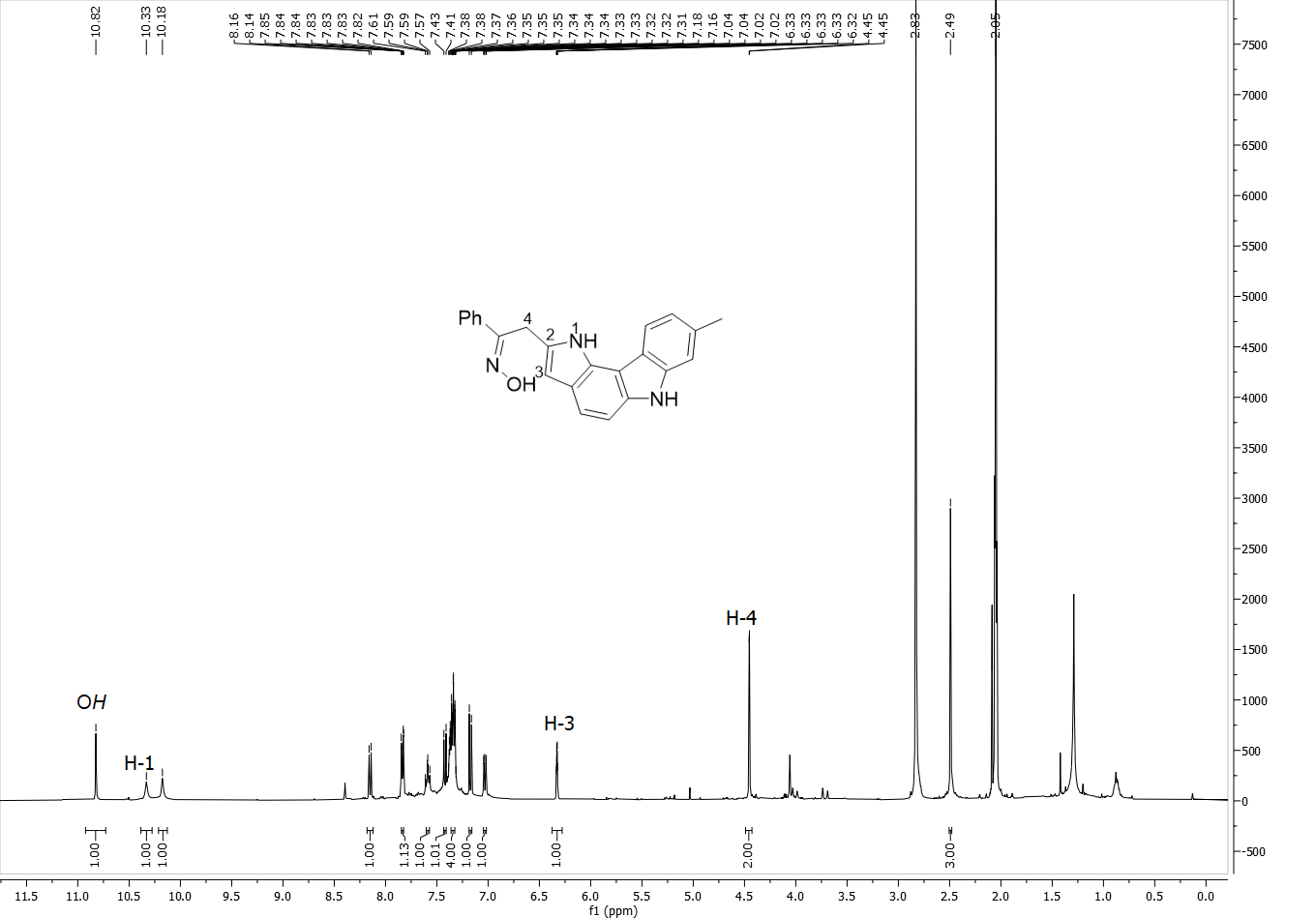


**Figure S18.** ^1^H NMR spectrum of compound **12b** (400 MHz, Acetone-*d_6_*).


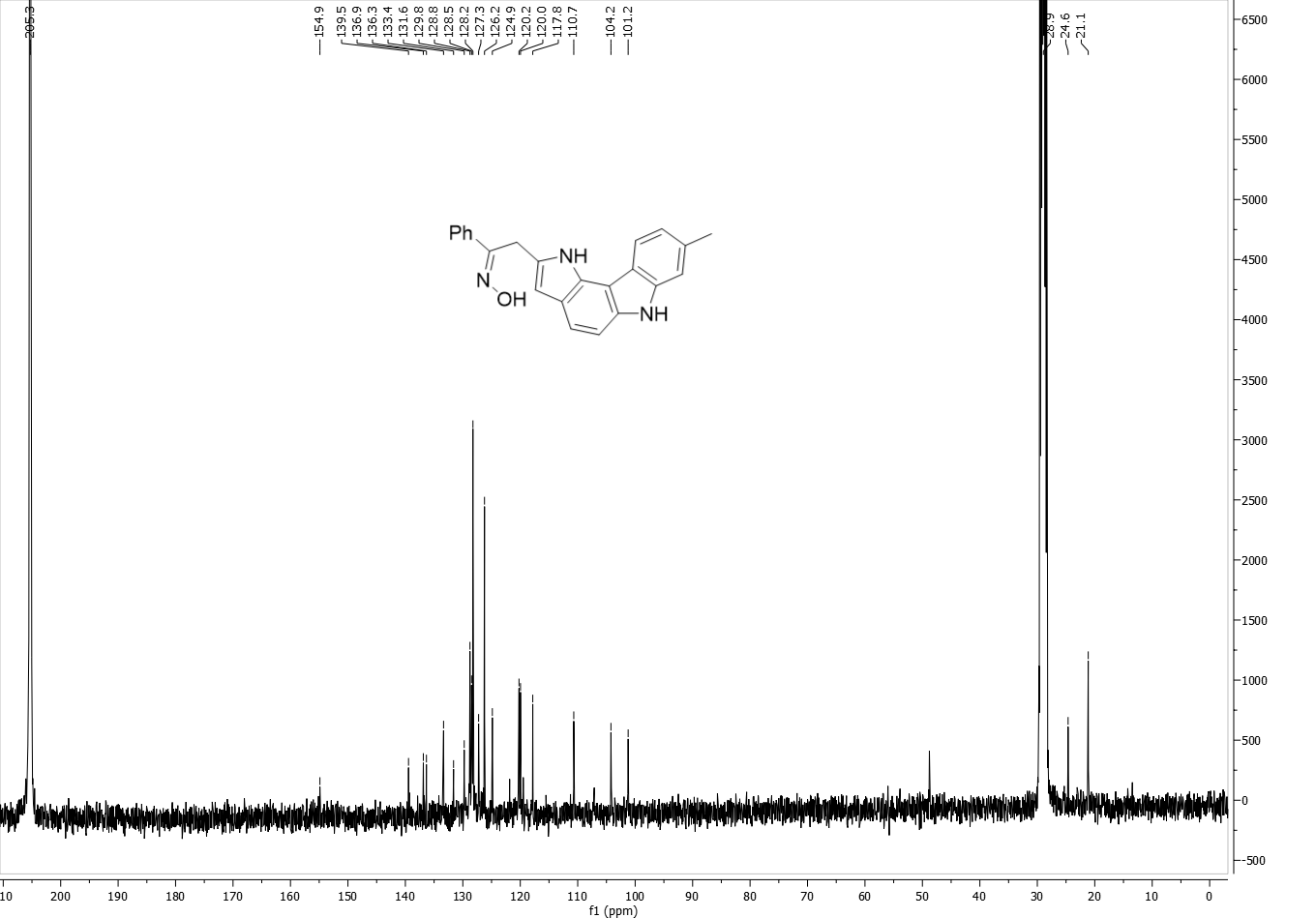


**Figure S19.** ^13^C NMR spectrum of compound **12b** (100 MHz, Acetone-*d_6_*).


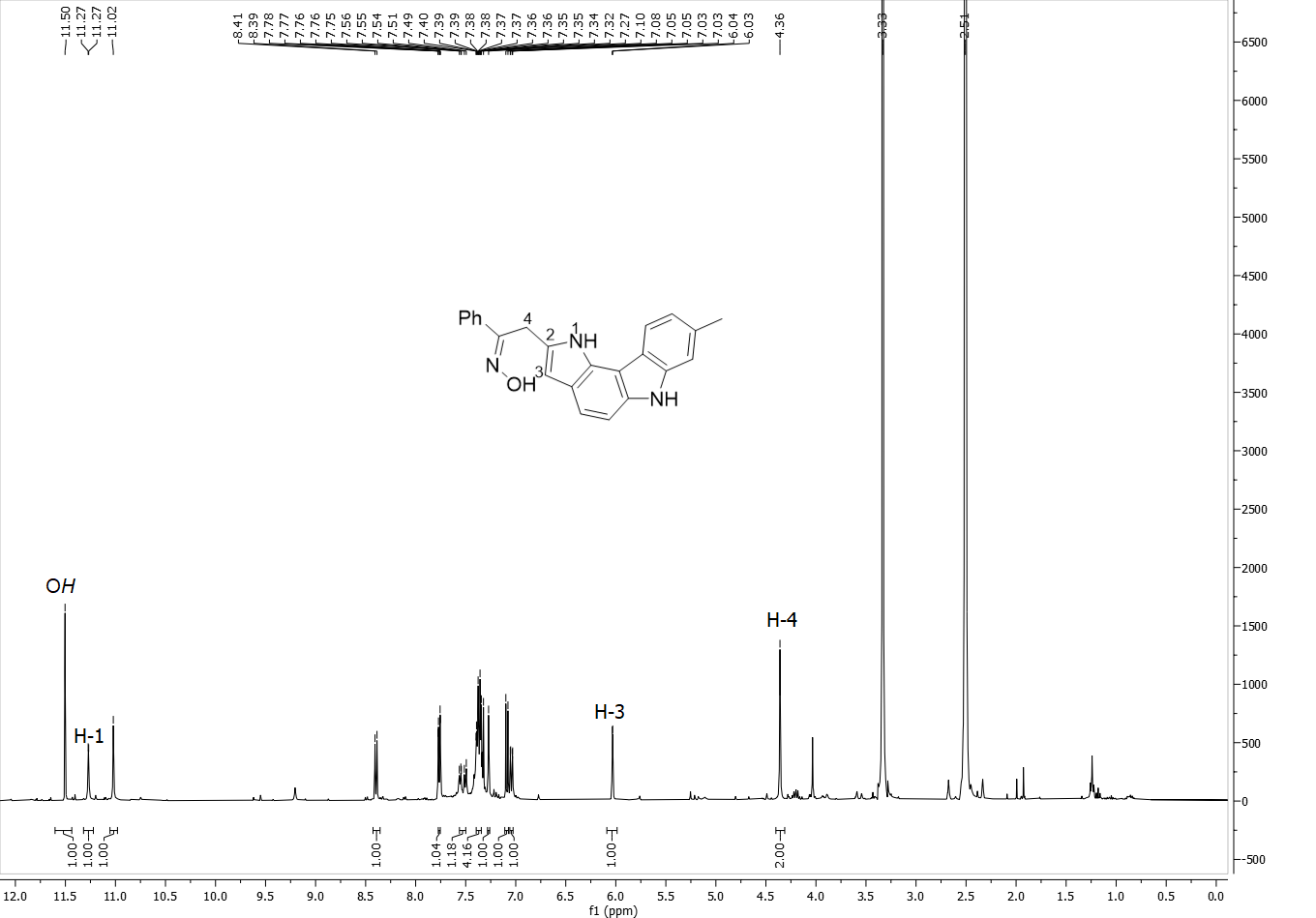


**Figure S20.** ^1^H NMR spectrum of compound **12b** (400 MHz, DMSO-*d_6_*).


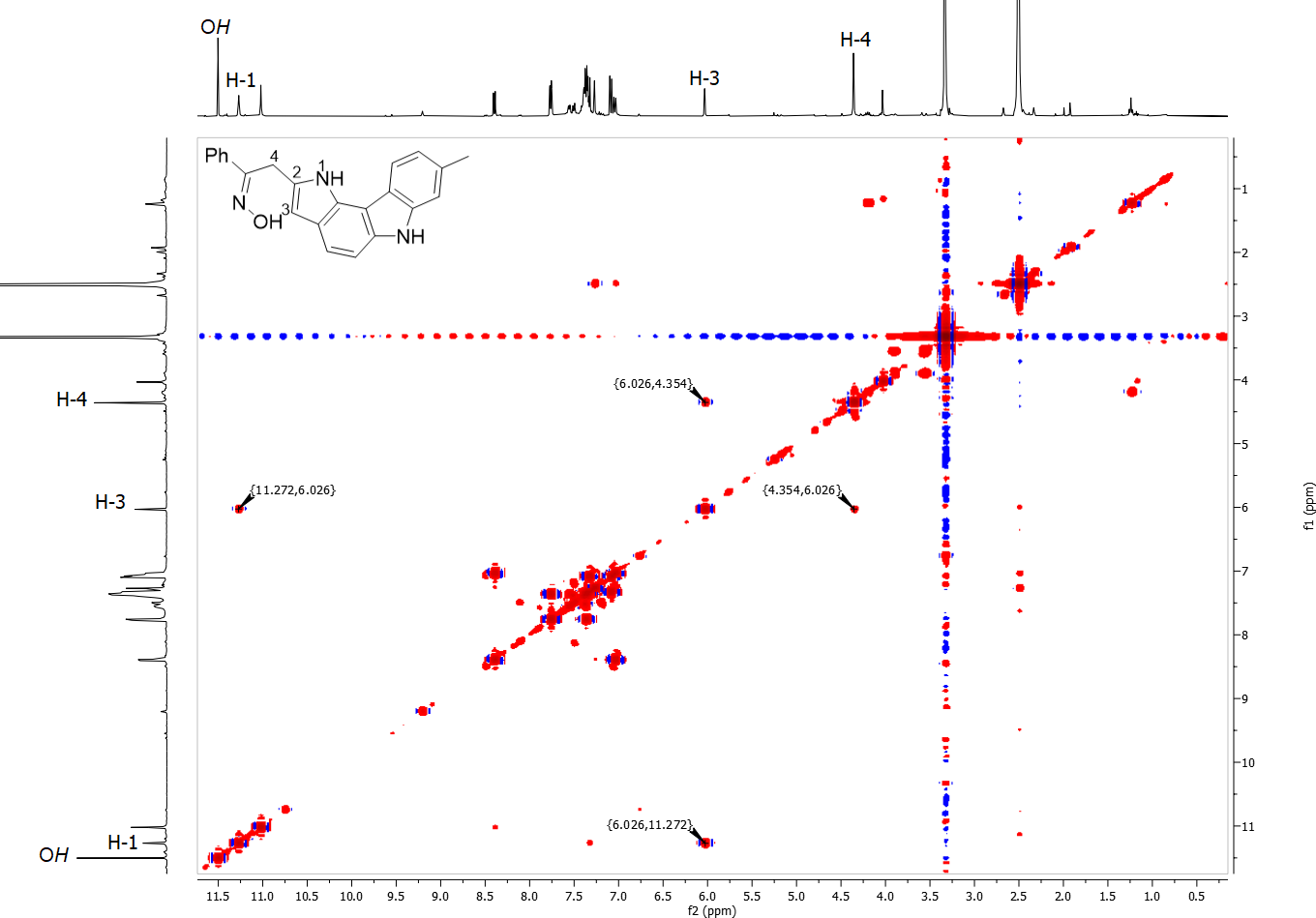


**Figure S21.** COSY spectrum of compound **12b** (400 MHz, DMSO-*d_6_*).


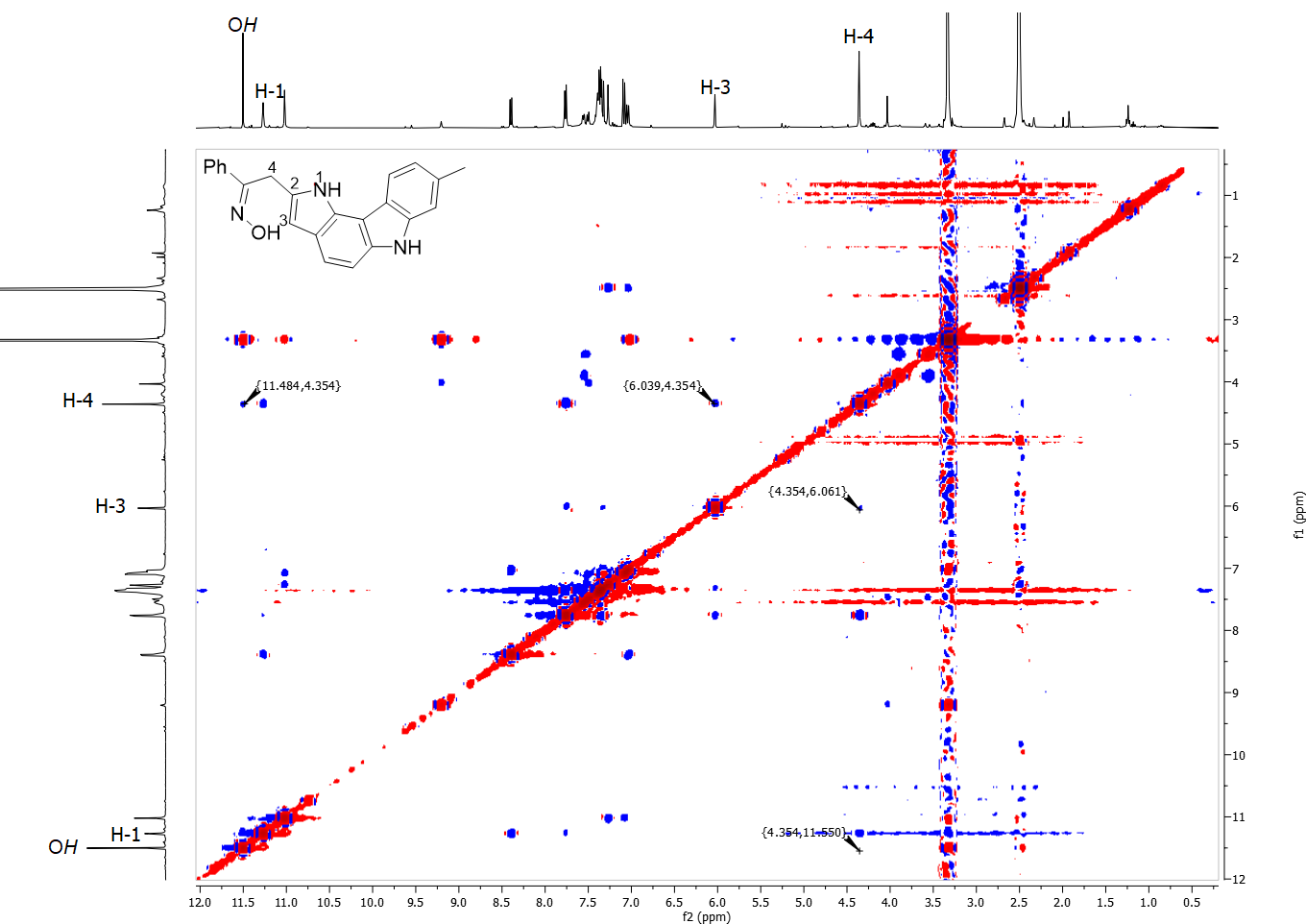


**Figure S22.** NOESY spectrum of compound **12b** (400 MHz, DMSO-*d_6_*).


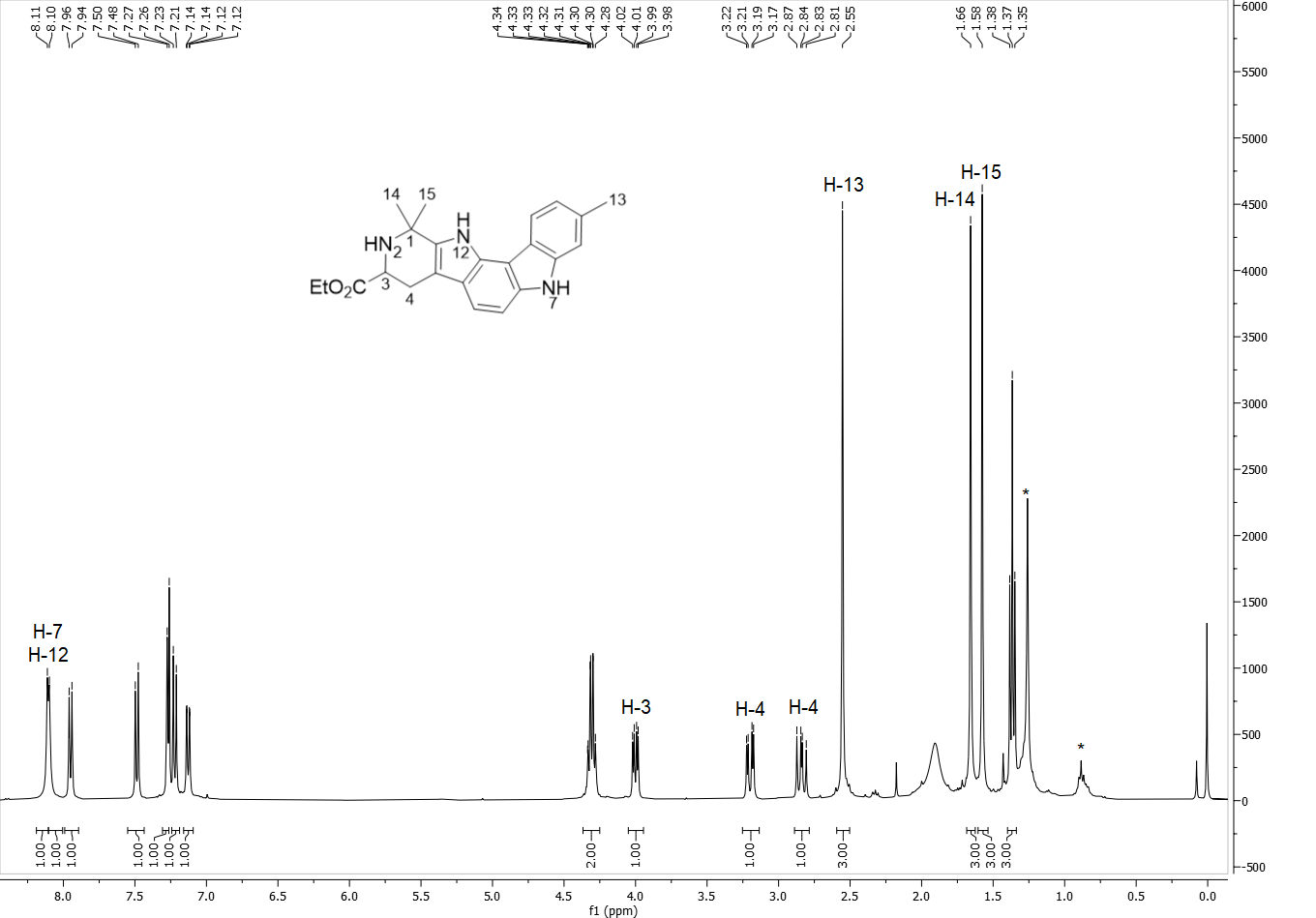


**Figure S23.** ^1^H NMR spectrum of compound **14** (400 MHz, CDCl_3_).


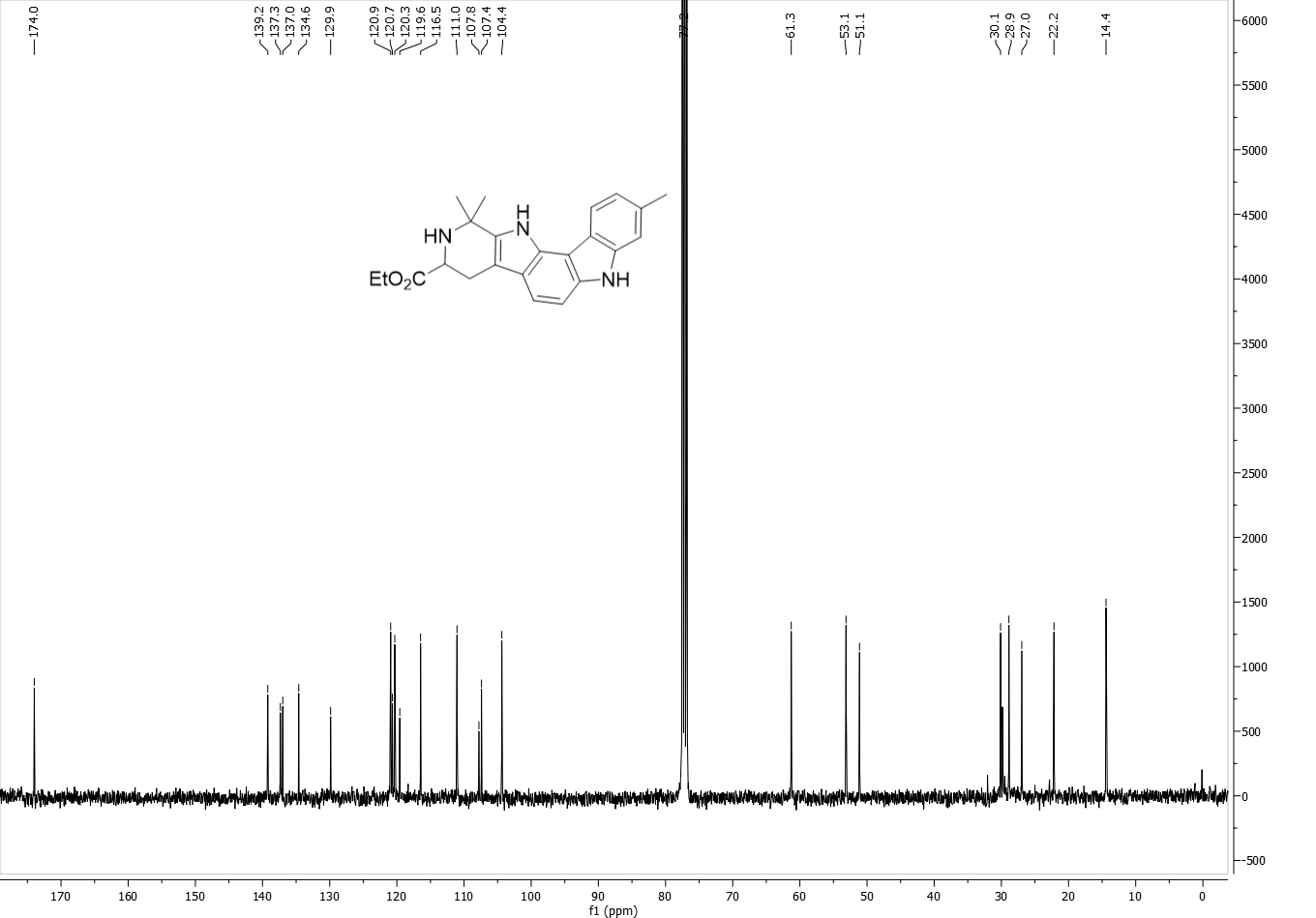


**Figure S24.** ^13^C NMR spectrum of compound **14** (100 MHz, CDCl_3_).


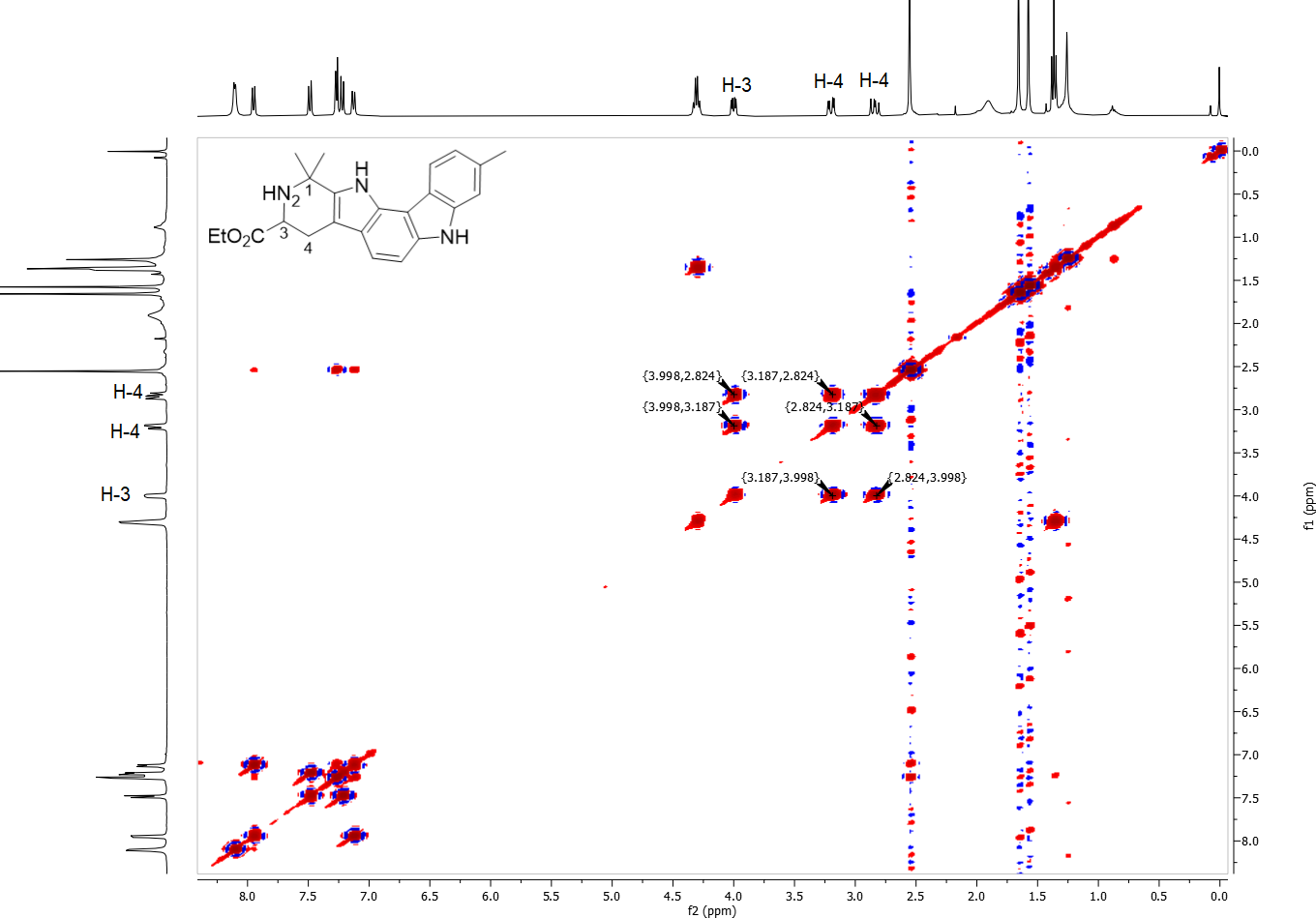


**Figure S25.** COSY spectrum of compound **14** (400 MHz, CDCl_3_).


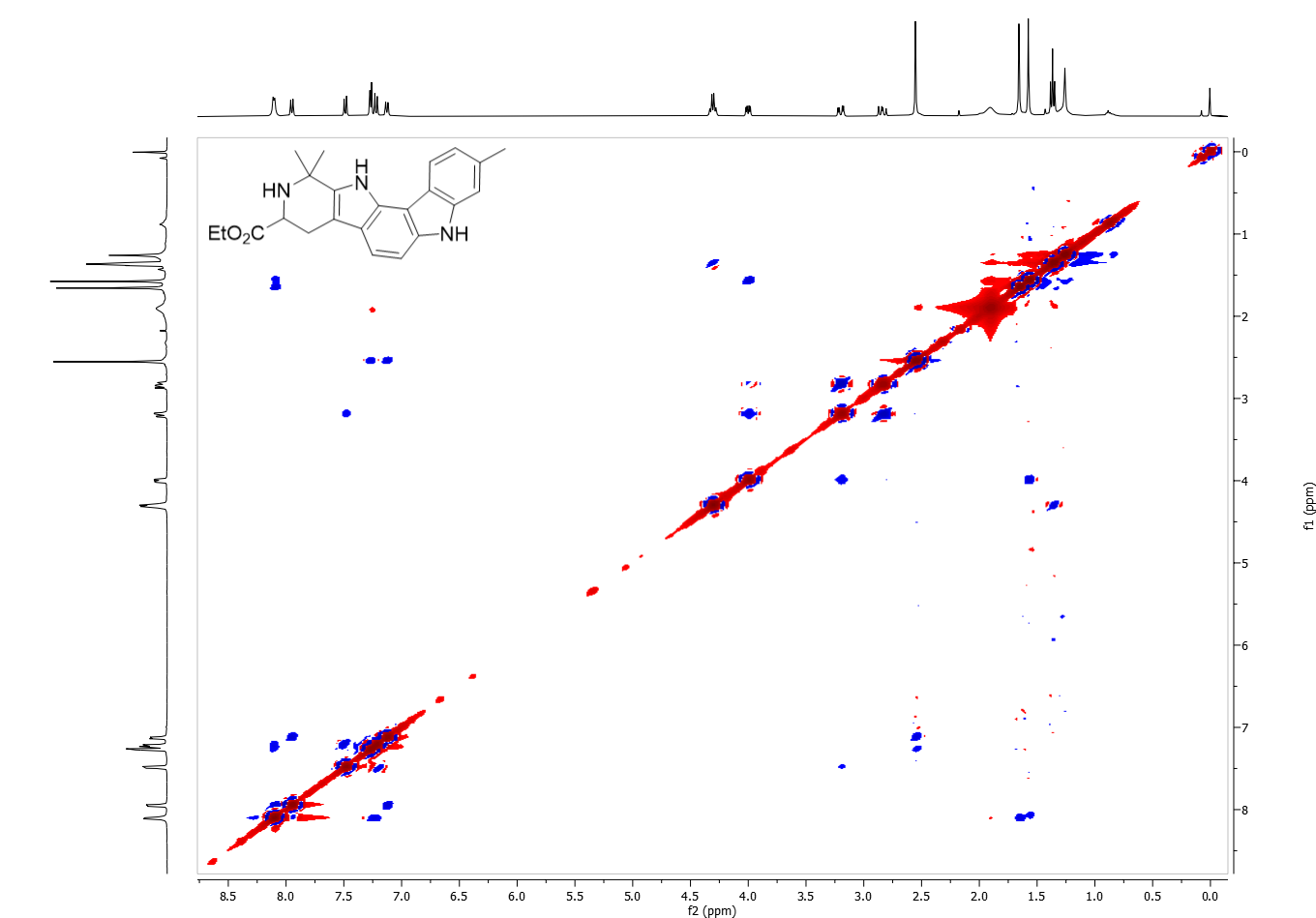


**Figure S26.** NOESY spectrum of compound **14** (400 MHz, CDCl_3_).


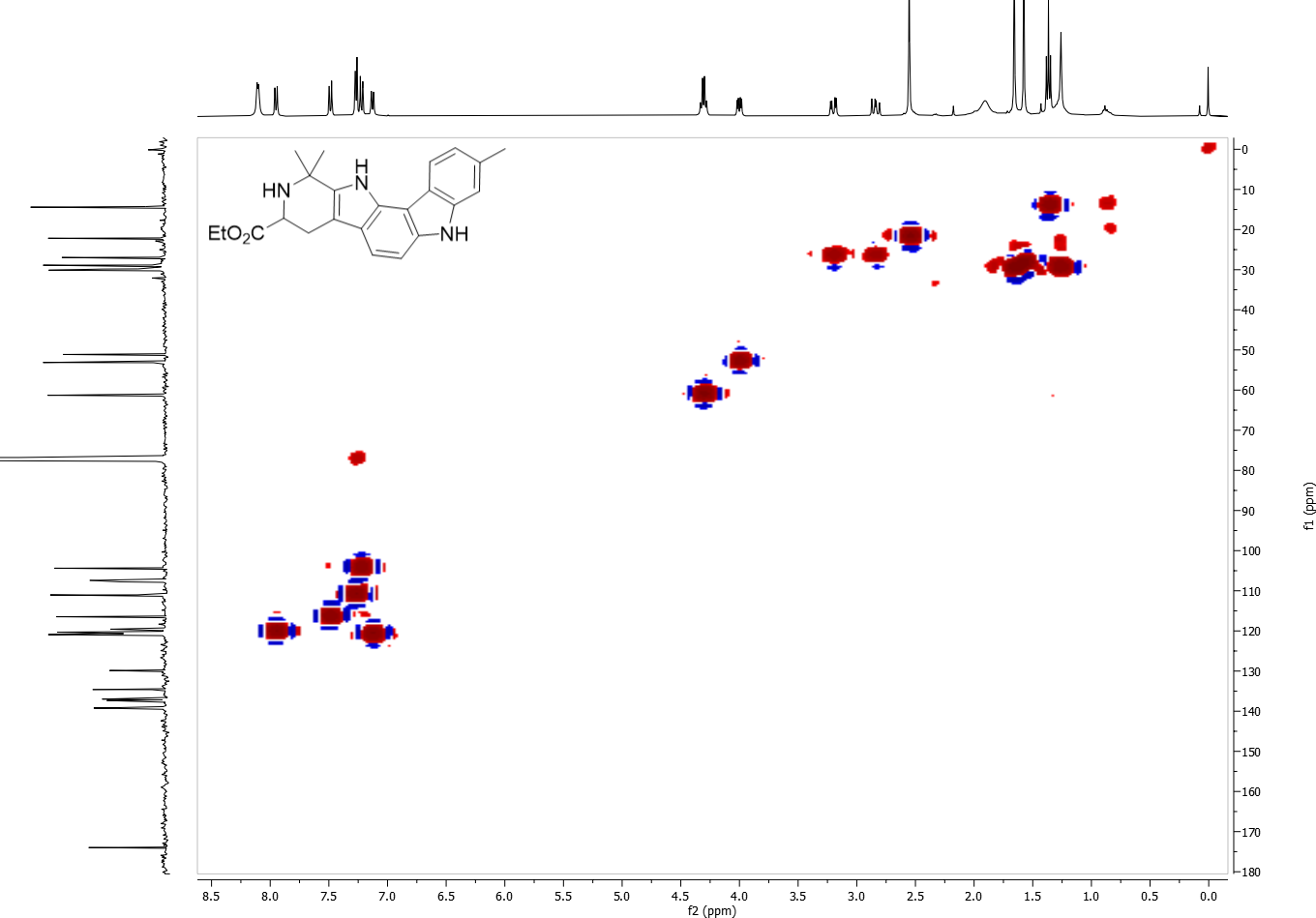


**Figure S27.** HSQC spectrum of compound **14** (CDCl_3_).


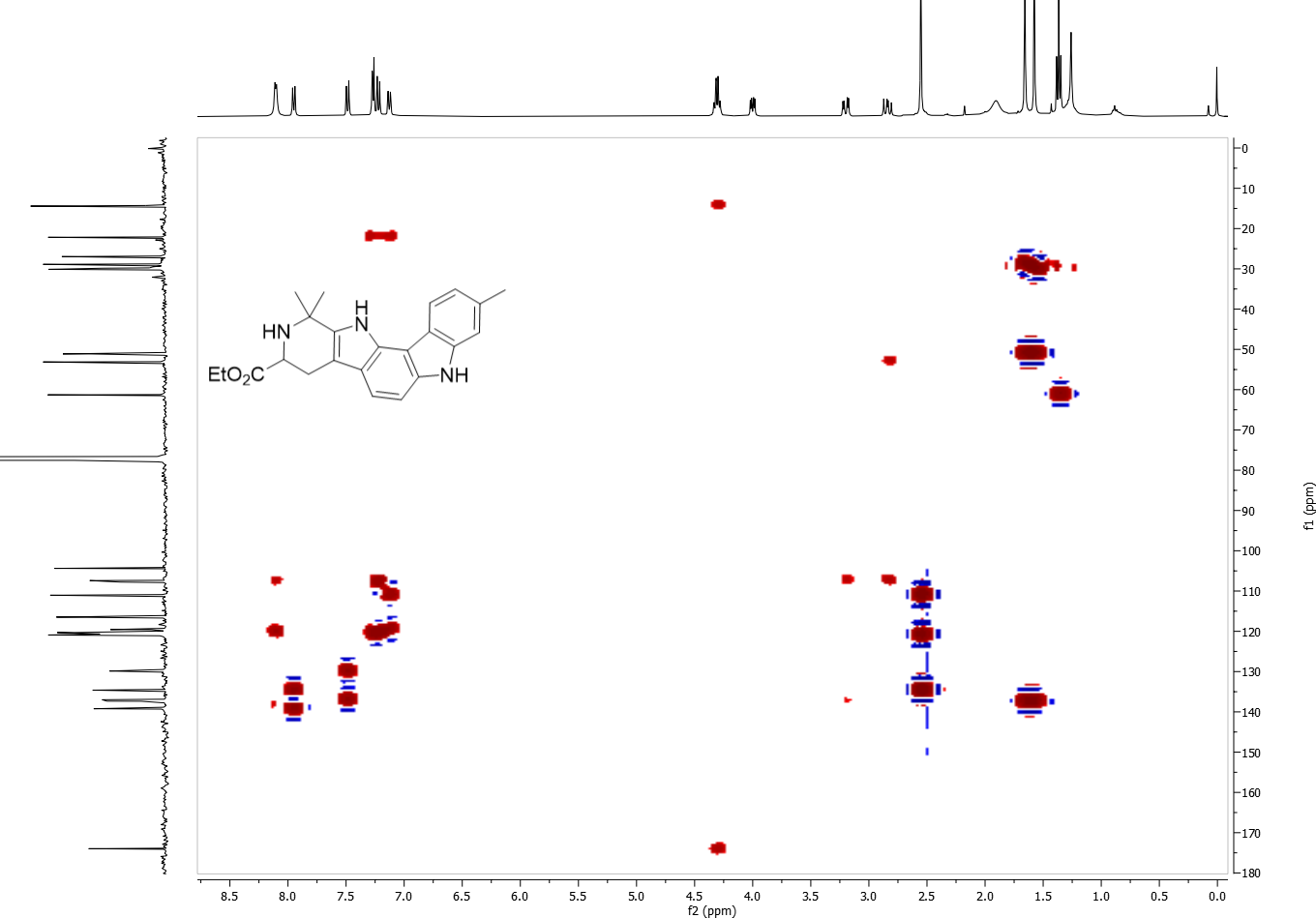


**Figure S28.** HMBC spectrum of compound **14** (CDCl_3_).

1. **X-Ray Crystallographic data for compound 14**

**Figure S29.** ORTEP-3 diagram of compound **14**, using 50% probability level ellipsoids.

**Table S1.** Crystallographic data and details about refinement for structure **14**.

|  | **14** |
| --- | --- |
| Formula | C_23_H_25_N_3_O_2_⋅H_2_O⋅C_2_H_5_OH |
| *M* | 439.54 |
| λ (Å) | 1.54178 |
| *T* (K) | 100(2) |
| crystal system | Triclinic |
| space group | *P1* |
| *a* (Å) | 8.6985(4) |
| *b* (Å) | 10.0013(4) |
| *c* (Å) | 14.4943(6) |
| *α* (°) | 71.400(2) |
| *β* (°) | 81.760(2) |
| *γ* (°) | 86.289(2) |
| *V* (Å^3^) | 1182.51(9) |
| *Z* | 2 |
| *ρ_calc_* (g.cm^-3^) | 1.234 |
| *µ* (mm^-1^) | 0.677 |
| Crystal size | 0.02×0.10×0.10 |
| Crystal colour | Colourless |
| Crystal description | Plates |
| *θ_max_* (°) | 63.43 |
| total data | 19021 |
| unique data | 3828 |
| *R_int_* | 0.1068 |
| *R* [*I*>2σ(*I*)] | 0.0850 |
| *R_w_* | 0.2348 |
| Goodness of fit | 1.153 |
| *ρ_min_* *ρ_max_* | -0.276  0.184 |

1. **Theoretical Calculations**

**Cartesian coordinates (Å) obtained from the B3LYP/6-31G(d,p) calculations**

Nitrosoalkene **2a**

| C | 0.1840610594 | 0.6769813997 | 0.3236184887 |
| --- | --- | --- | --- |
| C | -1.2091720415 | 0.1733638613 | 0.5414608859 |
| C | -2.2268259983 | 1.0289206514 | 0.7062157369 |
| H | -2.0521245802 | 2.0982123729 | 0.6723607034 |
| H | -3.2272634689 | 0.6476058215 | 0.8834200435 |
| N | -1.3826111222 | -1.2725832192 | 0.6800630768 |
| O | -2.5345483934 | -1.6452180872 | 0.5531831908 |
| C | 2.4490082929 | 0.0859472416 | -0.0095280607 |
| H | 2.4841003108 | 0.7157819500 | -0.9037357140 |
| H | 2.7753993571 | 0.6966343133 | 0.8383516504 |
| H | 4.3246477210 | -0.9106726347 | -0.3267008898 |
| C | 3.2758849922 | -1.1756932571 | -0.1588213181 |
| H | 2.9303226497 | -1.7716728812 | -1.0082072140 |
| H | 3.2156461860 | -1.7925951024 | 0.7422186163 |
| O | 0.4569474335 | 1.8578177259 | 0.2380634734 |
| O | 1.0748276019 | -0.3198301557 | 0.2163373304 |

Pyrrole

| H |  | 2.3023614867 | -1.5814320699 | -0.8693483714 |
| --- | --- | --- | --- | --- |
| C |  | 3.3145622710 | -1.2255313609 | -0.7458929721 |
| C |  | 3.9216979279 | -0.0579137172 | -1.1549430420 |
| N |  | 4.2568453030 | -1.9763422893 | -0.0822948911 |
| C |  | 5.2788123068 | -0.1083447674 | -0.7220360418 |
| C |  | 5.4573323374 | -1.3051473748 | -0.0623001035 |
| H |  | 3.4456796244 | 0.7429745019 | -1.7035355718 |
| H |  | 6.0369824683 | 0.6466493674 | -0.8775586640 |
| H |  | 6.3277579539 | -1.7312684921 | 0.4143796451 |
| H |  | 4.0927683206 | -2.8826437979 | 0.3246300127 |

*Exo* cycloadduct **3** – C2 alkylation

| C | 5.8374897241 | -2.4302226976 | 6.3919185594 |
| --- | --- | --- | --- |
| C | 5.5842329482 | -2.4829888524 | 4.9083423275 |
| H | 5.2241365675 | -3.4970588008 | 4.6914096872 |
| N | 4.9609278510 | -2.1376239707 | 7.2868707409 |
| O | 3.7150408355 | -1.7557606486 | 6.7704570881 |
| C | 2.1766457772 | -1.4583096266 | 4.1218746779 |
| C | 3.3687244922 | -2.0028104550 | 3.6200457624 |
| N | 2.4573133071 | -0.5806122997 | 5.1848294009 |
| C | 4.5250385304 | -1.4589843960 | 4.4390677457 |
| C | 3.7939056648 | -0.8282578021 | 5.6438509819 |
| H | 1.7591659226 | -0.4566918736 | 5.9081288739 |
| C | 7.1946035248 | -2.8671682754 | 6.8690803881 |
| C | 8.6057972002 | -3.3051211259 | 8.7105563930 |
| C | 8.5215737820 | -3.2826712118 | 10.2244033623 |
| H | 8.8177053250 | -4.3058857694 | 8.3210632977 |
| C | 0.9443785986 | -1.7640992637 | 3.5508538189 |
| C | 3.3411348700 | -2.8635704021 | 2.5325416845 |
| C | 0.9275660200 | -2.6380107825 | 2.4566753689 |
| H | 0.0226674204 | -1.3439570843 | 3.9430248101 |
| C | 2.1071947952 | -3.1851070798 | 1.9478918751 |
| H | 4.2606680940 | -3.2899645240 | 2.1388620502 |
| H | 0.0242294254 | -2.8945279870 | 1.9998211663 |
| H | 2.0698641236 | -3.8605042930 | 1.0987223983 |
| O | 8.0770452346 | -3.1846450424 | 6.0928701935 |
| O | 7.3207573610 | -2.8703737917 | 8.2018594510 |
| H | 9.3806104648 | -2.6319328604 | 8.3289576027 |
| H | 9.4779589842 | -3.5985078640 | 10.6529529770 |
| H | 8.2947471688 | -2.2761953640 | 10.5874512116 |
| H | 7.7410705935 | -3.9605589855 | 10.5815032978 |
| H | 6.5321982151 | -2.3745047405 | 4.3798166300 |
| H | 4.2820820597 | 0.0757811558 | 6.0248143681 |
| H | 5.0238839691 | -0.6522532855 | 3.8854818091 |

*Endo* cycloadduct **3** – C2 alkylation

| C | 4.8377684237 | -2.7841359955 | 6.7970771423 |
| --- | --- | --- | --- |
| C | 4.5584247868 | -3.3213541219 | 5.4180884285 |
| H | 4.0252496614 | -4.2690496906 | 5.5028325234 |
| N | 5.1133532230 | -1.5577719952 | 7.0713844556 |
| O | 5.0378684377 | -0.6949776854 | 5.9688414233 |
| C | 4.4957751693 | -0.6762395636 | 2.9261150741 |
| C | 4.4309959279 | -2.0610643621 | 3.1431373301 |
| N | 3.9462679776 | 0.0099759412 | 4.0231661115 |
| C | 3.8232294845 | -2.3050821037 | 4.5123271639 |
| C | 3.8535441758 | -0.8916688095 | 5.1352678969 |
| H | 4.2837937768 | 0.9409188445 | 4.2350087519 |
| C | 4.9253566271 | -3.7821315624 | 7.9177187744 |
| C | 5.3541054726 | -4.1629176952 | 10.2079293553 |
| C | 5.6488885265 | -3.3453309549 | 11.4501686054 |
| H | 4.4296066901 | -4.7415808507 | 10.3001188566 |
| C | 5.0000725231 | -0.1521219360 | 1.7387657710 |
| C | 4.8694219982 | -2.9430753873 | 2.1662602104 |
| C | 5.4444558700 | -1.0514921253 | 0.7616664959 |
| H | 5.0526608287 | 0.9203516620 | 1.5747342691 |
| C | 5.3823593678 | -2.4314731666 | 0.9650115044 |
| H | 4.8219192191 | -4.0174531874 | 2.3266957415 |
| H | 5.8459118062 | -0.6634323292 | -0.1704279499 |
| H | 5.7322009203 | -3.1100056720 | 0.1930736615 |
| O | 4.7468628730 | -4.9722540686 | 7.7340908939 |
| O | 5.2160913964 | -3.2341872117 | 9.1041388541 |
| H | 6.1612071653 | -4.8650454288 | 9.9748681129 |
| H | 5.7675169520 | -4.0107138644 | 12.3112410878 |
| H | 6.5704184763 | -2.7692992882 | 11.3273507649 |
| H | 4.8332128495 | -2.6481330334 | 11.6613804436 |
| H | 5.5349452524 | -3.5535898608 | 4.9736467753 |
| H | 2.7731930664 | -2.6083411651 | 4.4065908317 |
| H | 2.9894210745 | -0.6798233323 | 5.7748306387 |

Open chain oxime **4** – C2 alkylation

| C | 6.4860983794 | -0.7788180586 | 6.7104248322 |
| --- | --- | --- | --- |
| C | 5.8501295943 | -0.4517992472 | 5.3745585007 |
| H | 6.4670421846 | -0.9172924701 | 4.6003358421 |
| N | 6.1033257126 | -0.3562708928 | 7.8675458748 |
| O | 4.9797289604 | 0.4265395829 | 7.9093372636 |
| C | 2.2680101302 | -1.0754096732 | 4.3754285153 |
| C | 3.5019838078 | -0.3936164976 | 4.1955754743 |
| N | 2.4241766877 | -1.9216440300 | 5.4554267174 |
| C | 4.4033255455 | -0.8625422846 | 5.2232692855 |
| C | 3.7018161184 | -1.7890141366 | 5.9607612495 |
| H | 1.7066483219 | -2.5096793360 | 5.8469772279 |
| C | 7.7282880863 | -1.6216204599 | 6.6811418190 |
| C | 9.4325572228 | -2.6933303900 | 7.9118784551 |
| C | 9.8302771097 | -2.8820505292 | 9.3630277434 |
| H | 9.2304727305 | -3.6460696388 | 7.4115306210 |
| C | 1.1617560500 | -0.8484471264 | 3.5492945433 |
| C | 3.6205103722 | 0.5337625197 | 3.1459534874 |
| C | 1.3083953499 | 0.0779188924 | 2.5235888550 |
| H | 0.2242658691 | -1.3758022973 | 3.7017739201 |
| C | 2.5251195388 | 0.7616785988 | 2.3226634409 |
| H | 4.5537472209 | 1.0643494105 | 2.9784340563 |
| H | 0.4699567385 | 0.2777414851 | 1.8629574690 |
| H | 2.6042510277 | 1.4765783979 | 1.5090463292 |
| O | 8.2229425005 | -2.0142355111 | 5.6393174307 |
| O | 8.2263999090 | -1.8930474939 | 7.8958730790 |
| H | 10.2068236829 | -2.1774539393 | 7.3348091381 |
| H | 10.7393743131 | -3.4890602596 | 9.4210699819 |
| H | 10.0253943276 | -1.9190090763 | 9.8433732082 |
| H | 9.0389333299 | -3.3897155875 | 9.9218645800 |
| H | 5.9309844974 | 0.6349823557 | 5.2198026419 |
| H | 4.5101013004 | 0.3741044858 | 7.0500701227 |
| H | 4.0188633800 | -2.3699267928 | 6.8155882945 |

*Endo* cycloadduct **3’** – C3 alkylation

| C | 6.1750259147 | -1.5881068104 | 5.7801762067 |
| --- | --- | --- | --- |
| C | 5.6956215989 | -2.4842160621 | 4.6703422547 |
| H | 6.2792350151 | -3.4054550801 | 4.6571262696 |
| N | 5.5426768580 | -0.5399089516 | 6.1752246827 |
| O | 4.3062761663 | -0.3568584983 | 5.5332810870 |
| C | 2.3288424385 | -2.0371459107 | 3.4964783867 |
| C | 2.2575561998 | -1.2905267725 | 4.6830511821 |
| N | 3.5273439042 | -2.7536936857 | 3.4240331762 |
| C | 4.1768377500 | -2.7285122424 | 4.7488983839 |
| C | 3.4872520570 | -1.5602925938 | 5.4987197693 |
| H | 3.5040947266 | -3.6476555850 | 2.9505230427 |
| C | 7.5136746012 | -1.8793431972 | 6.3956962125 |
| C | 9.1801527133 | -1.2154844863 | 7.9284367236 |
| C | 9.4302651602 | -0.0823213249 | 8.9038224307 |
| H | 9.1617384413 | -2.1917903371 | 8.4236338153 |
| C | 1.2977817126 | -1.9744508420 | 2.5559814129 |
| C | 1.1790528451 | -0.4543205154 | 4.9386071116 |
| C | 0.2104679614 | -1.1382765952 | 2.8271226471 |
| H | 1.3404983280 | -2.5518903999 | 1.6369445221 |
| C | 0.1435367775 | -0.3790956901 | 4.0005145974 |
| H | 1.1413584912 | 0.1274897653 | 5.8553392590 |
| H | -0.6000629968 | -1.0782312278 | 2.1057494283 |
| H | -0.7113169809 | 0.2641437887 | 4.1835624240 |
| O | 8.1732781665 | -2.8503531702 | 6.0720861225 |
| O | 7.8893200036 | -0.9809249112 | 7.3142629576 |
| H | 9.9403583657 | -1.2603720443 | 7.1419813778 |
| H | 10.4026349231 | -0.2197643598 | 9.3873632989 |
| H | 9.4328579142 | 0.8824406403 | 8.3889629257 |
| H | 8.6595500986 | -0.0558211685 | 9.6795448749 |
| H | 5.8849532296 | -1.9534701165 | 3.7286606514 |
| H | 3.9750660681 | -3.6601837284 | 5.2960725566 |
| H | 3.2873715474 | -1.8245078866 | 6.5443002084 |

*Exo* cycloadduct **3’** – C3 alkylation

| C | 6.0746021208 | -1.6446487395 | 5.7731659924 |
| --- | --- | --- | --- |
| C | 5.4047385611 | -2.5049244659 | 4.7443311769 |
| H | 4.6314033748 | -3.1000966460 | 5.2494536617 |
| N | 5.4667804539 | -0.6716612215 | 6.3574299218 |
| O | 4.1394753610 | -0.5129216413 | 5.9431248794 |
| C | 2.4311869592 | -1.9829345617 | 3.3356179033 |
| C | 2.4562793825 | -0.9306926871 | 4.2631055940 |
| N | 3.7175521074 | -2.2750898331 | 2.8706430564 |
| C | 4.7062812323 | -1.5913960374 | 3.7212594832 |
| C | 3.8847232385 | -0.5251992830 | 4.5035637373 |
| H | 3.9189866697 | -3.2378960721 | 2.6296518888 |
| C | 7.4926336337 | -1.9394118281 | 6.1622646803 |
| C | 9.3400421129 | -1.3506249460 | 7.5116743798 |
| C | 9.6981067106 | -0.2816508274 | 8.5254625721 |
| H | 9.3882711695 | -2.3580180433 | 7.9380067631 |
| C | 1.2206200871 | -2.5650753692 | 2.9521167225 |
| C | 1.2811110140 | -0.4373170211 | 4.8151737532 |
| C | 0.0434002229 | -2.0684307315 | 3.5194163016 |
| H | 1.1926292186 | -3.3802941055 | 2.2347713209 |
| C | 0.0627982230 | -1.0155136763 | 4.4403788504 |
| H | 1.3140547504 | 0.3694554520 | 5.5415721073 |
| H | -0.9065593341 | -2.5128811995 | 3.2347579942 |
| H | -0.8662549643 | -0.6504318332 | 4.8667985191 |
| O | 8.1147281055 | -2.8656351814 | 5.6752276508 |
| O | 7.9810826521 | -1.0950231441 | 7.0787277857 |
| H | 9.9985895596 | -1.3300161972 | 6.6374592002 |
| H | 10.7158023638 | -0.4476414422 | 8.8929884535 |
| H | 9.6510282373 | 0.7146253177 | 8.0764603435 |
| H | 9.0131452095 | -0.3074417200 | 9.3776187465 |
| H | 6.1276782298 | -3.1803850860 | 4.2843150303 |
| H | 4.1121580766 | 0.4885826609 | 4.1516758954 |
| H | 5.4649252600 | -1.1132098900 | 3.0932856344 |

Open chain oxime **4’** – C3 alkylation

| C | 6.0152128370 | -2.1611373405 | 6.9858664038 |
| --- | --- | --- | --- |
| C | 5.1397532444 | -2.5974005736 | 5.8290261618 |
| H | 4.4285532361 | -3.3476718309 | 6.2095971848 |
| N | 5.6293195461 | -1.6095491634 | 8.0848274753 |
| O | 4.3016076707 | -1.3009859160 | 8.2088684431 |
| C | 2.8828613042 | -0.6541266552 | 3.6606071872 |
| C | 3.5142076175 | 0.4369147484 | 4.3177345064 |
| N | 3.4235834670 | -1.8133555119 | 4.1842882415 |
| C | 4.4541857144 | -0.1258494411 | 5.2493404532 |
| C | 4.3773930453 | -1.4959872830 | 5.1408530106 |
| H | 3.1840819107 | -2.7495049599 | 3.8983410186 |
| C | 7.4743834807 | -2.5016846680 | 6.8798384089 |
| C | 9.6151328028 | -2.3897976433 | 7.8703542126 |
| C | 10.2438011149 | -1.8291506649 | 9.1309812548 |
| H | 9.7273630917 | -3.4764664691 | 7.7988240368 |
| C | 1.9015489631 | -0.4726613085 | 2.6811568350 |
| C | 3.1407900940 | 1.7466271994 | 3.9704770555 |
| C | 1.5553378021 | 0.8348166133 | 2.3586311232 |
| H | 1.4282636027 | -1.3181552270 | 2.1894996213 |
| C | 2.1684776621 | 1.9329087372 | 2.9960321647 |
| H | 3.6078220521 | 2.5985217027 | 4.4569432456 |
| H | 0.7979539539 | 1.0126251549 | 1.6006521764 |
| H | 1.8726232824 | 2.9401674539 | 2.7178077297 |
| O | 7.9293532996 | -3.1165504639 | 5.9311590032 |
| O | 8.2031402350 | -2.0686521647 | 7.9152150746 |
| H | 10.0461954960 | -1.9530596288 | 6.9636358551 |
| H | 11.3183945880 | -2.0377355124 | 9.1329403863 |
| H | 10.1018813619 | -0.7461593702 | 9.1882742840 |
| H | 9.8015638959 | -2.2817036432 | 10.0228880027 |
| H | 5.7913348401 | -3.1137763698 | 5.1164539140 |
| H | 3.8773999697 | -1.2907497108 | 7.3243825334 |
| H | 5.1202788178 | 0.4150899103 | 5.9072029964 |

Indole

| C | 3.6553592035 | 0.6357297830 | -0.9335112328 |
| --- | --- | --- | --- |
| C | 3.4182419556 | -0.6697604981 | -0.5937176430 |
| N | 5.0135659919 | 0.8914484398 | -0.8699897421 |
| C | 4.6917169820 | -1.2687972207 | -0.3009725259 |
| C | 5.6765173959 | -0.2568628825 | -0.4850221360 |
| C | 5.1080023062 | -2.5517425617 | 0.0976715238 |
| C | 7.0406752096 | -0.4937564763 | -0.2827631746 |
| C | 6.4609514796 | -2.7911930299 | 0.2996536469 |
| H | 4.3785480637 | -3.3436866165 | 0.2453443305 |
| C | 7.4179955472 | -1.7722212254 | 0.1114322785 |
| H | 7.7786715751 | 0.2908014179 | -0.4274408488 |
| H | 6.7924071393 | -3.7788454137 | 0.6075299954 |
| H | 8.4692852859 | -1.9898444703 | 0.2773770066 |
| H | 2.4503655546 | -1.1494309427 | -0.5562900409 |
| H | 5.4490121879 | 1.7757614442 | -1.0740829950 |
| H | 2.9645841217 | 1.4165002529 | -1.2189184426 |

*Endo* cycloadduct **7a** – C3 alkylation

| C | -0.0508785462 | -0.1606625543 | 1.0146639023 |
| --- | --- | --- | --- |
| C | -2.0191062306 | 0.6502993074 | 2.0357198266 |
| C | -2.4013814201 | 1.6374208040 | 3.1211672411 |
| H | -2.4334866582 | 0.9245903455 | 1.0602751450 |
| C | 1.4472394133 | -0.1461308685 | 0.9794023166 |
| C | 2.1567604298 | -0.9994970909 | -0.0290359486 |
| N | 2.0792387645 | 0.5595002677 | 1.8524361548 |
| O | 3.4687676028 | 0.4620355035 | 1.7473978693 |
| C | 3.2685372487 | -0.1752388208 | -0.6995114075 |
| C | 4.0305704904 | 0.6238553930 | 0.3970162962 |
| N | 4.3048035332 | -0.9954784291 | -1.3623941357 |
| C | 5.4105170869 | 0.0425271846 | 0.3653917882 |
| C | 5.4730777979 | -0.8854593228 | -0.6017504380 |
| H | 6.2187445968 | 0.3640358462 | 1.0064852397 |
| H | 6.3300617386 | -1.4902566749 | -0.8790732771 |
| H | 4.0259891195 | -1.9349919354 | -1.6247587621 |
| O | -0.7051946968 | -0.8556889560 | 0.2581464260 |
| O | -0.5735427221 | 0.6491816820 | 1.9439333894 |
| H | -2.3580629901 | -0.3655898456 | 2.2636327743 |
| H | -1.9693591987 | 1.3463091126 | 4.0827355890 |
| H | -3.4906669995 | 1.6675868037 | 3.2259321717 |
| H | -2.0491519128 | 2.6433800106 | 2.8761939708 |
| H | 2.8246150936 | 0.5080748292 | -1.4305548389 |
| H | 2.6359820745 | -1.8252598675 | 0.5155296125 |
| H | 1.4462109205 | -1.4138681379 | -0.7454804743 |
| H | 3.9957154640 | 1.7026254138 | 0.2011995689 |

*Exo* cycloadduct **7a** – C3 alkylation

| C | 0.0940847767 | -0.3363517730 | 1.4980841120 |
| --- | --- | --- | --- |
| C | -1.6964888466 | 0.3790899041 | 2.8555293503 |
| C | -2.1186283989 | 1.6284444940 | 3.6030621778 |
| H | -2.3912369137 | 0.1213534875 | 2.0503728972 |
| C | 1.4487051603 | -0.0338285933 | 0.9274283565 |
| C | 2.0159829988 | -0.9956725989 | -0.0814724961 |
| N | 2.0173322012 | 1.0705603302 | 1.2644756116 |
| O | 3.2759379512 | 1.2587801046 | 0.6774007035 |
| C | 3.5402293302 | -1.1527083766 | 0.0682307884 |
| C | 4.1504407376 | 0.0876964541 | 0.7660133746 |
| N | 4.2481589164 | -1.2243668906 | -1.2279281244 |
| C | 5.4002663048 | 0.3497928412 | -0.0166150485 |
| C | 5.3913428135 | -0.4294140939 | -1.1101625621 |
| H | 6.1250098870 | 1.1126708441 | 0.2282189643 |
| H | 6.1363202718 | -0.4697728670 | -1.8977185964 |
| H | 4.3829324121 | -2.1552003809 | -1.6050076090 |
| O | -0.4904429526 | -1.3770499420 | 1.2547679148 |
| O | -0.3910494745 | 0.6345776703 | 2.2829408138 |
| H | -1.6240021160 | -0.4908727743 | 3.5171528669 |
| H | -1.3972290240 | 1.8787667200 | 4.3859740889 |
| H | -3.0947212353 | 1.4648047406 | 4.0713265485 |
| H | -2.2002110343 | 2.4824036581 | 2.9245479412 |
| H | 1.4801911703 | -1.9442105358 | -0.0319237898 |
| H | 1.8337446488 | -0.5599386406 | -1.0717193737 |
| H | 4.2987622420 | -0.0880832420 | 1.8390265917 |
| H | 3.7734681730 | -2.0400705399 | 0.6718944979 |

Open chain oxime **8a** – C3 alkylation

| C | -0.0668307380 | 0.5243534289 | -0.3937246524 |
| --- | --- | --- | --- |
| C | -1.8099038194 | 1.8832662335 | -1.2302958030 |
| C | -2.3452029783 | 3.2847879098 | -1.0111054523 |
| H | -1.4999359685 | 1.7167886114 | -2.2672416050 |
| C | 1.1224296186 | 0.4149980431 | 0.5149271183 |
| C | 1.6940114156 | -0.9789439016 | 0.7236431978 |
| N | 1.5398271275 | 1.5134150863 | 1.0475682741 |
| O | 2.6346159580 | 1.4522482585 | 1.8607771528 |
| C | 3.1723399771 | -1.1104808790 | 0.4780535879 |
| C | 4.1929153744 | -1.5542174591 | 1.3043105390 |
| N | 3.7453703047 | -0.7994313795 | -0.7359735272 |
| C | 5.4078338231 | -1.5060314764 | 0.5615060649 |
| C | 5.0994097970 | -1.0360288362 | -0.6953799611 |
| H | 6.3918007317 | -1.7849084058 | 0.9108140753 |
| H | 5.7247153714 | -0.8454889120 | -1.5548649885 |
| O | -0.4423714108 | -0.4074840619 | -1.0852764101 |
| O | -0.6583740219 | 1.7251808387 | -0.3664360065 |
| H | -2.5476358758 | 1.1144578977 | -0.9793898994 |
| H | -2.6379806134 | 3.4308547840 | 0.0325326148 |
| H | -3.2240690537 | 3.4491543789 | -1.6428879311 |
| H | -1.5912281074 | 4.0353657050 | -1.2644336985 |
| H | 1.4998570493 | -1.2815249673 | 1.7609423680 |
| H | 1.1239603208 | -1.6586260765 | 0.0832150021 |
| H | 3.0792422593 | 0.5798185900 | 1.7707290114 |
| H | 4.0679901392 | -1.8929349006 | 2.3244992206 |
| H | 3.2396133195 | -0.4435885099 | -1.5325082919 |

*Exo* cycloadduct **7a’** – C2 alkylation

| C | 0.1074348837 | 0.4812449318 | 0.1273428098 |
| --- | --- | --- | --- |
| C | -1.6029331459 | 1.9700341831 | -0.5199764851 |
| C | -1.7753682688 | 3.4556478711 | -0.7707490389 |
| H | -1.8098103346 | 1.3748264061 | -1.4153663176 |
| C | 1.5370828266 | 0.2903950495 | 0.5306458087 |
| C | 1.9647854616 | -1.1074529856 | 0.8713519396 |
| N | 2.2812414001 | 1.3389667820 | 0.5598769029 |
| O | 3.5965743573 | 1.2234089545 | 0.9383227174 |
| C | 4.2650004303 | -0.0708321187 | 1.1890086270 |
| C | 3.4727834172 | -1.2913357662 | 0.6879485308 |
| N | 5.4696960518 | -0.0827564700 | 0.3879611420 |
| C | 3.9964445700 | -1.4246878708 | -0.7310148557 |
| C | 5.1276575989 | -0.7171561600 | -0.8296923931 |
| H | 3.5730296902 | -2.0761162718 | -1.4836003903 |
| H | 5.8023789092 | -0.6345240363 | -1.6735147457 |
| H | 5.9177183991 | 0.8253265408 | 0.3147585093 |
| O | -0.6640890198 | -0.4582768581 | 0.0450035365 |
| O | -0.2285886458 | 1.7553832736 | -0.1221566937 |
| H | -2.2622820763 | 1.6066535335 | 0.2751179708 |
| H | -1.5734075296 | 4.0330588500 | 0.1357583018 |
| H | -2.8029488039 | 3.6591908752 | -1.0888126377 |
| H | -1.0961909664 | 3.7999811103 | -1.5558500622 |
| H | 4.4797516165 | -0.0741461150 | 2.2602555355 |
| H | 1.6783168064 | -1.3214396555 | 1.9102660337 |
| H | 1.4002372501 | -1.8125983244 | 0.2562620967 |
| H | 3.7961851220 | -2.1619957292 | 1.2775531575 |

*Endo* cycloadduct **7a’** – C2 alkylation

| C | -0.1241859380 | 0.7645948049 | -0.2931802676 |
| --- | --- | --- | --- |
| C | -2.2377746694 | 1.5942411359 | 0.3549294652 |
| C | -2.8163232091 | 2.4808400708 | 1.4408562030 |
| H | -2.4100133697 | 2.0010204759 | -0.6468600268 |
| C | 1.3455792325 | 0.6940854305 | -0.0131254188 |
| C | 2.1945655764 | -0.0558650607 | -0.9974042715 |
| N | 1.7639993262 | 1.2878833086 | 1.0482962189 |
| O | 3.1037496540 | 1.2684130838 | 1.3496572558 |
| C | 4.0985496721 | 0.4656931518 | 0.6071145108 |
| C | 3.4786230946 | -0.5731884376 | -0.3449179286 |
| N | 4.8073331238 | -0.3334863749 | 1.5831478247 |
| C | 3.3594438707 | -1.7808455490 | 0.5673497539 |
| C | 4.1358626269 | -1.5777169001 | 1.6376716618 |
| H | 2.8201821203 | -2.6858212799 | 0.3218166916 |
| H | 4.3426588382 | -2.2507584987 | 2.4613151874 |
| O | -0.6236070115 | 0.1932246489 | -1.2464788830 |
| O | -0.8125515726 | 1.5066861489 | 0.5864582867 |
| H | -2.6624426093 | 0.5851718264 | 0.3761679280 |
| H | -2.6241858108 | 2.0591040479 | 2.4316325198 |
| H | -3.8991804558 | 2.5720724613 | 1.3073729952 |
| H | -2.3773265331 | 3.4818742474 | 1.4024543253 |
| H | 1.6090873373 | -0.8740929375 | -1.4243080759 |
| H | 2.4346311717 | 0.6112834947 | -1.8369043347 |
| H | 4.2150347318 | -0.7854607162 | -1.1338868837 |
| H | 4.7469013152 | 1.1997596453 | 0.1231757389 |
| H | 4.9518894876 | 0.1417877718 | 2.4684495235 |

Open chain oxime **8a’** – C2 alkylation

| C | 0.0032098757 | 0.3281064428 | -0.2970744676 |
| --- | --- | --- | --- |
| C | -1.9957418472 | 1.5760666174 | -0.4157545881 |
| C | -2.4620105643 | 2.9135996184 | -0.9573479031 |
| H | -2.5061140362 | 0.7351644591 | -0.8963507322 |
| C | 1.4724224629 | 0.2618818769 | -0.5946752774 |
| C | 2.1842436654 | -1.0288621895 | -0.2366829305 |
| N | 1.9860134428 | 1.2913121098 | -1.1787897788 |
| C | 3.6492361634 | -0.0010030801 | 1.6463367438 |
| C | 3.4435608989 | -0.8706814456 | 0.5883761957 |
| N | 4.9071013461 | -0.2302615277 | 2.1446118387 |
| C | 4.6404645334 | -1.6460312949 | 0.4562147690 |
| C | 5.5223329828 | -1.2274391070 | 1.4275941979 |
| H | 4.8184771711 | -2.4272625881 | -0.2706833414 |
| H | 6.5251620962 | -1.5511880468 | 1.6628737487 |
| O | -0.5945315031 | -0.5866505528 | 0.2428987698 |
| O | -0.5766484223 | 1.4758342284 | -0.6795061807 |
| H | -2.1609423666 | 1.4868628751 | 0.6631099485 |
| H | -1.9353757387 | 3.7385725666 | -0.4690102639 |
| H | -3.5352153348 | 3.0322630680 | -0.7770063162 |
| H | -2.2826077667 | 2.9819836386 | -2.0339772158 |
| H | 1.4561551983 | -1.6654356770 | 0.2734860344 |
| H | 2.4420412274 | -1.5439569000 | -1.1729985306 |
| H | 5.3296391888 | 0.2859979925 | 2.8996780982 |
| H | 3.0054135481 | 0.7561045463 | 2.0689739976 |
| O | 3.3221612209 | 1.2473291320 | -1.4711897484 |
| H | 3.7419525578 | 0.5049932374 | -0.9848070678 |

Pyrrolo[3,2-*c*]carbazole **10**

| C | -0.6694641938 | -0.9541709895 | 1.3809107845 |
| --- | --- | --- | --- |
| C | -2.0581713037 | -1.2155345635 | 1.2239235776 |
| N | -0.1635622282 | -0.6586776672 | 0.1359244320 |
| C | -2.3517971010 | -1.0603344083 | -0.1754821246 |
| C | -1.1829805837 | -0.7234203267 | -0.8026454731 |
| H | -0.9811251700 | -0.5211133916 | -1.8445846408 |
| C | -0.0505944171 | -1.0192506831 | 2.6424086408 |
| C | -2.8418607131 | -1.5487722309 | 2.3517362014 |
| C | -0.8845654091 | -1.3568605425 | 3.7347587783 |
| C | -2.2621379830 | -1.6208813943 | 3.6092726593 |
| H | -3.9029877328 | -1.7493112222 | 2.2336547873 |
| H | -2.8566261538 | -1.8755646652 | 4.4818939020 |
| H | -3.3137340588 | -1.1839647652 | -0.6530958202 |
| N | -0.1047502948 | -1.3720524837 | 4.8853041407 |
| C | 1.2831204412 | -0.8236604574 | 3.1673689384 |
| C | 1.2030585168 | -1.0531935570 | 4.5699908157 |
| C | 2.3187850897 | -0.9534233286 | 5.4028094942 |
| C | 2.5278034922 | -0.4880287101 | 2.6185851992 |
| C | 3.5515335005 | -0.6130144073 | 4.8398479667 |
| C | 3.6393641529 | -0.3872423666 | 3.4496327517 |
| C | 4.7740671720 | -0.4584992116 | 5.7158885479 |
| H | 5.6946515855 | -0.6322632106 | 5.1501259820 |
| H | 4.8384757669 | 0.5531051978 | 6.1375668549 |
| H | 4.7582991032 | -1.1593629690 | 6.5567922165 |
| H | 2.2334990453 | -1.1365124844 | 6.4714596774 |
| H | 4.6038013177 | -0.1281358502 | 3.0206230512 |
| H | 2.6393489036 | -0.3096487807 | 1.5519021982 |
| H | 0.7952645081 | -0.4296143710 | -0.0647661561 |
| H | -0.4440152528 | -1.5821961593 | 5.8093926168 |

*Endo* cycloadduct **16** – C2 alkylation

| C | -0.0160058093 | 0.6927842191 | 0.0794028132 |
| --- | --- | --- | --- |
| C | 1.8034194258 | 2.0867011989 | -0.4901722810 |
| C | 2.0238408788 | 3.4680659399 | -1.0751141720 |
| H | 2.2637095378 | 1.3011924695 | -1.0984220239 |
| C | -1.5061519047 | 0.5114241844 | 0.0985841498 |
| C | -2.0627661949 | -0.7842907635 | 0.6203991340 |
| H | -2.3534268460 | -1.3837566420 | -0.2514463632 |
| H | -1.2930620456 | -1.3339179005 | 1.1634676829 |
| N | -2.2363944360 | 1.4413346540 | -0.4093849354 |
| O | -3.6122063759 | 1.1642827187 | -0.3454920148 |
| C | -3.3414591894 | -0.5571974656 | 1.4482025670 |
| C | -4.0587494611 | 0.7340466160 | 0.9769221835 |
| N | -4.3372029546 | -1.6341755821 | 1.2653532215 |
| C | -5.5014178389 | 0.3374630877 | 0.9098597851 |
| C | -5.5934756914 | -1.0474581975 | 1.0993700457 |
| C | -6.6364078288 | 1.1239263856 | 0.6898388086 |
| C | -6.8460501973 | -1.6730413845 | 1.0786099781 |
| C | -7.9035353141 | 0.5411000719 | 0.6782509529 |
| H | -6.5303062110 | 2.1935912901 | 0.5352307139 |
| C | -7.9846428680 | -0.8434879504 | 0.8760052379 |
| H | -8.7949772586 | 1.1395550874 | 0.5193650162 |
| H | -4.2816293464 | -2.4037740682 | 1.9209102059 |
| O | 0.7433600100 | -0.1557954418 | 0.5103388210 |
| O | 0.3734554425 | 1.8622151702 | -0.4441858890 |
| H | 2.2067756140 | 1.9969116816 | 0.5235282658 |
| H | 1.5486071221 | 4.2340203149 | -0.4556090783 |
| H | 3.0964145027 | 3.6814566453 | -1.1265966063 |
| H | 1.6082697011 | 3.5361995074 | -2.0843627860 |
| N | -9.1093055043 | -1.6516942078 | 0.9117239060 |
| C | -7.3247202252 | -3.0320875006 | 1.2186168300 |
| C | -8.7389580187 | -2.9731673006 | 1.1123613434 |
| H | -10.0540143496 | -1.3290257503 | 0.7799038807 |
| C | -9.5381290512 | -4.1146246061 | 1.2006117853 |
| C | -6.7180261181 | -4.2822975721 | 1.4005407778 |
| C | -7.5136067046 | -5.4199001673 | 1.4862491409 |
| C | -8.9209765319 | -5.3528582829 | 1.3926773920 |
| C | -9.7446697500 | -6.6156641761 | 1.5010849276 |
| H | -9.6331732219 | -7.0803842848 | 2.4882960549 |
| H | -9.4332828835 | -7.3607365621 | 0.7596039418 |
| H | -10.8089866195 | -6.4158364564 | 1.3467830708 |
| H | -10.6199607676 | -4.0466634176 | 1.1170821183 |
| H | -7.0428571692 | -6.3901633082 | 1.6230867601 |
| H | -5.6365349087 | -4.3715485273 | 1.4537728982 |
| H | -3.0857230641 | -0.4490433442 | 2.5109918647 |
| H | -3.8474595747 | 1.5725196178 | 1.6518598748 |

*Exo* cycloadduct **16** – C2 alkylation

| C | 0.0175347245 | 0.6946947413 | 0.0825571503 |
| --- | --- | --- | --- |
| C | -1.8025805034 | 2.0820543371 | -0.4994507039 |
| C | -2.0246801637 | 3.4608304848 | -1.0900607133 |
| H | -2.2583143860 | 1.2931676052 | -1.1068547488 |
| C | 1.5077719685 | 0.5150879902 | 0.1073498471 |
| C | 2.0624561089 | -0.7797698571 | 0.6333981275 |
| H | 2.3489815983 | -1.3837838125 | -0.2366633903 |
| H | 1.2921116911 | -1.3249523198 | 1.1800859487 |
| N | 2.2385210566 | 1.4442829114 | -0.4011992797 |
| O | 3.6143511947 | 1.1692553788 | -0.3347185375 |
| C | 3.3433088057 | -0.5537376585 | 1.4581164107 |
| C | 4.0608171026 | 0.7374730435 | 0.9872673082 |
| N | 4.3379714289 | -1.6311521319 | 1.2714036286 |
| C | 5.5032667528 | 0.3402173484 | 0.9186693964 |
| C | 5.5944927246 | -1.0451387483 | 1.1052586803 |
| C | 6.6385644429 | 1.1260723036 | 0.6980971029 |
| C | 6.8464342045 | -1.6718332861 | 1.0810923845 |
| C | 7.9051059754 | 0.5420359617 | 0.6822992604 |
| H | 6.5332454642 | 2.1962127858 | 0.5463788216 |
| C | 7.9852698609 | -0.8430781950 | 0.8766415987 |
| H | 8.7967471819 | 1.1399898778 | 0.5226194992 |
| H | 4.2823012218 | -2.4018041738 | 1.9257379092 |
| O | -0.7419234879 | -0.1536346728 | 0.5137519859 |
| O | -0.3723006925 | 1.8614681827 | -0.4466807907 |
| H | -2.2099411236 | 1.9942572698 | 0.5127756575 |
| H | -1.5544334866 | 4.2304568535 | -0.4712176188 |
| H | -3.0978362160 | 3.6704674822 | -1.1468271937 |
| H | -1.6055123728 | 3.5265685955 | -2.0980461820 |
| N | 9.1091834226 | -1.6525221524 | 0.9071997006 |
| C | 7.3241838493 | -3.0315058388 | 1.2181826679 |
| C | 8.7381366798 | -2.9738890328 | 1.1073354992 |
| H | 10.0538690731 | -1.3307047405 | 0.7731696330 |
| C | 9.5363637860 | -4.1162933798 | 1.1916570607 |
| C | 6.7168817419 | -4.2811815719 | 1.4017121435 |
| C | 7.5116132402 | -5.4196600958 | 1.4841050562 |
| C | 8.9186835997 | -5.3540283898 | 1.3854329340 |
| C | 9.7416493186 | -6.6175109089 | 1.4917480337 |
| H | 9.6452373247 | -7.0733507985 | 2.4847188018 |
| H | 9.4168517027 | -7.3685210223 | 0.7622919280 |
| H | 10.8036571743 | -6.4210626380 | 1.3188038378 |
| H | 10.6179379228 | -4.0494305765 | 1.1039312786 |
| H | 7.0403496909 | -6.3894793070 | 1.6224652342 |
| H | 5.6355467496 | -4.3695154923 | 1.4591175912 |
| H | 3.0904480219 | -0.4465010717 | 2.5216689619 |
| H | 3.8500756257 | 1.5756487196 | 1.6627780787 |

Open chain oxime **12a** – C2 alkylation

| C | 1.0532987760 | 2.4035259631 | -2.0325391981 |
| --- | --- | --- | --- |
| C | -0.1467517819 | 4.2699606025 | -2.8321567826 |
| C | -0.6234910993 | 4.8207623958 | -4.1618438613 |
| H | -0.9790903736 | 4.0279234439 | -2.1636888597 |
| C | 1.7973546530 | 1.1416967633 | -2.3626528155 |
| C | 2.3538364715 | 0.3643443840 | -1.1875660896 |
| H | 1.8501365433 | -0.6157544468 | -1.1676763157 |
| H | 2.0456435997 | 0.8953340898 | -0.2811485069 |
| N | 1.8219818833 | 0.7898266276 | -3.6021722217 |
| C | 3.8452666104 | 0.1574326547 | -1.1957781972 |
| C | 4.8356635845 | 0.6314214255 | -2.0243801973 |
| N | 4.4359463614 | -0.6229746648 | -0.2074631608 |
| C | 6.0933965948 | 0.1351850533 | -1.5334259231 |
| C | 5.8024228414 | -0.6466796021 | -0.3852944882 |
| C | 7.4298171979 | 0.2859028194 | -1.9656354450 |
| C | 6.8214197846 | -1.2814802821 | 0.3450972633 |
| C | 8.4593852114 | -0.3247231172 | -1.2668355397 |
| H | 7.6505249558 | 0.8819733422 | -2.8463526519 |
| C | 8.1426569844 | -1.0929415848 | -0.1295109613 |
| H | 9.4909621039 | -0.2146327675 | -1.5881853354 |
| H | 3.9377973181 | -1.0643339103 | 0.5482643688 |
| O | 0.8871938265 | 2.7756480133 | -0.8840460896 |
| O | 0.5993477703 | 3.0598897045 | -3.1076206257 |
| H | 0.5058623003 | 4.9722303386 | -2.3029070635 |
| H | 0.2203852417 | 5.0617154978 | -4.8144402635 |
| H | -1.2033064761 | 5.7343185569 | -3.9955680782 |
| H | -1.2605264141 | 4.0962828661 | -4.6769924700 |
| N | 9.0042517585 | -1.7837177820 | 0.7117962948 |
| C | 6.9038001655 | -2.1230340588 | 1.5184456875 |
| C | 8.2823327762 | -2.4122430541 | 1.7103785192 |
| H | 10.0044160673 | -1.8259091650 | 0.6052262635 |
| C | 8.7338913198 | -3.2124322143 | 2.7630534610 |
| C | 5.9773291604 | -2.6650985338 | 2.4217540396 |
| C | 6.4267281214 | -3.4620853772 | 3.4673583334 |
| C | 7.7991376033 | -3.7445033695 | 3.6523068839 |
| C | 8.2401958435 | -4.6130951327 | 4.8078873726 |
| H | 7.9816528422 | -4.1579209854 | 5.7715975834 |
| H | 7.7534375047 | -5.5951572738 | 4.7775051663 |
| H | 9.3216187290 | -4.7765959175 | 4.7969469289 |
| H | 9.7936740991 | -3.4199967615 | 2.8893659092 |
| H | 5.7067287083 | -3.8817886569 | 4.1653863394 |
| H | 4.9126402284 | -2.4722102582 | 2.3147753822 |
| H | 4.6842573774 | 1.2800415254 | -2.8760796077 |
| O | 2.4885195051 | -0.3639945724 | -3.9187595125 |
| H | 3.0903537210 | -0.6126125789 | -3.1848255355 |

*Exo* cycloadduct **15** – C3 alkylation

| C | 0.4308261239 | 0.4268322532 | -0.3792873537 |
| --- | --- | --- | --- |
| C | -1.0370532386 | -0.7294474034 | -1.8190501818 |
| C | -1.1664456522 | -1.0234587719 | -3.3011391737 |
| H | -0.8915885007 | -1.6392187670 | -1.2284427939 |
| C | 1.6299958230 | 1.3123069793 | -0.2387157454 |
| C | 2.0459056374 | 1.6933830715 | 1.1527659167 |
| H | 1.4965415547 | 2.5961833563 | 1.4542858173 |
| N | 2.2107054404 | 1.6901891297 | -1.3219798822 |
| O | 3.3016768808 | 2.5249650164 | -1.2384975812 |
| C | 4.0264284756 | 2.7985725211 | 0.0202381898 |
| C | 3.5518359640 | 1.9497454120 | 1.2157748853 |
| N | 5.4058370907 | 2.4107210728 | -0.1724767868 |
| C | 4.4682850661 | 0.7422802922 | 1.1165449941 |
| C | 5.5426075518 | 1.0877748270 | 0.2900198979 |
| C | 4.4020125056 | -0.5098977801 | 1.7292078972 |
| C | 6.5814355517 | 0.1827901355 | 0.0600120408 |
| C | 5.4088784376 | -1.4552051552 | 1.5116931246 |
| H | 3.5610238979 | -0.7636493582 | 2.3691672544 |
| C | 6.4765420917 | -1.0938155900 | 0.6840954532 |
| H | 5.3606248256 | -2.4374384188 | 1.9711002803 |
| H | 5.7699772630 | 2.6160596672 | -1.0963048669 |
| O | -0.1871559581 | 0.0264827338 | 0.5917800592 |
| O | 0.1151815284 | 0.1289698612 | -1.6478991012 |
| H | -1.9198872909 | -0.2172450368 | -1.4214469018 |
| H | -1.3039623231 | -0.1019690715 | -3.8738788827 |
| H | -2.0318373327 | -1.6711772686 | -3.4750295267 |
| H | -0.2733994902 | -1.5313362890 | -3.6765198251 |
| N | 7.5856180621 | -1.8396690536 | 0.3132163530 |
| C | 7.8126356500 | 0.1864616214 | -0.7022234086 |
| C | 8.4024097208 | -1.0923267283 | -0.5213576763 |
| H | 7.7649996565 | -2.7873176468 | 0.6019460924 |
| C | 9.6134539713 | -1.4410852383 | -1.1224658480 |
| C | 8.4804053585 | 1.1261615846 | -1.4982824650 |
| C | 9.6865843884 | 0.7791762908 | -2.0972664463 |
| C | 10.2622109446 | -0.4982901615 | -1.9235491525 |
| H | 10.2048228258 | 1.5094453048 | -2.7134860664 |
| H | 8.0743646177 | 2.1241583873 | -1.6384626541 |
| H | 10.0483309572 | -2.4261104348 | -0.9710550855 |
| C | 11.5631295796 | -0.8411602099 | -2.6126936075 |
| H | 11.9900724510 | -1.7702416307 | -2.2239145004 |
| H | 11.4200473360 | -0.9696353757 | -3.6930105065 |
| H | 12.3072513477 | -0.0480133757 | -2.4799820988 |
| H | 3.9240173043 | 3.8767460782 | 0.1633937095 |
| H | 1.7398932434 | 0.9080371338 | 1.8472691500 |
| H | 3.7827606615 | 2.5112660355 | 2.1315070035 |

*Exo* cycloadduct **15** – C3 alkylation

| C | 0.7694915694 | 1.2807572869 | -1.8583033569 |
| --- | --- | --- | --- |
| C | -0.6224690675 | 2.7115486141 | -3.1189932668 |
| C | -0.6511906840 | 3.3185127237 | -4.5086358169 |
| H | -1.4796122914 | 2.0557366774 | -2.9354897594 |
| C | 2.0465032248 | 0.4992743554 | -1.7841497177 |
| C | 2.2260743699 | -0.3626173897 | -0.5663356241 |
| H | 1.8720830386 | 0.1898227893 | 0.3084321566 |
| N | 2.8713691300 | 0.6451564056 | -2.7600922712 |
| O | 4.0627770010 | -0.0402733817 | -2.7447017830 |
| C | 4.3646249279 | -1.0735843852 | -1.7396598552 |
| C | 3.6729511929 | -0.8220784820 | -0.3900260252 |
| N | 5.7721031056 | -0.9550961072 | -1.4444034488 |
| C | 4.6641150471 | 0.1220963398 | 0.2713640085 |
| C | 5.8901602525 | -0.0290974079 | -0.3870185297 |
| C | 4.5397323640 | 0.9596288243 | 1.3803213934 |
| C | 7.0241702867 | 0.6547366935 | 0.0551556060 |
| C | 5.6432331810 | 1.6824973630 | 1.8445572580 |
| H | 3.5821081223 | 1.0654186238 | 1.8832121684 |
| C | 6.8611188648 | 1.5163094459 | 1.1785257208 |
| H | 5.5549127496 | 2.3503282370 | 2.6957184481 |
| H | 6.3557801914 | -0.7937330288 | -2.2581967380 |
| O | -0.0252176632 | 1.2933971912 | -0.9349507110 |
| O | 0.5932404368 | 1.9333831410 | -3.0157839907 |
| H | -0.6189517315 | 3.4762719130 | -2.3352766808 |
| H | 0.2180275697 | 3.9621588823 | -4.6705634929 |
| H | -1.5566047904 | 3.9215285693 | -4.6316930069 |
| H | -0.6480697439 | 2.5379991947 | -5.2749398879 |
| N | 8.0879668377 | 2.1076406537 | 1.4410226634 |
| C | 8.4120874117 | 0.7386389508 | -0.3505911796 |
| C | 9.0361349478 | 1.6546574708 | 0.5368012640 |
| H | 8.2627242369 | 2.7671155428 | 2.1815484210 |
| C | 10.3914866064 | 1.9757605993 | 0.4388386153 |
| C | 9.1913286578 | 0.1338805719 | -1.3453992436 |
| C | 10.5413869074 | 0.4539599097 | -1.4436610150 |
| C | 11.1538320712 | 1.3731449986 | -0.5646405319 |
| H | 11.1453208535 | -0.0199247430 | -2.2132509908 |
| H | 8.7583764940 | -0.5921086177 | -2.0281246132 |
| H | 10.8487216493 | 2.6790468843 | 1.1306459848 |
| C | 12.6187686187 | 1.7092667802 | -0.7236660642 |
| H | 13.0032624658 | 2.2504286074 | 0.1457930793 |
| H | 13.2249165660 | 0.8060069287 | -0.8529243650 |
| H | 12.7889533954 | 2.3388018793 | -1.6062354045 |
| H | 1.5557504880 | -1.2284598773 | -0.6502843364 |
| H | 4.1184988458 | -2.0326587146 | -2.2043101708 |
| H | 3.6767222923 | -1.7757809138 | 0.1569650902 |

Open chain oxime **11a** – C3 alkylation

| C | 0.2529914323 | 0.0090903623 | 0.8366764402 |
| --- | --- | --- | --- |
| C | -1.1457363589 | 0.0845322739 | 2.7377408219 |
| C | -2.0773990796 | 1.0730864449 | 3.4116313181 |
| H | -1.6710221623 | -0.8121826556 | 2.3944751594 |
| C | 0.8913291452 | 0.7617853346 | -0.2921421514 |
| C | 1.5844033781 | -0.0600819139 | -1.3682096481 |
| H | 1.4168130604 | -1.1121223592 | -1.1221326422 |
| N | 0.7840153878 | 2.0460445619 | -0.2502557220 |
| C | 3.6844012979 | 0.6948295789 | -2.6628367213 |
| C | 3.0614082541 | 0.2051330149 | -1.5367692666 |
| N | 5.0488076933 | 0.7716841780 | -2.4395528105 |
| C | 4.0986753101 | -0.0417765598 | -0.5633867587 |
| C | 5.3284469783 | 0.3290146694 | -1.1657647452 |
| C | 4.0772988275 | -0.5669156362 | 0.7494035050 |
| C | 6.5461674783 | 0.2047735198 | -0.4732822257 |
| C | 5.2612834113 | -0.7078701076 | 1.4545450800 |
| H | 3.1354435165 | -0.8720717349 | 1.1947990827 |
| C | 6.4683058425 | -0.3185420447 | 0.8398344177 |
| H | 5.2616471880 | -1.1145167497 | 2.4614812965 |
| H | 5.7253223241 | 1.1073823786 | -3.1044454829 |
| O | 0.4746408401 | -1.1742248232 | 1.0373671069 |
| O | -0.5607771420 | 0.7543836080 | 1.5959717774 |
| H | -0.3397576936 | -0.2407612465 | 3.4042200298 |
| H | -1.5355866772 | 1.9728640086 | 3.7160246241 |
| H | -2.5192959233 | 0.6154264446 | 4.3023483450 |
| H | -2.8857184634 | 1.3700591051 | 2.7373741081 |
| N | 7.7546332332 | -0.3704350513 | 1.3584689656 |
| C | 7.9399481060 | 0.4758178451 | -0.7450869716 |
| C | 8.6579563115 | 0.1034608051 | 0.4241094753 |
| H | 7.9908916381 | -0.6989070068 | 2.2803769562 |
| C | 10.0462986888 | 0.2307597713 | 0.5150632297 |
| C | 8.6600440150 | 0.9852974767 | -1.8352651215 |
| C | 10.0410613578 | 1.1117864100 | -1.7443637971 |
| C | 10.7476807698 | 0.7418096902 | -0.5781495721 |
| H | 10.5968647064 | 1.5049516213 | -2.5917944708 |
| H | 8.1544933330 | 1.2808029846 | -2.7512977995 |
| H | 10.5739157048 | -0.0617184350 | 1.4195786021 |
| C | 12.2471319781 | 0.9213191372 | -0.5156131997 |
| H | 12.6784549378 | 0.4002434707 | 0.3439923054 |
| H | 12.7339734476 | 0.5389492196 | -1.4196107037 |
| H | 12.5180467657 | 1.9810911629 | -0.4286457203 |
| H | 3.2658596015 | 0.9679708702 | -3.6221397115 |
| H | 1.0739516593 | 0.1372871366 | -2.3209775041 |
| O | 1.4129806885 | 2.7555158989 | -1.2410972063 |
| H | 2.0774051913 | 2.1823733405 | -1.6784626944 |

TS*endo*[**2a** + pyrrole] – C2 alkylation

Imaginary frequency = -316 cm^–1^

| C | 0.8003628503 | 1.1868801714 | 0.2093400473 |
| --- | --- | --- | --- |
| C | -0.5278161799 | 1.1408986023 | -0.4143519660 |
| C | -0.7502777154 | 0.2590473916 | -1.4777435951 |
| H | 0.1176713685 | -0.1189641820 | -2.0067644187 |
| H | -1.6487050848 | 0.4391186446 | -2.0576067292 |
| N | -1.5503783512 | 1.9351641455 | 0.1171395056 |
| O | -2.6645688518 | 1.7621492842 | -0.4107592882 |
| H | -0.3920824552 | -1.1229397933 | 2.3635958291 |
| C | -0.9597924373 | -1.2700209719 | 1.4553127399 |
| C | -2.3286323665 | -1.1019118295 | 1.2244796851 |
| N | -0.3581287419 | -1.7052890919 | 0.3223996868 |
| C | -2.5439918954 | -1.3434012928 | -0.1289024517 |
| H | -3.0594514072 | -0.7999315404 | 1.9604537639 |
| C | -1.2789244545 | -1.5889520421 | -0.7315698023 |
| H | 0.6355957272 | -1.5375192631 | 0.1738615528 |
| H | -1.1094541538 | -2.1535597752 | -1.6400118084 |
| H | -3.4835281320 | -1.2876823324 | -0.6597170015 |
| C | 2.3005259160 | 2.3328278123 | 1.6348504479 |
| H | 2.4304533644 | 1.4494910960 | 2.2700994091 |
| H | 3.0840741975 | 2.3009147224 | 0.8706531809 |
| H | 3.3016931793 | 3.7145035334 | 2.9437756578 |
| C | 2.3356859792 | 3.6194542027 | 2.4371449783 |
| H | 1.5456051368 | 3.6304386371 | 3.1934851150 |
| H | 2.1988357260 | 4.4880802422 | 1.7869461364 |
| O | 1.6591020024 | 0.3177338617 | 0.0343378326 |
| O | 1.0110267794 | 2.2627697672 | 0.9874514925 |

TS*exo*[**2a** + pyrrole] – C2 alkylation

Imaginary frequency = -335 cm^–1^

| C | -1.6126075061 | 1.0323097951 | -0.0107513254 |
| --- | --- | --- | --- |
| C | -0.2246094339 | 1.2785702792 | 0.4159800309 |
| C | 0.4977235073 | 0.2134056251 | 0.9786233368 |
| H | -0.0918995011 | -0.6152935998 | 1.3556476481 |
| H | 1.4057244370 | 0.4744396892 | 1.5122100859 |
| N | 0.3828019206 | 2.4694453964 | 0.0605278058 |
| O | 1.6168417245 | 2.5062717466 | 0.2923108745 |
| H | 0.4642169723 | -1.0517272398 | -0.9930773389 |
| C | 1.3942612097 | -0.8326769338 | -0.4823756667 |
| C | 2.4251998008 | 0.0401994559 | -0.9438401008 |
| N | 2.0663951000 | -1.8972678982 | 0.1408741977 |
| C | 3.6506728177 | -0.4461016577 | -0.4775379311 |
| H | 2.2590151874 | 0.9460301128 | -1.5051964470 |
| C | 3.3936617542 | -1.6344524572 | 0.2043265575 |
| H | 1.6078894949 | -2.6755685111 | 0.5891423537 |
| H | 4.0699076671 | -2.2882716759 | 0.7379007844 |
| H | 4.6205285894 | 0.0155578023 | -0.5924997059 |
| C | -3.6444276204 | 1.9503864029 | -0.7971499767 |
| H | -3.6505830896 | 1.2839678168 | -1.6665694344 |
| H | -4.2061662232 | 1.4511364777 | -0.0003117948 |
| H | -5.2531711995 | 3.2157080081 | -1.4613597379 |
| C | -4.2162309252 | 3.3171994112 | -1.1245121821 |
| H | -3.6395860218 | 3.8012725887 | -1.9180972889 |
| H | -4.2000896369 | 3.9671636325 | -0.2448118501 |
| O | -2.1088262320 | -0.0887844081 | -0.0441823803 |
| O | -2.2827427934 | 2.1474801412 | -0.3620705142 |

TS*endo*[**2a** + pyrrole] – C3 alkylation

Imaginary frequency = -352 cm^–1^

| C | -0.4750325086 | 1.6877372111 | -0.7463373652 |
| --- | --- | --- | --- |
| C | -0.5217046374 | 0.5683386855 | -1.6092208784 |
| H | 0.4338708662 | 0.1343256525 | -1.8817851992 |
| H | -1.2814595033 | 0.6166702195 | -2.3828531918 |
| N | -1.6053183515 | 2.3944468740 | -0.4361234030 |
| O | -2.6596085053 | 1.9439142485 | -0.9754667513 |
| C | -1.0690402367 | -0.7257527031 | 1.5002397403 |
| C | -0.4407371670 | -1.2721394632 | 0.4303222635 |
| N | -2.3342032221 | -0.2842235951 | 1.0879024131 |
| C | -1.2915039696 | -1.0427112985 | -0.7348579594 |
| C | -2.5145837124 | -0.5096184604 | -0.2211491390 |
| H | -2.9764682050 | 0.2421526588 | 1.6627362087 |
| H | -3.4420194504 | -0.2890933350 | -0.7208057351 |
| C | 0.7851786168 | 1.9962721092 | -0.0481523729 |
| C | 1.9560133413 | 3.4839457788 | 1.3668374291 |
| C | 1.7085477711 | 4.7987486754 | 2.0831738617 |
| H | 2.2202154470 | 2.6804528175 | 2.0637705686 |
| H | -1.2749555447 | -1.6694680199 | -1.6187434511 |
| O | 1.7846073965 | 1.2936460688 | -0.1219234559 |
| O | 0.7372200528 | 3.1334769416 | 0.6811950175 |
| H | 2.7702928209 | 3.5610033060 | 0.6385003057 |
| H | 2.6123543276 | 5.1102821521 | 2.6170372218 |
| H | 1.4397044575 | 5.5839242900 | 1.3703319184 |
| H | 0.8941641578 | 4.7025127415 | 2.8073290257 |
| H | -0.7543986814 | -0.5955034258 | 2.5247205710 |
| H | 0.5623644399 | -1.6712401299 | 0.4126223573 |

TS*exo*[**2a** + pyrrole] – C3 alkylation

Imaginary frequency = -364 cm^–1^

| C | -0.1549724743 | 0.8160115941 | 0.4246565449 |
| --- | --- | --- | --- |
| C | -2.3209745892 | 1.6543124605 | -0.0034870389 |
| C | -3.0287442829 | 2.9950972690 | -0.0616263662 |
| H | -2.3692380800 | 1.1222949540 | -0.9599269115 |
| C | 1.2520435086 | 1.1198872293 | 0.7485696813 |
| C | 2.1326525505 | 0.0506852801 | 1.0198543125 |
| H | 1.6761983800 | -0.9138428591 | 1.2114773821 |
| H | 3.0212727030 | 0.3023729263 | 1.5874704417 |
| N | 1.7154545279 | 2.3981448717 | 0.5825726814 |
| O | 2.9744550146 | 2.5027128770 | 0.6812554308 |
| C | 3.8629893214 | 0.7044361442 | -0.9383738663 |
| C | 3.1042986241 | -0.4711518065 | -0.6546239118 |
| N | 5.1442671092 | 0.4592882571 | -0.6073413693 |
| C | 4.0829012806 | -1.4673789059 | -0.2298510988 |
| C | 5.2926162741 | -0.8537159138 | -0.1510847066 |
| H | 2.1967751093 | -0.7182525387 | -1.1913188735 |
| H | 5.8697442600 | 1.1608367114 | -0.5947839541 |
| H | 3.5579568815 | 1.6443324490 | -1.3646958287 |
| O | -0.5708653731 | -0.3239272299 | 0.2627943482 |
| O | -0.9388339409 | 1.9093682697 | 0.3233358240 |
| H | -2.7533908153 | 0.9949343130 | 0.7566030516 |
| H | -2.9747926314 | 3.5070021441 | 0.9036994720 |
| H | -4.0833587434 | 2.8481038068 | -0.3165647186 |
| H | -2.5762937215 | 3.6430473038 | -0.8180715282 |
| H | 3.8653831944 | -2.4885488332 | 0.0484343693 |
| H | 6.2555559128 | -1.2124507741 | 0.1809266329 |

TS*endo*[**2a** + indole] – C3 alkylation

Imaginary frequency = -330 cm^–1^

| C | -0.4442680849 | 1.6535512729 | -0.7475474437 |
| --- | --- | --- | --- |
| C | -0.5228086269 | 0.5693638013 | -1.6370186442 |
| H | 0.4083439262 | 0.0823395549 | -1.9030180085 |
| H | -1.3014815955 | 0.6281975210 | -2.3892060660 |
| N | -1.5586975387 | 2.3865333779 | -0.4159738059 |
| O | -2.6236364950 | 1.9781277650 | -0.9612779524 |
| C | -1.1670077672 | -0.7977822895 | 1.5261972538 |
| C | -0.5497543973 | -1.4005767825 | 0.4100353245 |
| N | -2.3868370840 | -0.2657045248 | 1.0885246704 |
| C | -1.3921262348 | -1.1020491116 | -0.7507001386 |
| C | -2.5628223720 | -0.4956056237 | -0.2284236808 |
| H | -3.0009445192 | 0.3104565579 | 1.6457657164 |
| H | -3.4735778539 | -0.2179027349 | -0.7311956289 |
| C | 0.8401641562 | 1.9446232270 | -0.0763957056 |
| C | 2.0302156488 | 3.3622552178 | 1.3893284468 |
| C | 1.7670145989 | 4.5727025628 | 2.2650313229 |
| H | 2.3902786355 | 2.5056362792 | 1.9687708798 |
| C | -0.6068461974 | -0.8149459074 | 2.8021124390 |
| C | 0.6744492753 | -2.0535193767 | 0.5793681110 |
| C | 0.6101435411 | -1.4786208352 | 2.9472440382 |
| H | -1.0953732704 | -0.3378487782 | 3.6464598321 |
| C | 1.2396582764 | -2.0949489848 | 1.8522319043 |
| H | 1.1783424893 | -2.5166019079 | -0.2639542757 |
| H | 1.0789864303 | -1.5206930613 | 3.9257948147 |
| H | 2.1871181882 | -2.6036228108 | 2.0013775465 |
| H | -1.4030064818 | -1.6908561104 | -1.6593253767 |
| O | 1.8586213299 | 1.2957829792 | -0.2610737396 |
| O | 0.7849058086 | 3.0109901714 | 0.7513411640 |
| H | 2.7800245132 | 3.5700326014 | 0.6184139669 |
| H | 2.6929153550 | 4.8810333211 | 2.7616263629 |
| H | 1.3974894622 | 5.4122439432 | 1.6693410086 |
| H | 1.0227168842 | 4.3455086859 | 3.0340456641 |

TS*exo*[**2a** + indole] – C3 alkylation

Imaginary frequency = -337 cm^–1^

| C | -0.0843082839 | 0.7061605735 | 0.4336906292 |
| --- | --- | --- | --- |
| C | -2.3379136148 | 1.3265567366 | 0.0818041594 |
| C | -3.1495370334 | 2.5979008089 | -0.0802681029 |
| H | -2.3808732687 | 0.6936967718 | -0.8106476356 |
| C | 1.3005713205 | 1.1596420488 | 0.6808741281 |
| C | 2.2952463415 | 0.2024229761 | 0.9255705235 |
| H | 1.9727493891 | -0.8153444349 | 1.1105567380 |
| H | 3.1919618996 | 0.5483062382 | 1.4258577349 |
| N | 1.6104483836 | 2.4853245557 | 0.4874333866 |
| O | 2.8485006010 | 2.7346533629 | 0.5264943814 |
| C | 3.9336465107 | 0.9997089039 | -1.1025480151 |
| C | 3.3030045972 | -0.2429652175 | -0.8477567225 |
| N | 5.2395452973 | 0.9201079646 | -0.7588788489 |
| C | 4.3765814577 | -1.1458127395 | -0.4314794845 |
| C | 5.5537762066 | -0.3711433515 | -0.3244611613 |
| C | 4.4209979559 | -2.5011397513 | -0.0877468959 |
| C | 6.7653795786 | -0.8961502433 | 0.1222771375 |
| C | 5.6295485323 | -3.0430426219 | 0.3465902437 |
| H | 3.5316732828 | -3.1205805923 | -0.1614793659 |
| C | 6.7848306102 | -2.2495078558 | 0.4551837611 |
| H | 7.6571753695 | -0.2817668747 | 0.2041692292 |
| H | 5.6818344587 | -4.0958406368 | 0.6070234685 |
| H | 7.7106554595 | -2.6982610761 | 0.8021747699 |
| H | 2.4015799153 | -0.5612776175 | -1.3538556569 |
| H | 5.8654067837 | 1.7112637227 | -0.7287121915 |
| H | 3.5241955774 | 1.9052513807 | -1.5178588916 |
| O | -0.3973483588 | -0.4728014619 | 0.3448568043 |
| O | -0.9689249496 | 1.7176479077 | 0.3178069845 |
| H | -2.6874382139 | 0.7225426929 | 0.9261255704 |
| H | -3.0855631072 | 3.2189430906 | 0.8179031322 |
| H | -4.2015184245 | 2.3492470767 | -0.2548299101 |
| H | -2.7876842740 | 3.1859576627 | -0.9285698996 |

TS*endo*[**2a** + indole] – C2 alkylation

Imaginary frequency = -357 cm^–1^

| C | -0.7109320829 | 1.5990041347 | -0.5438200847 |
| --- | --- | --- | --- |
| C | -0.7896729506 | 0.7030215926 | -1.6192109539 |
| H | 0.1227940699 | 0.5175648245 | -2.1760555092 |
| H | -1.7181893537 | 0.7262879574 | -2.1798536741 |
| N | -1.8573975300 | 2.0837497634 | 0.0775451858 |
| O | -2.9205225610 | 1.6270465796 | -0.3991237512 |
| C | -0.5174354845 | -1.0799549703 | 1.2734694358 |
| C | -1.9405269127 | -1.1019897633 | 1.1304230482 |
| N | 0.0591289249 | -1.2713852065 | 0.0435531053 |
| C | -2.2083203574 | -1.2075978310 | -0.2473829261 |
| C | -0.9651064007 | -1.1804003314 | -0.9284891112 |
| H | 0.9709436941 | -0.8610132770 | -0.1476246311 |
| H | -0.7951508588 | -1.6458473154 | -1.8930600964 |
| H | -3.1770882802 | -1.2622741340 | -0.7223197895 |
| C | 0.6033689465 | 1.8997234595 | 0.0423525065 |
| C | 1.8754181815 | 3.2928208506 | 1.4703465000 |
| C | 1.6467667119 | 4.4923757802 | 2.3700693021 |
| H | 2.2320515157 | 2.4206622889 | 2.0283727699 |
| C | 0.0941074588 | -0.9175984334 | 2.5299962448 |
| C | -2.7582325748 | -0.9914608757 | 2.2819313389 |
| C | -0.7362647666 | -0.8136200186 | 3.6310733060 |
| H | 1.1745543686 | -0.8828390169 | 2.6259148054 |
| C | -2.1517880706 | -0.8563965961 | 3.5123237733 |
| H | -3.8394104256 | -1.0001365903 | 2.1865710731 |
| H | -0.2966376233 | -0.6938421349 | 4.6171693795 |
| H | -2.7580526138 | -0.7657018767 | 4.4082619242 |
| O | 1.6131048067 | 1.2333815630 | -0.1922143001 |
| O | 0.6100146111 | 2.9707596894 | 0.8526741569 |
| H | 2.6103942941 | 3.5017801741 | 0.6857833049 |
| H | 2.5862364508 | 4.7773370600 | 2.8548180280 |
| H | 1.2817041030 | 5.3475517542 | 1.7943840585 |
| H | 0.9113407095 | 4.2630908996 | 3.1465215802 |

TS*exo*[**2a** + indole] – C2 alkylation

Imaginary frequency = -373 cm^–1^

| C | -0.0304975885 | 0.7688494886 | 0.3104042507 |
| --- | --- | --- | --- |
| C | -2.1921276135 | 1.5302107677 | -0.2720063798 |
| C | -2.8504355479 | 2.8167836583 | -0.7330586884 |
| H | -2.3145602358 | 0.7221231752 | -1.0013137797 |
| C | 1.3998806203 | 1.1032080986 | 0.4680957837 |
| C | 2.2620592155 | 0.1393133534 | 1.0136894506 |
| H | 1.7872602075 | -0.6952913982 | 1.5183993623 |
| H | 3.2073136343 | 0.5000760371 | 1.4045631056 |
| N | 1.8848595963 | 2.2478781664 | -0.1308619884 |
| O | 3.1387046580 | 2.3505640200 | -0.0917413833 |
| H | 2.1556899329 | -1.3110355752 | -0.8423939122 |
| C | 3.1028500655 | -0.9486977727 | -0.4612806473 |
| C | 3.9422064487 | -0.0077085087 | -1.1162059447 |
| N | 3.9530933754 | -1.8781393609 | 0.1727454178 |
| C | 5.2772162803 | -0.2811791271 | -0.7356000060 |
| H | 3.6083860071 | 0.7662666145 | -1.7878469870 |
| C | 5.2503737190 | -1.4434864821 | 0.0961042366 |
| H | 3.6282730162 | -2.6017732651 | 0.7949524625 |
| C | 6.5121394453 | 0.3503303110 | -1.0041923276 |
| C | 6.4176356708 | -1.9656741586 | 0.6722384563 |
| C | 7.6631627646 | -0.1759205049 | -0.4482233187 |
| H | 6.5445507949 | 1.2379658663 | -1.6282920235 |
| C | 7.6117196590 | -1.3216453818 | 0.3832758681 |
| H | 6.3903417125 | -2.8430324443 | 1.3114064862 |
| H | 8.6223098883 | 0.2947446048 | -0.6398401861 |
| H | 8.5349003388 | -1.7061287910 | 0.8073410787 |
| O | -0.4826907701 | -0.3453202960 | 0.5406586343 |
| O | -0.7845578882 | 1.8047755749 | -0.1044678758 |
| H | -2.6028748326 | 1.1758556985 | 0.6793250200 |
| H | -2.7163854310 | 3.6105036937 | 0.0075382562 |
| H | -3.9237368309 | 2.6550291624 | -0.8771039466 |
| H | -2.4209603126 | 3.1564547749 | -1.6799084747 |

TS*endo*[**2a** + **10**] – C2 alkylation

Imaginary frequency = -329 cm^–1^

| C | -0.6824401476 | 1.5411768276 | -0.4941376516 |
| --- | --- | --- | --- |
| C | -0.8349297091 | 0.6966561926 | -1.5968291302 |
| H | 0.0441226275 | 0.4828963094 | -2.1947071908 |
| H | -1.7893040116 | 0.7528620929 | -2.1081215841 |
| N | -1.7835517721 | 2.1006914817 | 0.1595030150 |
| O | -2.8869815566 | 1.7560300201 | -0.3094918621 |
| C | -0.5789929239 | -1.1803371870 | 1.2646159285 |
| C | -2.0033575493 | -1.2118214595 | 1.1052611942 |
| N | 0.0072762826 | -1.3497468051 | 0.0388282786 |
| C | -2.2512763223 | -1.3080932039 | -0.2738732178 |
| C | -1.0071948928 | -1.2658337391 | -0.9416884142 |
| H | -0.8163840842 | -1.6941388338 | -1.9183538032 |
| C | 0.6589668228 | 1.7526221096 | 0.0666377939 |
| C | 2.0397980349 | 3.0190187663 | 1.5153335463 |
| C | 1.8875038424 | 4.1869195608 | 2.4716789061 |
| H | 2.3649302085 | 2.1079390676 | 2.0267216974 |
| C | -0.0061759799 | -1.0373915391 | 2.5433090655 |
| C | -2.8610349202 | -1.1286081083 | 2.2387724622 |
| C | -0.9073135203 | -0.9682293870 | 3.6308847485 |
| C | -2.3186992207 | -1.0150082554 | 3.4972288289 |
| H | -3.9372566612 | -1.1460378392 | 2.1014008502 |
| H | -2.9541746103 | -0.9490068585 | 4.3753577782 |
| H | -3.2159871722 | -1.3578727592 | -0.7588739053 |
| H | 0.9153352288 | -0.9286376267 | -0.1495287907 |
| O | 1.6277130133 | 1.0429140435 | -0.2202994505 |
| O | 0.7451344769 | 2.7889587572 | 0.9147027844 |
| H | 2.7653212374 | 3.2304817939 | 0.7225870153 |
| H | 2.8491030427 | 4.4069793000 | 2.9469171895 |
| H | 1.5493024820 | 5.0827996977 | 1.9433443372 |
| H | 1.1592402442 | 3.9554767891 | 3.2540715362 |
| N | -0.1730242054 | -0.8566390900 | 4.7929920968 |
| C | 1.3299028853 | -0.9528706300 | 3.0885367022 |
| H | -0.5654812486 | -0.7694864907 | 5.7167218190 |
| C | 1.1819450225 | -0.8428162197 | 4.4963965069 |
| C | 2.2772254923 | -0.7373711914 | 5.3570492032 |
| C | 2.6252764997 | -0.9464184243 | 2.5485251496 |
| C | 3.5598035786 | -0.7408285240 | 4.8075242267 |
| C | 3.7139228182 | -0.8419395083 | 3.4056908222 |
| C | 4.7801301551 | -0.6434571114 | 5.6935425384 |
| H | 5.3544725311 | -1.5779987987 | 5.6836636947 |
| H | 5.4551949992 | 0.1503189922 | 5.3546034861 |
| H | 4.5082244012 | -0.4327627975 | 6.7318334939 |
| H | 2.1366456659 | -0.6531856913 | 6.4317557731 |
| H | 4.7174747563 | -0.8332884861 | 2.9879169690 |
| H | 2.7820941587 | -0.9963152372 | 1.4760955622 |

TS*exo*[**2a** + **10**] – C2 alkylation

Imaginary frequency = -338 cm^–1^

| C | 0.0178213701 | 0.5531935333 | 0.1911777762 |
| --- | --- | --- | --- |
| C | -2.1860312367 | 1.0442901175 | -0.5093973417 |
| C | -2.9611066565 | 2.2320155091 | -1.0489563938 |
| H | -2.1228707192 | 0.2283699084 | -1.2366346861 |
| C | 1.3715799845 | 1.0650904242 | 0.4731205149 |
| C | 2.3279696026 | 0.1806982664 | 0.9896740885 |
| H | 1.9543948204 | -0.7436265864 | 1.4161463689 |
| H | 3.2195742290 | 0.6204022560 | 1.4217682872 |
| N | 1.7116557136 | 2.3289752112 | 0.0215021398 |
| O | 2.9319564886 | 2.6037847627 | 0.1361744096 |
| H | 2.3912979326 | -1.1437730802 | -0.9676323791 |
| C | 3.3117309204 | -0.7464193663 | -0.5587932545 |
| C | 4.0771388130 | 0.3151994659 | -1.1010842411 |
| N | 4.2283335555 | -1.6560363646 | 0.0059060883 |
| C | 5.4245295166 | 0.1142269327 | -0.7371434531 |
| C | 5.4876644067 | -1.1273859709 | -0.0263068000 |
| C | 6.5994943095 | 0.8893344027 | -0.9340808814 |
| C | 6.7051062183 | -1.5985355416 | 0.4930103125 |
| C | 7.8071330872 | 0.4420721945 | -0.4446348842 |
| H | 6.5353109295 | 1.8344328386 | -1.4635542300 |
| C | 7.8423052595 | -0.7878560035 | 0.2567388252 |
| H | 8.7157556579 | 1.0198232352 | -0.5831456806 |
| H | 3.6749937475 | 1.1484512237 | -1.6552546756 |
| H | 3.9608241362 | -2.4830297837 | 0.5147086087 |
| O | -0.2844537226 | -0.6300174199 | 0.2949880523 |
| O | -0.8544882028 | 1.5049475976 | -0.1994819991 |
| H | -2.6442364075 | 0.6364997720 | 0.3985166508 |
| H | -3.0113406064 | 3.0353877179 | -0.3082983196 |
| H | -3.9826089838 | 1.9276130879 | -1.2994568945 |
| H | -2.4860732779 | 2.6283687121 | -1.9511321541 |
| N | 8.9367360105 | -1.4084630824 | 0.8226150598 |
| C | 7.1506473689 | -2.7557517804 | 1.2383634470 |
| C | 8.5501971498 | -2.5971941195 | 1.4239024308 |
| H | 9.8764651916 | -1.0455823991 | 0.8075925349 |
| C | 9.3244513710 | -3.5373617930 | 2.1043750762 |
| C | 6.5359488000 | -3.9022649907 | 1.7603943103 |
| C | 7.3066835216 | -4.8390300301 | 2.4398899088 |
| C | 8.6969493385 | -4.6721600958 | 2.6232847700 |
| C | 9.4900488639 | -5.6988459114 | 3.3983663236 |
| H | 9.4632995512 | -5.4907871142 | 4.4756839000 |
| H | 9.0882940708 | -6.7064485790 | 3.2536108658 |
| H | 10.5411575430 | -5.7077208390 | 3.0941949577 |
| H | 10.3947653870 | -3.3935519046 | 2.2294015728 |
| H | 6.8292919081 | -5.7284127689 | 2.8424276078 |
| H | 5.4697030386 | -4.0730216444 | 1.6373533801 |

TS*endo*[**2a** + **10**] – C3 alkylation

Imaginary frequency = -330 cm^–1^

| C | -0.2403575613 | 1.9379224453 | -0.8885256319 |
| --- | --- | --- | --- |
| C | -0.3529212291 | 0.8439852373 | -1.7648319137 |
| H | 0.5669410063 | 0.3457554191 | -2.0489572374 |
| H | -1.1453528891 | 0.9120536041 | -2.5019459047 |
| N | -1.3362468118 | 2.6922873851 | -0.5453729119 |
| O | -2.4181551409 | 2.2953274908 | -1.0660276212 |
| C | -0.8985502022 | -0.4993349404 | 1.4095407410 |
| C | -0.3368753998 | -1.1255169769 | 0.2809474224 |
| N | -2.1176710119 | 0.0608901906 | 1.0130915460 |
| C | -1.2103624846 | -0.8091836865 | -0.8511665346 |
| C | -2.3506726249 | -0.1757094362 | -0.2948785333 |
| H | -2.6977862579 | 0.6543020797 | 1.5871255699 |
| H | -3.2699699046 | 0.1265231682 | -0.7660706763 |
| C | 1.0578640467 | 2.2211902297 | -0.2430925846 |
| C | 2.2788547144 | 3.6446907346 | 1.1943307798 |
| C | 2.0287369846 | 4.8573313238 | 2.0714672248 |
| H | 2.6413148779 | 2.7878116291 | 1.7719577098 |
| C | -0.2764650602 | -0.5380341170 | 2.6635320341 |
| C | 0.8783644885 | -1.8230951448 | 0.3891564348 |
| C | 0.9416751341 | -1.2658438803 | 2.7268760058 |
| C | 1.5189831392 | -1.9079545411 | 1.6190324471 |
| H | 1.3178798357 | -2.2963347373 | -0.4834640552 |
| H | 2.4532800434 | -2.4514925627 | 1.7202492101 |
| H | -1.2643260211 | -1.4007090575 | -1.7565474492 |
| O | 2.0672338995 | 1.5592056745 | -0.4328849705 |
| O | 1.0258061315 | 3.2995873103 | 0.5711046832 |
| H | 3.0221252255 | 3.8488213418 | 0.4157858848 |
| H | 2.9599561344 | 5.1635157091 | 2.5597863714 |
| H | 1.6564080088 | 5.6972615681 | 1.4778641543 |
| H | 1.2900810120 | 4.6322760872 | 2.8464030888 |
| N | 1.4025385335 | -1.2134177605 | 4.0332210098 |
| H | 2.2615358607 | -1.6264737971 | 4.3577793518 |
| C | 0.5325120552 | -0.4675956928 | 4.8103300689 |
| C | -0.5393073040 | -0.0248693051 | 3.9896426770 |
| C | -1.5489231941 | 0.7563031533 | 4.5701007353 |
| C | 0.6056323480 | -0.1509096925 | 6.1689402383 |
| C | -1.4748236953 | 1.0726061558 | 5.9207595272 |
| C | -0.4057451794 | 0.6293609832 | 6.7316956760 |
| C | -0.3520943828 | 1.0229794902 | 8.1899330734 |
| H | -1.3281634301 | 0.9028179079 | 8.6719226932 |
| H | 0.3740714248 | 0.4198079639 | 8.7426122914 |
| H | -0.0632809958 | 2.0753304671 | 8.3054854487 |
| H | 1.4343937794 | -0.4999269319 | 6.7796799797 |
| H | -2.3856232883 | 1.1194644501 | 3.9790947607 |
| H | -2.2570146150 | 1.6785930604 | 6.3702171849 |

TS*exo*[**2a** + **10**] – C3 alkylation

Imaginary frequency = -322 cm^–1^

| C | -0.0676920827 | 0.6651314234 | 0.5376807861 |
| --- | --- | --- | --- |
| C | -2.4123450053 | 0.9510686886 | 0.5183788092 |
| C | -3.4170726287 | 2.0871782570 | 0.5155522060 |
| H | -2.5174697703 | 0.3074535240 | -0.3618426046 |
| C | 1.2615889590 | 1.3081057783 | 0.5294406905 |
| C | 2.3996281135 | 0.5045915650 | 0.6999064199 |
| H | 2.2453434431 | -0.5098716028 | 1.0471949172 |
| H | 3.3096745598 | 1.0085600334 | 1.0033852304 |
| N | 1.3599931010 | 2.6306051745 | 0.1641814768 |
| O | 2.5460402058 | 3.0279808576 | -0.0139304798 |
| C | 3.5923121552 | 1.1803705943 | -1.6331530676 |
| C | 3.1428844700 | -0.0727091031 | -1.1512952755 |
| N | 4.9407248403 | 1.2405729955 | -1.5260700724 |
| C | 4.3547402580 | -0.8370946367 | -0.8536370160 |
| C | 5.4479323437 | 0.0338345205 | -1.0408603665 |
| C | 4.5666070791 | -2.1496432094 | -0.3937214897 |
| C | 6.7647300088 | -0.3645466726 | -0.7774508114 |
| C | 5.8582682608 | -2.5895549326 | -0.1294568849 |
| H | 3.7242387295 | -2.8194782264 | -0.2494972295 |
| C | 6.9286619818 | -1.6993815607 | -0.3191351049 |
| H | 6.0368585516 | -3.6031840136 | 0.2165362831 |
| H | 2.2033580048 | -0.5175775826 | -1.4507777022 |
| H | 5.4811931622 | 2.0777949067 | -1.6813499621 |
| H | 3.0284152873 | 2.0105441137 | -2.0238865612 |
| O | -0.2213358227 | -0.5488814380 | 0.5621723459 |
| O | -1.0949530210 | 1.5397057759 | 0.5167154543 |
| H | -2.5174060399 | 0.3114729472 | 1.4010633452 |
| H | -3.2969908268 | 2.7171856708 | 1.4018307483 |
| H | -4.4352091755 | 1.6841591470 | 0.5135056484 |
| H | -3.2928986276 | 2.7175553565 | -0.3699824327 |
| N | 8.2824596399 | -1.9194766862 | -0.1195153249 |
| C | 8.0821100014 | 0.2273753583 | -0.8528519315 |
| C | 8.9965749120 | -0.7757913500 | -0.4333214650 |
| H | 8.6846714060 | -2.7829031005 | 0.2070900507 |
| C | 10.3736773702 | -0.5482852102 | -0.3775025798 |
| C | 8.5887116611 | 1.4820999205 | -1.2216533291 |
| C | 9.9583379957 | 1.7072872101 | -1.1677824214 |
| C | 10.8627169158 | 0.7044578510 | -0.7505320397 |
| H | 10.3491251916 | 2.6810136132 | -1.4509545684 |
| H | 7.9269978436 | 2.2816710153 | -1.5450939701 |
| H | 11.0544167179 | -1.3297804983 | -0.0493425644 |
| C | 12.3468469625 | 0.9878447279 | -0.7315778210 |
| H | 12.8922147941 | 0.2366784724 | -0.1530360871 |
| H | 12.7637354231 | 0.9888595810 | -1.7466442008 |
| H | 12.5596826503 | 1.9696007440 | -0.2955790479 |
